# Synthesis, Antibacterial Evaluation, and Chemometric Profiling of a Vanilloid-Based Compounds Library Active Against *Helicobacter pylori*

**DOI:** 10.3390/antibiotics15080729

**Published:** 2026-07-27

**Authors:** Ilaria D’Agostino, Strahinja Kovačević, Moataz A. Shaldam, Alessandra Ammazzalorso, Cristina Campestre, Paolo Guglielmi, Michele Coluccia, Anna Gilardoni, Okan Aykaç, İrem Bozbey Merde, Francesca Sisto, Simone Carradori

**Affiliations:** 1 Department of Pharmacy, University of Pisa, via Bonanno 6, 56126 Pisa, Italy; ilaria.dagostino@unipi.it; 2 Faculty of Technology Novi Sad, University of Novi Sad, Bulevar cara Lazara 1, 21000 Novi Sad, Serbia; strahko@uns.ac.rs; 3 Department of Pharmacy, “G. d’Annunzio” University of Chieti-Pescara, via dei Vestini 31, 66100 Chieti, Italy; dr_moutaz_986@pharm.kfs.edu.eg (M.A.S.); alessandra.ammazzalorso@unich.it (A.A.); cristina.campestre@unich.it (C.C.); simone.carradori@unich.it (S.C.); 4 Department of Drug Chemistry and Technologies, Sapienza University of Rome, P.le A. Moro 5, 00185 Rome, Italy; paolo.guglielmi@uniroma1.it (P.G.); michele.coluccia@uniroma1.it (M.C.); 5 Department of Biomedical, Surgical and Dental Sciences, University of Milan, Via Pascal 36, 20133 Milan, Italy; piera.gilardoni@unimi.it; 6 Department of Pharmaceutical Chemistry, Faculty of Pharmacy, Sivas Cumhuriyet University, 58000 Sivas, Turkey; okanaykac@cumhuriyet.edu.tr; 7 Department of Pharmaceutical Chemistry, Institute of Health Sciences, Inonu University, 44000 Malatya, Turkey; 8 Department of Pharmaceutical Chemistry, Faculty of Pharmacy, Inonu University, 44000 Malatya, Turkey; irem.bozbey@inonu.edu.tr

**Keywords:** vanillin, *Helicobacter pylori*, MIC, MBC, hierarchical cluster analysis, artificial neural networks, time-killing, GES-1, molecular docking

## Abstract

**Background:** Among natural products, vanillin (**Van**), a major component of *Vanilla planifolia*, exhibits multiple bioactivities, including antimicrobial effects. **Methods:** In this study, **Van**, its analogues *o*-vanillin (**oVan**), *iso*-vanillin (**iVan**), ethylvanillin (**eVan**), and a library of newly synthesized derivatives were evaluated against *Helicobacter pylori* strains with distinct antibiotic susceptibilities. Time-kill kinetics, antibacterial spectrum, and viability in a normal gastric cell line GES-1, were also assessed. **Results: Van** showed minimal or no activity (MIC and MBC > 128 µg/mL), whereas structural modifications markedly improved anti-*H. pylori* activity, with MIC values as low as 4 µg/mL. Compounds **16V**, **20oV,** and **29eV** were among the most potent (MIC_90_ = 4–16 µg/mL). Activity depended on both the vanilloid core and substituent type. The compounds were inactive against representative Gram-negative and Gram-positive bacteria (MIC > 128 µg/mL). Selected compounds preserved viability in GES-1 cells. Hierarchical clustering, artificial neural clustering, and principal component analysis identified potency-related architectural motifs and strain-specific activity. Docking against *H. pylori* urease suggested that several compounds, particularly **16V**, may interact with the enzyme, providing preliminary support for a possible involvement of this target. **Conclusions:** Systematic modification of the vanilloid scaffold generated selective and relatively non-cytotoxic anti-*H. pylori* hit compounds and confirmed the value of natural metabolites in antibacterial drug discovery.

## 1. Introduction

**Figure 1:**
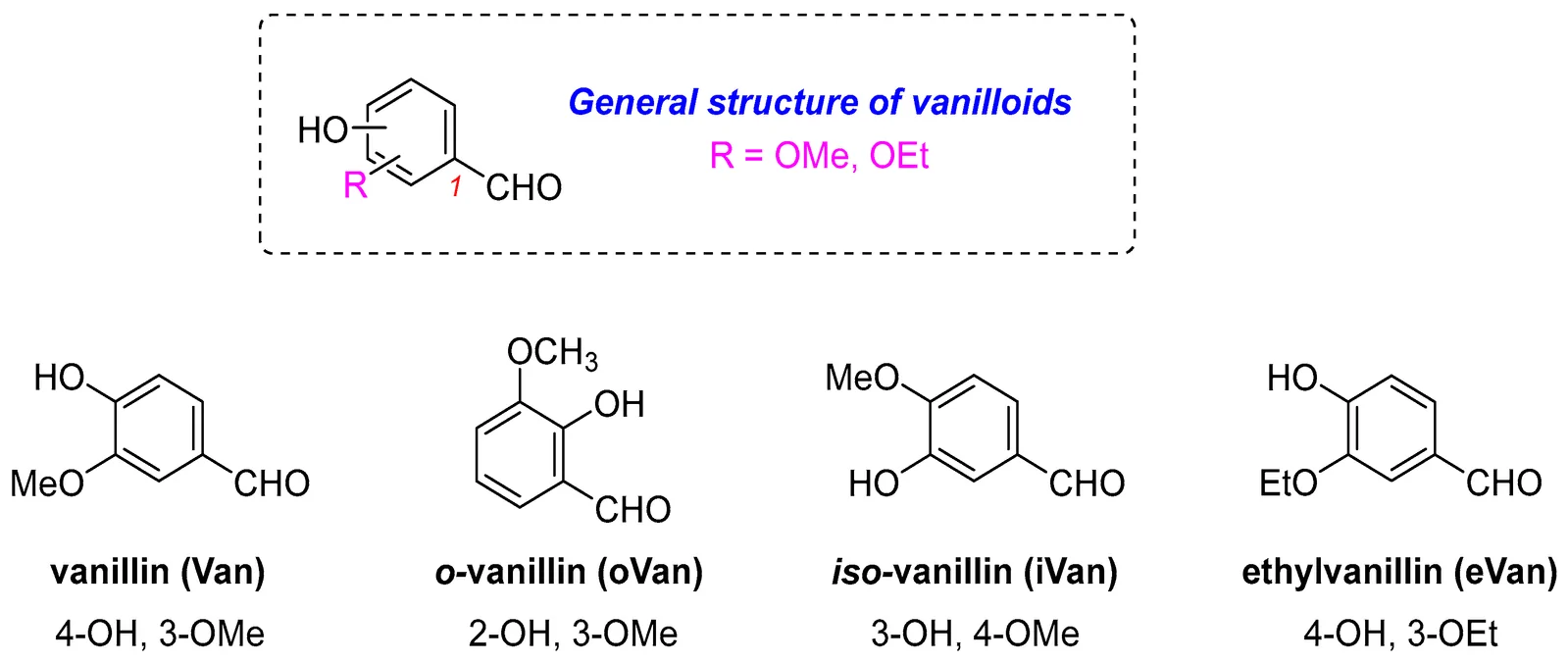
Chemical structure of vanilloids, with **Van** and **oVan** previously investigated as anti-*H. pylori* agents [7].

Nature has long served as a rich source of bioactive compounds with diverse health-promoting properties, offering valuable hit compounds for the development of new therapeutics or prophylactic agents [1,2,3]. In this context, the vanilla bean (*Vanilla planifolia*) has been recognized for its multifaceted bioactivities and application in traditional medicine, besides its common use as a flavoring and preservative agent in the food industry [4]. The vanilla plant contains several chemical constituents, including 4-hydroxy-3-methoxybenzaldehyde, commonly known as “vanillin” (**Van**) (Figure 1), a major aromatic component associated with main beneficial effects reported for vanilla extracts [5,6].

This compound was demonstrated to possess polypharmacological properties, including antimicrobial [8,9,10], antioxidant [11,12], anti-inflammatory [13], and anticancer activities [14]. **Van** isomers, such as *o*-vanillin (**oVan**) [15] and *iso*-vanillin (**iVan**) [16] (Figure 1), have been detected in trace amounts in certain plant species and are also produced industrially, serving as ingredients in flavorings and fragrances, together with the fully synthetic analogue ethylvanillin (**eVan**) (Figure 1) [17]. Despite their close structural similarity to **Van**, these compounds were poorly exploited for their antimicrobial potential [18,19,20,21]. Therefore, as part of this study, we investigated their possible use as antibacterial agents, focusing on their activity against *Helicobacter pylori* (*H. pylori*) and the development of related derivatives.

Briefly, *H. pylori* is a microaerophilic, Gram-negative, spiral-shaped bacterium infecting about half the global population [22,23]. This bacterium can survive in the harsh gastric environment through the combined activity of urease and carbonic anhydrases [24,25], along with its peculiar motility and adhesion features [26,27]. *H. pylori* infection causes gastric and duodenal ulcers, and mucosa-associated lymphoid tissue (MALT) lymphoma. Also, it is associated with a significant risk factor for the development of gastric cancer, with approximately 1% of infected individuals developing the disease [22,28,29]. For these reasons, the International Agency for Research on Cancer (IARC), part of the World Health Organization (WHO), has classified *H. pylori* as a Group 1 carcinogen [30]. Indeed, *H. pylori* is the most common human bacterial pathogen occurring in 40–60% the world population. In more detail, a recent comprehensive study assessed that the global prevalence of *H. pylori* infection slightly decreased to 43.9% in adults over the last three decades while still being as high as 35.1% in children [31,32].

The standard therapeutic approach to eradicate *H. pylori* infection traditionally involved a triple therapy based on clarithromycin, amoxicillin, and a proton pump inhibitor (PPI) [33,34]. However, clarithromycin resistance rates exceeding 20% in the target population and/or the presence of penicillin allergy significantly restrict the effectiveness of this regimen, in favour of bismuth-based quadruple therapy (BQT) or non-bismuth quadruple therapy [26]. Current guidelines recommend 14-day optimized BQT as the preferred empirical regimen when antibiotic susceptibility is unknown. Factors such as the antibiotic resistance, inadequate acid suppression, and poor patient adherence can markedly compromise treatment success [35]. Hence, the need for new antibacterial agents is pressing, and in the search for new bioactive compounds, natural products undoubtedly represent a promising starting point [36,37,38,39,40,41].

**Figure 2:**
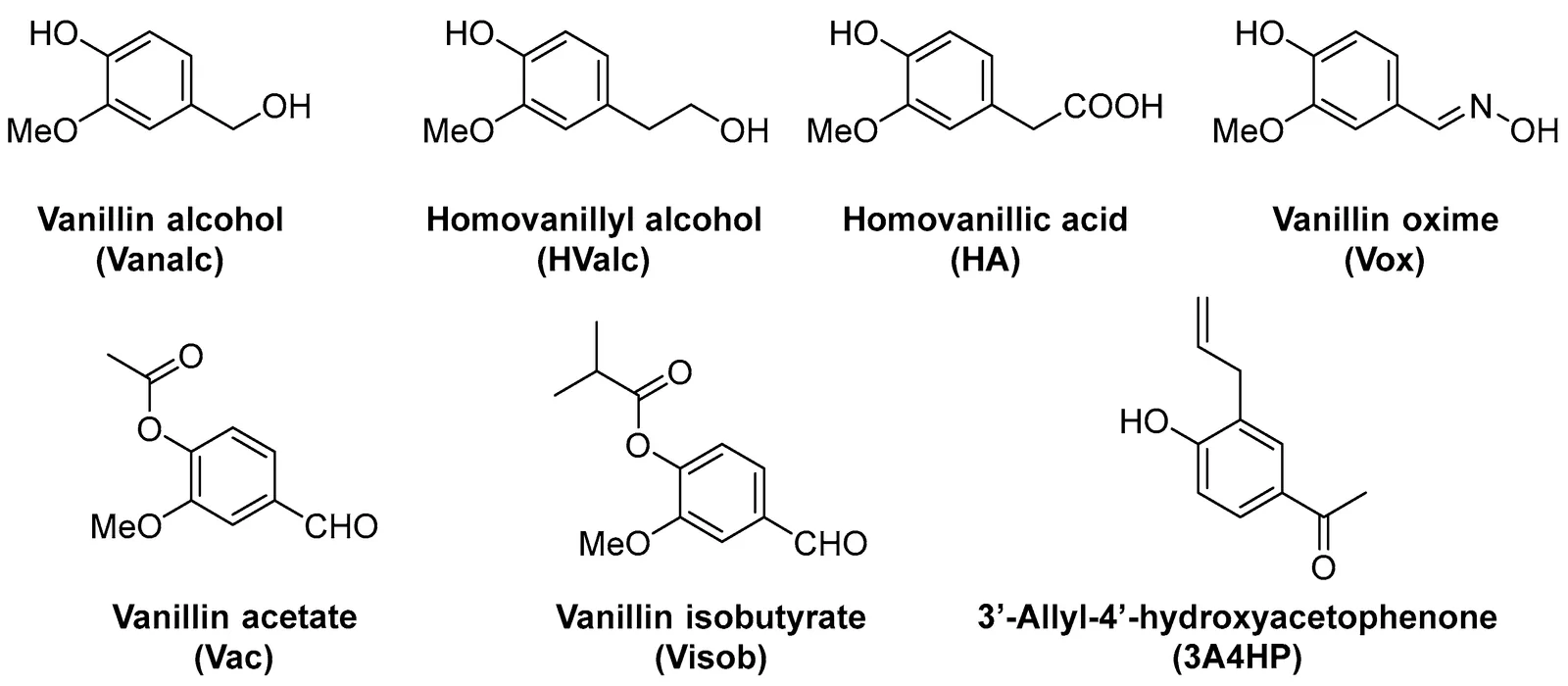
Chemical structure of commercially available vanilloid compounds investigated in this work.

**Figure 3:**
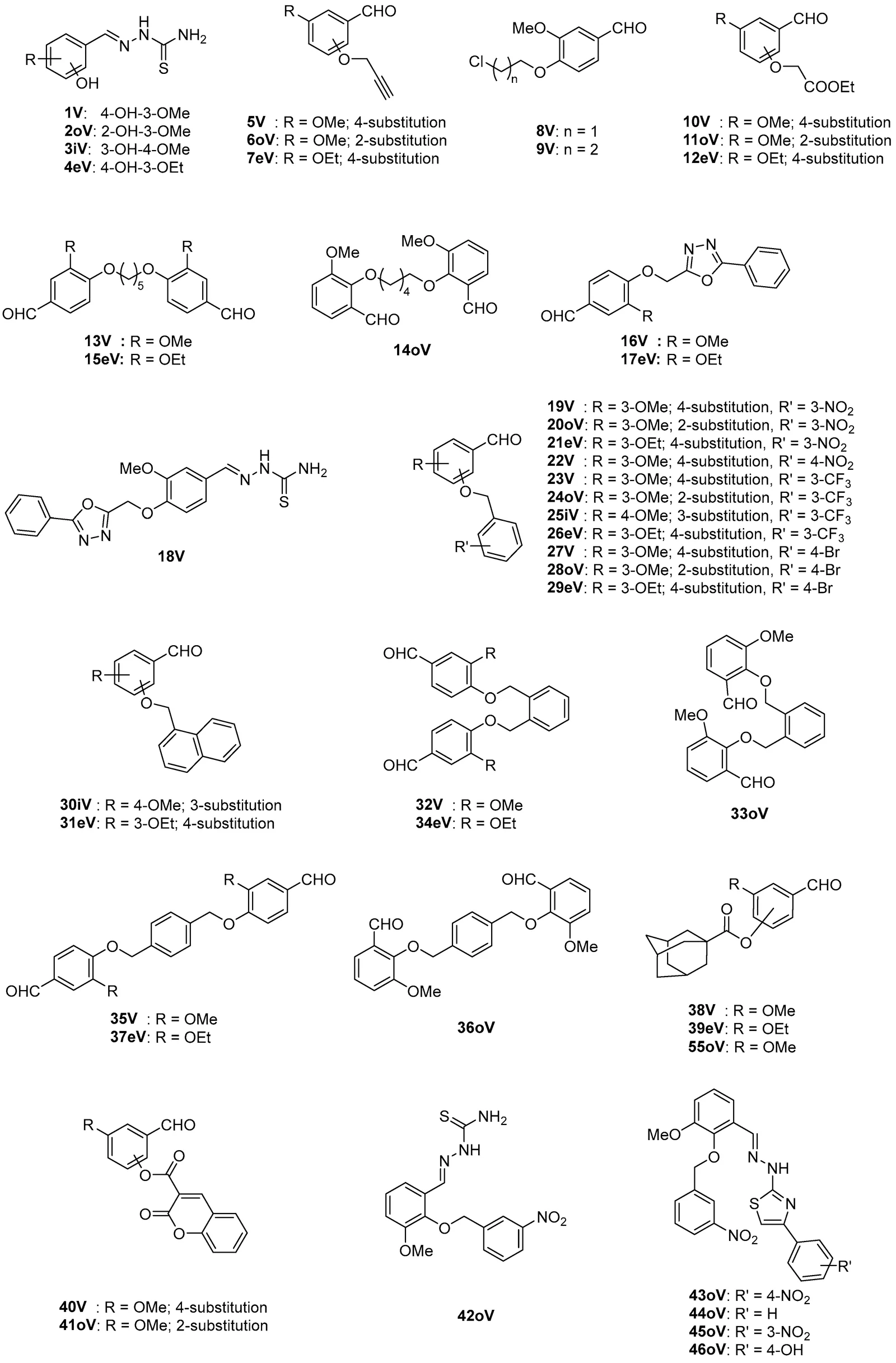
Chemical structure of new vanilloid derivatives developed in this work.

In this frame, we have previously investigated several natural phenols, including the isomers carvacrol and thymol, eugenol, and some vanilloids (Figure 1), along with their derivatives obtained via alkylation, benzylation, or azotization [42,43,44,45,46]. Herein, for the first time, we explored commercially available **Van** analogues (Figure 2) and chemical modifications of such vanilloids (Figure 3) to determine how to improve anti-*H. pylori* activity by testing them against *H. pylori* strains with different antibiotic susceptibility phenotypes.

To complement the biological evaluation, multivariate analyses were applied to explore patterns within the MIC and MBC datasets [47]. These chemometric tools enabled the identification of similarity relationships among compounds and strains, the detection of non-linear clustering behavior, and the visualization of the variables driving activity differences, providing a comprehensive framework for interpreting antibacterial profiles within the vanilloid library [48]. Lastly, an exploratory in silico analysis was performed to assess the putative interactions with *H. pylori* urease, a key enzyme for the bacterium survival, as a putative target for this series of compounds [49,50].

## 2. Results and Discussion

### 2.1. Design and Synthesis of the Vanilloid Library

**Scheme 1:**
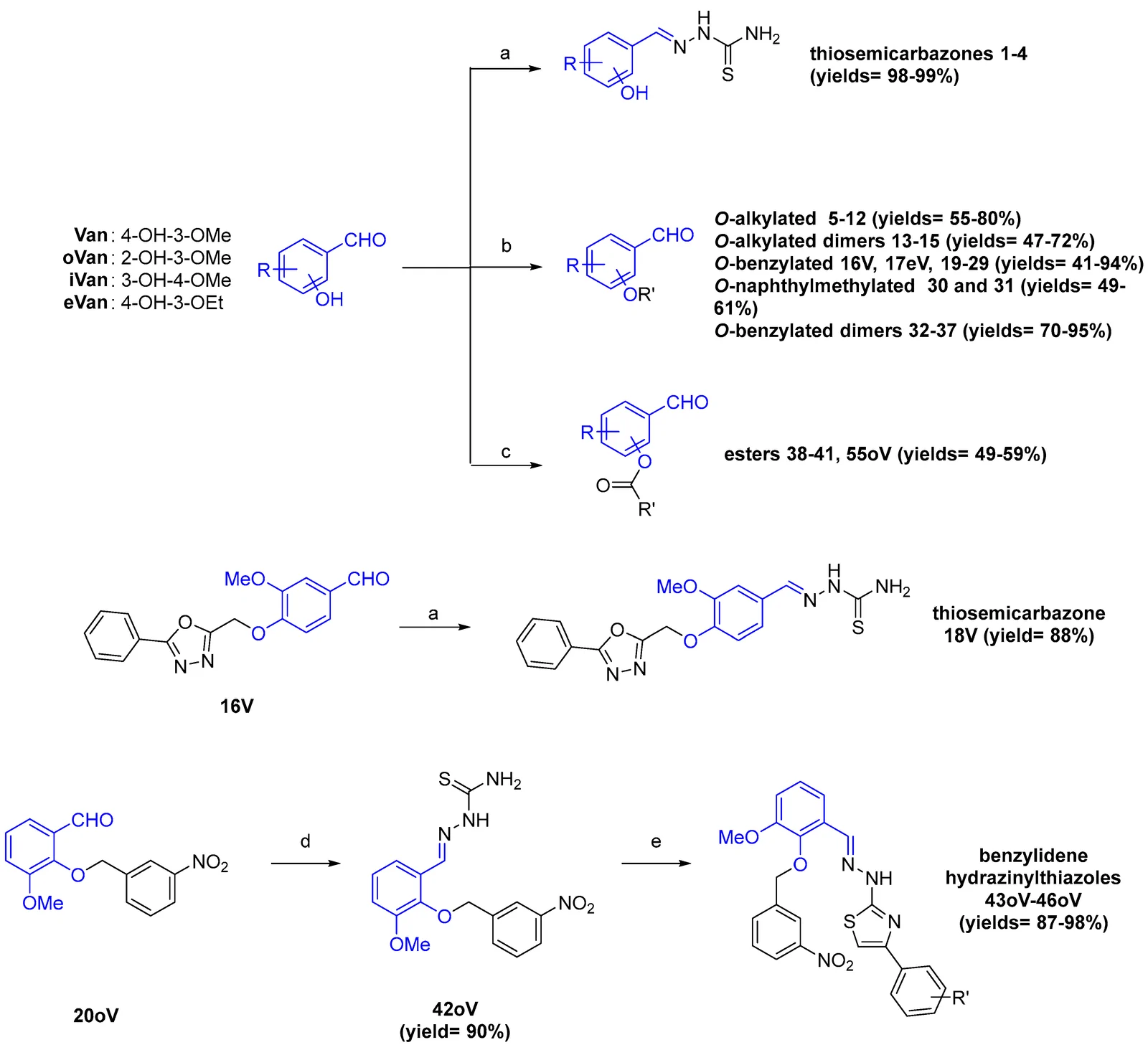
Synthesis of vanilloid derivatives herein reported. *Reagents and conditions*: (a) thiosemicarbazide, MW, 110 °C, 15 min; (b) proper halide, K_2_CO_3_, refluxing ACN, 16 h; (c) adamantane-1-carbonyl chloride, TEA, DMAP, DCM, 0–r.t., 48 h or coumarin-3-carboxylic acid, DCC, DMAP, dry DCM, r.t., 16 h; (d) thiosemicarbazide, absolute ethanol, reflux, 4 h; (e) proper phenacyl derivative, abs. ethanol, reflux, 3–6 h. Yields are reported for each chemical group within brackets.

To explore the chemical space of the vanilloid scaffold, a series of modifications was carried out targeting its two key functional moieties: the phenolic group and the aldehyde group (Scheme 1).

In addition to these chemical modifications, isomeric variations were also investigated by designing derivatives based on **Van**, **iVan**, and **oVan** substitution patterns. Furthermore, ethyl **Van** was included as an additional parent compound for the design. Specifically, the reactive aldehyde group was transformed into thiosemicarbazones, functional moieties well-known in medicinal chemistry for their versatility and diverse biological activities. Indeed, these compounds are able to form additional hydrogen bonds and coordinating interactions with biological targets, potentially enhancing binding affinity and modulating pharmacological activity [51]. Accordingly, thiosemicarbazones **1**–**4** were designed and synthesized through a reaction between the aldehyde group of the vanilloid scaffold and thiosemicarbazide, performed under microwave irradiation at 110 °C for 15 min.

Regarding the phenolic group, it was *O*-modified via alkylation and benzylation reactions, yielding compounds **5**–**17** and **19**–**37** through nucleophilic substitution of the corresponding vanilloid with the appropriate alkyl bromide in refluxing acetonitrile. Specifically, among the *O*-alkylated vanilloids, the propargylated **5**–**7**, the chloroethyl **8V**, and the chloropropyl **9V** were designed to introduce reactive and synthetically versatile tails. In particular, in some cases they may act as electrophilic sites, capable of forming covalent bonds with nucleophilic residues in target proteins, thereby functioning as alkylating agents. In addition, these groups can provide a handle for further chemical functionalization, such as through Huisgen click chemistry [52] or nucleophilic substitution reactions. In contrast, the esterified carboxymethyl tails in compounds **10**–**12** were introduced to mask free carboxylic acid function and allow its release via hydrolysis, potentially enhancing target interactions while modulating solubility and cellular uptake [53]. Interestingly, symmetric dimers of **Van**, **oVan**, and **eVan** were designed by introducing a five-methylene spacer between the two cores and derivatizing the phenolic functions, yielding derivatives **13**–**15**. This dimerization strategy was intended to explore the potential of the two benzaldehyde moieties to interact with targets while reducing steric hindrance, thanks to the flexibility provided by the medium-length spacer [54].

Additionally, the 5-phenyl-1,3,4-oxadiazol-2-ylmethoxy substituent was introduced on the phenolic group of **Van** and **eVan**, yielding **16V** and **17eV**, to incorporate an aromatic moiety, the oxadiazole, capable of establishing hydrogen bonds and polar interactions, thereby enhancing target engagement and contributing to the overall pharmacophoric profile of the scaffold [55]. Also, the aldehyde moiety of compound **16V** was converted into a thiosemicarbazone, affording **18V**.

The large series of benzyl derivatives **19**–**29** explored distal phenyl functionalization by introducing either 3- or 4-nitro groups (**19**–**21** and **22V**, respectively), a 3-trifluoromethyl substituent (**23**–**26**), or a 4-bromo atom (**27**–**29**) onto the various vanilloid cores. These substituents were selected based on medicinal chemistry considerations, as they modulate electronic properties, lipophilicity, and potential for additional interactions. Moreover, these substituents were also chosen due to the ready availability of the corresponding benzyl bromides in the laboratory, facilitating straightforward synthesis and broad analog generation.

As a continuation of the series, we also decorated the phenolic moiety of **iVan** and **eVan** with a 1-naphthylmethyl tail in derivatives **30iV** and **31eV**, respectively, thereby increasing the lipophilicity of the resulting compounds and enhancing the possibility of establishing hydrophobic and π-stacking interactions within non-polar regions of the target binding site [56]. Moreover, a focused library of dimers was designed through strategic *O*-derivatization of the phenolic moieties [57]. Specifically, various spacers, such as 1,2-benzenedimethanol (phthalyl alcohol) and 1,4-benzenedimethanol, were employed to modulate the distance and orientation between vanilloid units, introduced via *O*-benzylation with **Van**, **oVan**, or **eVan**, affording compounds **32**–**34** and **35**–**37**, respectively, to investigate how spacer length and substitution pattern influence biological activity.

A series of ester derivatives was developed to evaluate the effect of steric and electronic modifications at the phenolic hydroxyl group. These included 1-adamantanecarboxylic acid esters of **Van** (**38V**), **oVan** (**55oV**), and **eVan** (**39eV**), as well as 3-coumarin carboxylic acid esters of **Van** (**40V**) and **oVan** (**41oV**), designed to explore potential improvements in lipophilicity, membrane permeability, and target engagement [58]. Finally, *O*-3-nitrobenzylated **oVan** was further elaborated to generate thiosemicarbazone **42oV** and a series of benzylidene hydrazinylthiazoles (**43oV**–**46oV**). In these thiazole derivatives, none or different phenyl substituents (3- or 4-nitro, 4-hydroxy) were introduced at position C4 of the thiazole ring, aiming to systematically probe the impact of electronic and hydrogen-bonding modifications on bioactivity. Overall, the design and synthesis of this large library of vanilloid derivatives was aimed at exploring how structural variations, such as spacer length, phenolic *O*-derivatization, and thiosemicarbazone formation, affect biological activity, providing a rational framework to identify key molecular features underlying pharmacological effects.

### 2.2. In Vitro Antibacterial Activity Evaluation Against H. pylori Strains

The whole library of vanilloids was tested against four *H. pylori* strains with distinct antibiotic susceptibility profiles, including the NCTC 11637 reference strain and the clinical isolates F1, 23, and F40/499. These strains had been previously verified by Gram staining and systematically characterized with respect to catalase, urease, and oxidase enzymatic activities, thereby confirming their taxonomic identity and functional profile. Subsequent antimicrobial susceptibility testing was carried out using three clinically relevant antibiotics, metronidazole (MTZ), clarithromycin (CLR), and amoxicillin (AMX), chosen as benchmark agents given their widespread use in therapeutic regimens against *H. pylori* infection [59]. In line with previous reports, MTZ resistance is commonly associated with mutational inactivation of genes encoding oxygen-independent NADPH nitroreductases [60], enzymes involved in drug activation. Conversely, CLR resistance is commonly cassociated with mutations in the ribosomal 23S rRNA macrolide-bindig site [61], which reduce effective drug binding.

In Table 1, the minimum inhibitory concentration (MIC) and minimum bactericidal concentration (MBC) values are reported for **Van**, commercially available **Van** analogues, and the newly synthesized library of vanilloids. Additionally, MIC_50_, MIC_90_, and MBC_90_ values were provided. MIC_50_ and MIC_90_ represent the concentrations required to inhibit 50% and 90% of the tested *H. pylori* strains, respectively. Thus, taken together, these data provide not only a quantitative measure of growth inhibition but also insights into the bactericidal potential of the compounds, thereby offering a more comprehensive evaluation of their putative therapeutic relevance and effectiveness across the tested strain panel.

Observing the data in Table 1, **Van** was largely inactive against all tested *H. pylori* strains, with MIC and MBC values consistently >128 µg/mL. Analysis of commercially available derivatives featuring structural modifications revealed specific trends.

- **Van** isomerization: **oVan** exhibited markedly improved antibacterial activity compared to **Van**, with MIC and MBC values ranging from 4 to 16 µg/mL, whereas **iVan** remained inactive (MIC ≥ 128 µg/mL), thereby indicating that an ortho-positioned hydroxyl group enhances bacterial growth inhibition.
- The **Van** homologue **eVan** retained poor activity (MIC ≥ 128 µg/mL), suggesting that the introduction of a methylene unit is tolerated but insufficient to significantly enhance the antibacterial properties of the **Van** core.
- Reduction or oxidation of the aldehyde group was not a suitable modification to improve bioactivity. In fact, reduction in the aldehyde moiety, as in Vanillin alcohol (**Vanalc**) and Homovanillyl alcohol (**HValc**), did not improve activity (MIC ≥ 128 µg/mL), and, similarly, oxidation to the corresponding carboxylic acid, as in Homovanillic acid (**HA**), showed no enhancement over **Van**.
- Aldehyde modification, as the conversion to an oxime, as in Vanillin oxime (**Vox**), led to moderate activity (MIC 32 µg/mL; MBC 32–64 µg/mL).
- Esterification showed different results: Vanillin acetate (**Vac**) proved ineffective in enhancing activity (MIC ≥ 128 µg/mL), whereas Vanillin isobutyrate (**Visob**) showed improved activity (MIC 32–64 µg/mL; MBC 32–64 µg/mL), suggesting that introduction of small lipophilic ester groups may increase efficacy.
- Other modifications, such as 3′-allyl-4′-hydroxyacetophenone (**3A4HP**), also displayed moderate activity (MIC 16–32 µg/mL; MBC 16–32 µg/mL), indicating that additional aromatic and hydroxyl substituents can positively affect antibacterial properties.

Analysis of the data reported in Table 1 for the newly synthesized vanilloids revealed distinct structure–activity relationships (SARs), which were dependent on the specific series of derivatives examined. Within the thiosemicarbazone series (**1**–**4**), only the **Van**-derived one (**1V**) showed appreciable antibacterial activity, with MIC and MBC values of 16–32 and 32–64 µg/mL, respectively, across the tested strains. Thus, **1V** represents a significant improvement compared to **Van**, indicating that that the effect of aldehyde conversion into a thiosemicarbazone scaffold is strongly dependent on the vanilloid core. The reduced or absent activity of the **oVan**-, **iVan**-, and **eVan**-derived compounds **2**–**4** implies that appropriate electronic and steric environments around the aldehyde/imine region may be required to maintain productive interactions with bacterial targets.

Within the *O*-alkylated series (**5**–**12**), activity remained generally moderate and highly dependent on both the vanilloid core and the nature of the alkyl substituent. Several derivatives derived from **Van** (**5V**, **8V**, and **9V**) displayed MIC values in the 32–64 µg/mL range, showing a modest improvement (at least two-fold lower MICs) over the parent **Van**. Derivatives bearing an alkyl chain with a terminal chlorine atom, **8V** and **9V,** showed moderate and strain-dependent activity. In particular, **8V** displayed its lowest MIC value against strain F1, whereas **9V** showed its highest activity against NCTC 11637 and strain 23.

Conversely, the **oVan**-based *O*-alkylated derivatives (**6oV** and **11oV**) showed variable outcomes, with propargylated compound **6oV** exhibiting the best performance in the series (MIC 32 µg/mL), consistent with the generally more favorable antibacterial profile of the **oVan** core. In addition, ethyl acetate **11oV** showed reduced efficacy (MIC ≥ 64–128 µg/mL), indicating that not all alkyl chains synergize with the *ortho*-hydroxy arrangement. In contrast, **eVan**-based *O*-alkyl derivatives (propargylated **7eV** and ethyl acetate **12eV**) were largely inactive (MIC ≥ 128 µg/mL), and this trend seems to align with the poor intrinsic activity of **eVan**. Overall, observing the data in Table 1, we could assume that *O*-alkylation could only modestly enhance antibacterial activity, but its impact is highly scaffold-dependent, with **Van** derivatives showing an improvement, **oVan** derivatives retaining activity in the presence of a specific substitution pattern, and the tested monomeric **eVan** derivatives **7eV** and **12eV** remaining inactive regardless of alkylation. 

*O*-alkylated dimers **13**–**15** generally exhibited improved antibacterial activity compared to monomeric **Van** derivatives, with **Van**-based **13V** showing a consistent moderate activity across all strains (MIC/MBC = 64/64 µg/mL), **oVan** dimer **14oV** being the most active compound in this series (MIC/MBC = 16–32 µg/mL, depending on the strain), and **eVan** dimer **15eV** exhibiting moderate activity (MIC = 64 µg/mL), superior to the monomeric **eVan** (MIC ≥ 128 µg/mL). These results suggest that the effect of dimerization is dependent on the vanilloid core, with **14oV** showing the highest activity within this series. As shown in Table 1, the introduction of aromatic *O*-substituents in compounds **16**–**29** generally enhanced the antibacterial potency compared to **Van** and its analogues, with several derivatives displaying MIC values in the 4–16 µg/mL range, as also previously demonstrated for carvacrol- and thymol-derivatives, but not for eugenol-based compounds [44]. Among them, the oxadiazole-containing vanilloid **16V**, incorporating a phenyl-oxadiazole tail, emerged as the most potent anti-*H. pylori* agent, with MIC and MBC values of 4 µg/mL across all tested strains.

In several cases, **Van**-based derivatives consistently outperformed the corresponding **oVan** and **eVan** analogues (e.g., **16V** vs. **17eV** and **23V** vs. **24**–**26**). Generally, comparison of the 3- and 4-nitro, 3-trifluoromethyl, and 4-bromo substitutions in the *O*-benzylated derivatives series suggested a possible interplay between benzyl-ring substitution and the **Van** core. In particular, nitro-substituted compounds (**19**–**22**) showed strong core dependence: while the **oVan**-based derivative **20oV**, endowed with a 3-nitrobenzyl tail, is among the most potent compounds (MIC 8–16 µg/mL), the corresponding **Van** isomer **19V** is nearly inactive, and the **eVan** analogue **21eV** possesses only modest activity. Otherwise, **Van**-based 4-nitrobenzyl derivative **22V** exhibited intermediate potency. Replacing the 3-nitro group with another electron-withdrawing function, such as 3-trifluoromethyl (**23**–**26**), provided equipotent or more potent compounds, with the **Van**-based **23V** being the most active (MIC 8–16 µg/mL), followed by **25iV** and **24oV** analogues, and a clear loss of activity in the **eVan**-based derivative **26eV**. Contrariwise, the 4-bromobenzyl series (**27**–**29**) displayed intermediate activity, with **eVan**-based **29eV** showing slightly greater activity than the corresponding **oVan** analogue **28oV** and matching or exceeding the **Van** derivative **27V**. More complex compounds, such as *O*-naphthylmethylated **30iV** and **31eV**, showed moderate activity (MIC 16–32 µg/mL), with **31eV** being more potent than the analogue **30iV**.

Observing data reported in Table 1, the *O*-benzylated dimers **32–37**, derived from either 1,2-benzenedimethanol (**32**–**34**) or 1,4-benzenedimethanol (**35**–**37**), displayed moderate and fairly uniform antibacterial activity (MIC 64–128 µg/mL). Within both dimer series, **eVan**-based dimers **34eV** and **37eV** consistently showed the best activity (MIC = 64 µg/mL), whereas **Van**- (**32V** and **35V**) and **oVan**- (**33oV** and **36oV**) based dimers were generally less potent (MIC 64–128 µg/mL). As regards the ester series (**38**–**41** and **55oV**), the **oVan**-adamantane ester **55oV** was the most active compound of this subset, with MIC values of 8–16 µg/mL; **Van**-adamantane ester **38V** and **oVan**-coumarin ester **41oV** showed moderate activity (MIC 32–64 µg/mL), whereas the **eVan**-adamantane ester **39eV** was largely inactive (MIC ≥ 128 µg/mL). **Van**-coumarin ester **40V** displayed intermediate potency (MIC 64–128 µg/mL).

Finally, the thiosemicarbazone of *O*-3-nitrobenzylated **oVan**, **42oV,** was found to exhibit promising anti-*H. pylori* potency, with MIC values of 4–16 µg/mL. This compound and its derivatives were also designed to mask the aldehyde moiety, which can be metabolically unstable and reactive. In contrast, focusing attention on benzylidene hydrazinylthiazoles **43**–**46oV**, we could notice that most of them were largely inactive (MIC ≥ 128 µg/mL), despite variation in the phenyl substituent: **44oV** (unsubstituted), **43oV** (4-nitro), and **45oV** (3-nitro). Notably, derivative **46oV**, bearing a distal phenol ring (4-hydroxy), displayed moderate activity (MIC = 16–64 µg/mL), thus indicating that thiazole ring formation requires specific substituents to restore or confer antibacterial activity.

### 2.3. Effect on Cell Viability

**Figure 4:**
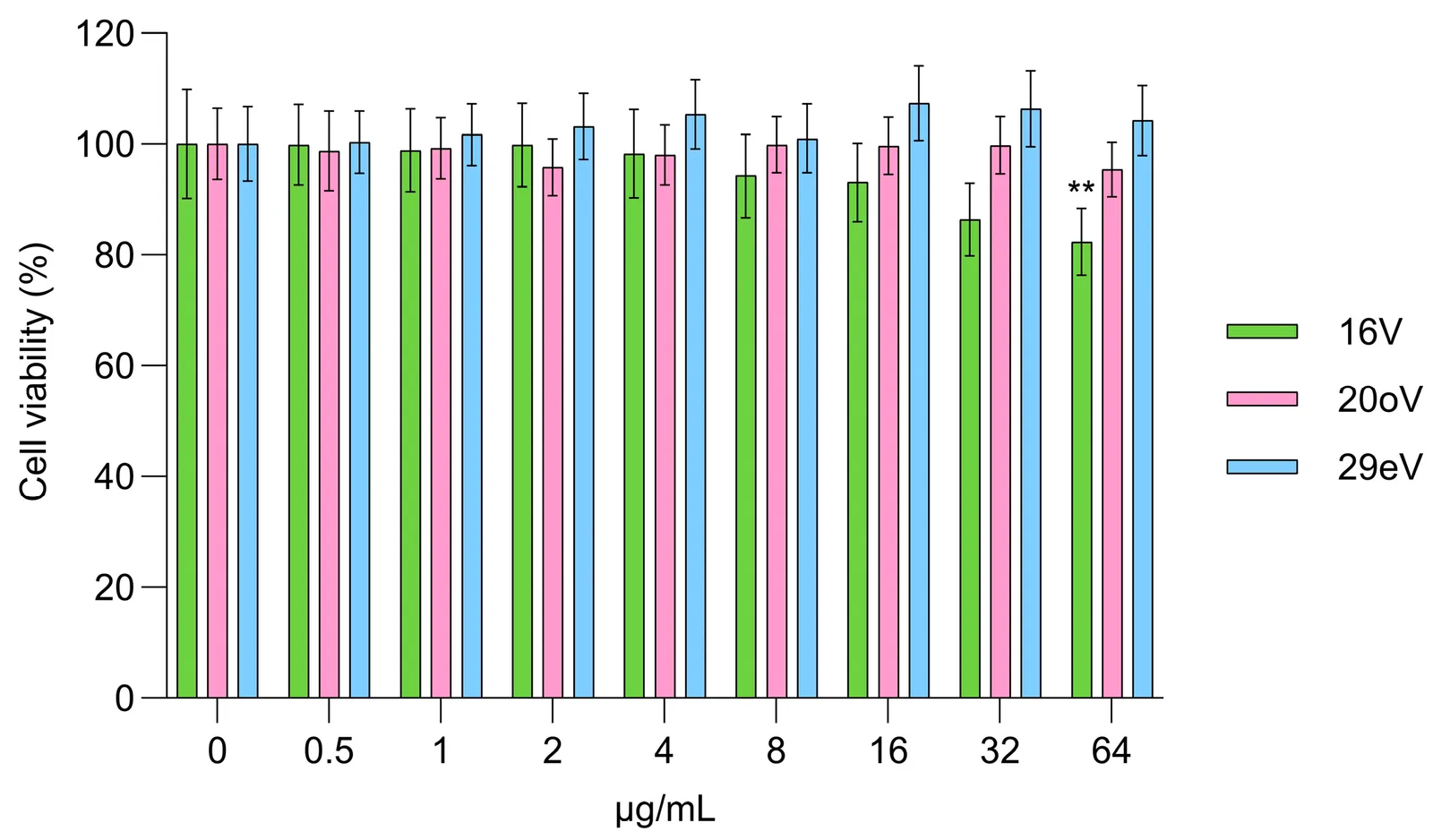
Cell viability of GES-1 cells exposed to compounds **16V**, **20oV**, and **29eV** for 24 h. Data (mean ± SD, n = 3) are expressed as percentage (%) compared to untreated cells. Statistical significance was assessed by ordinary two-way ANOVA followed by Dunnett’s multiple-comparison test. **: *p* = 0.0086.

We tested the effects of compounds **16V**, **20oV,** and **29eV** on GES-1 cell viability because they were selected as representative most potent active compounds with three distinct structural series. Treatment with compound **16V** at 64 µg/mL resulted in a modest but statistically significant reduction in cell viability to 82%, a concentration 16-fold higher than its MIC and MBC values of 4 µg/mL (Figure 4).

None of the tested compounds reduced cell viability by 50% within the investigated concentration range; therefore, their IC_50_ values were estimated to be higher than the maximum tested concentration of 64 µg/mL (Figure 4). Accordingly, the selectivity index (SI) was calculated for each compound and reported in Table 2.

Overall, the selected compounds showed limited effects on GES-1 cell viability at concentrations several-fold higher than their antibacterial MIC values.

### 2.4. Time-Kill Curve and Antibacterial Spectrum

**Figure 5:**
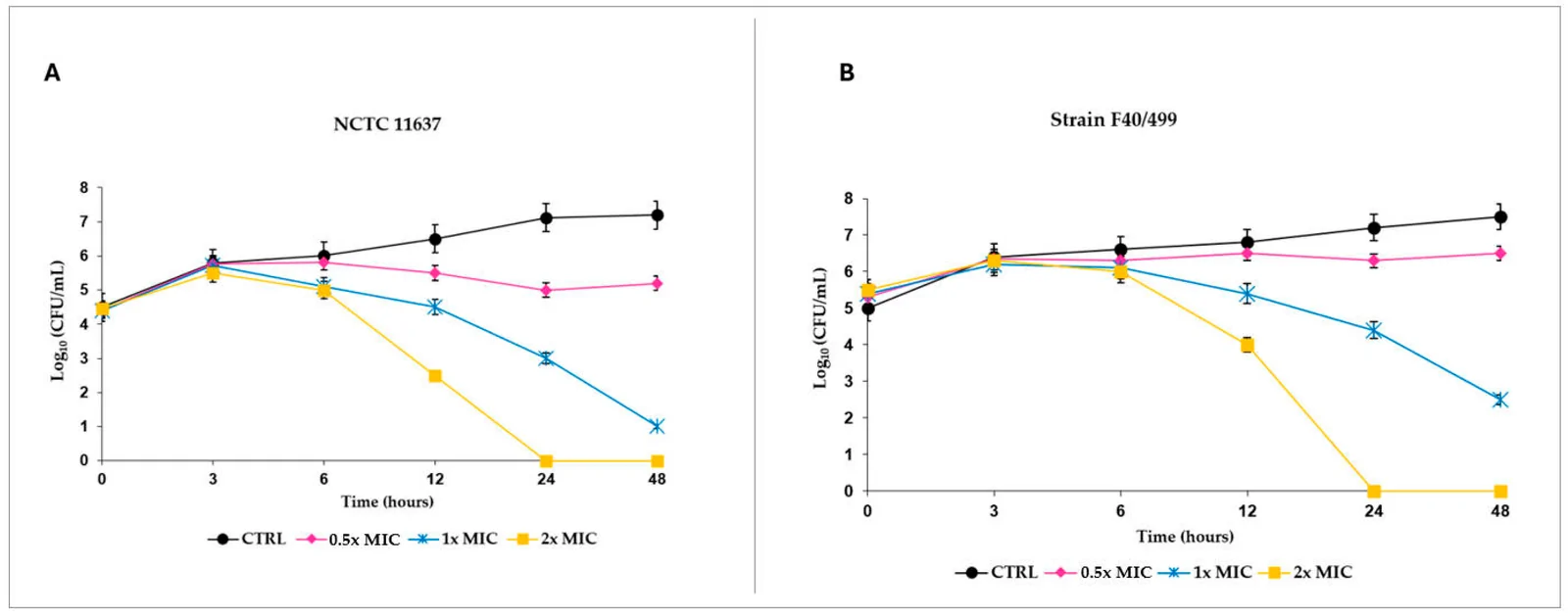
Time-kill kinetics of compound **16V** against *H. pylori* NCTC 11637 (**A**) and the clinical strain F40/499 (**B**). Antibacterial activity was evaluated in MegaCell RPMI medium supplemented with 3% FCS in the presence or absence of compound **16V** at 0.5×, 1×, and 2× MIC at different time points. MIC of compound **16V** against both NCTC 11637 and F40/499 was 4 µg/mL. Data are expressed as mean log_10_ CFU/mL ± SD from three different experiments performed in triplicate.

A kinetic study was performed against *H. pylori* NCTC 11637 and the MTZ/CLR-resistant clinical isolate F40/499 (Figure 5). The antibacterial effect of compound **16V** increased with increasing concentrations, ranging from ½ to 2× MIC. Treatment for 12 h with 2× MIC caused a 4 log_10_ CFU and ~3 log_10_ CFU decrease relative to the initial inoculum, for *H. pylori* NCTC 11637 and F40/499 clinical strain, respectively. Total killing was achieved after 24 h.

Marked reductions in viable counts were also observed for both strains after 24 h at 1× MIC; after 48 h of incubation, a reduction of 6 log_10_ CFU was observed in *H. pylori* NCTC 11637; ~5 log_10_ CFU decrease was observed for strain F40/499. Treatment with 0.5× MIC showed only a marginal killing effect on both strains after 24 h of incubation.

To verify the specificity of compounds **16V**, **20oV**, and **29eV** against *H. pylori*, other representative Gram-negative and Gram-positive bacterial strains were also investigated. As shown in Table 3, all compounds were found to be inactive against the tested bacteria with respect to ciprofloxacin (CPX) as a reference drug.

### 2.5. Chemometric Analyses

#### 2.5.1. Hierarchical Cluster Analysis (HCA) and Clustering by Artificial Neural Networks (CANN)

**Figure 6:**
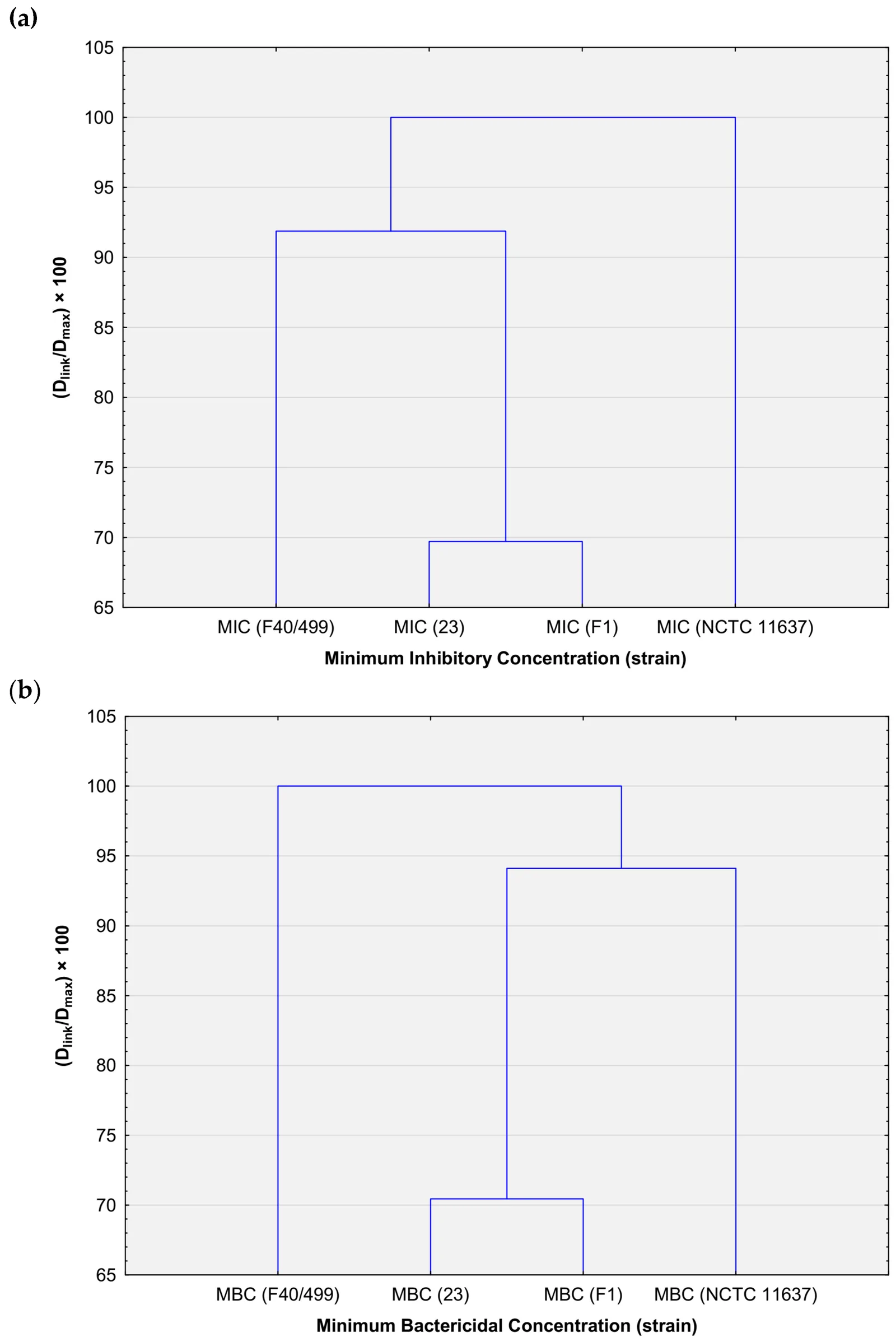
Dendrograms of HCA based on (**a**) MIC and (**b**) MBC values of the synthesized vanilloids tested on four different *H. pylori* strains.

Aimed at systematically evaluating the anti-*H. pylori* potential of the synthesized vanilloids and given the large number of data/structures generated in this paper, a Hierarchical Cluster Analysis (HCA) was performed, based on individual MIC and MBC data collected across the four clinically relevant strains tested. Briefly, through a multivariate approach, the compounds underwent clustering according to the similarity of their activity profiles, and groups sharing comparable antibacterial/bactericidal potency were identified. Specifically, HCA is able not only to highlight structural motifs and isomeric variations associated with enhanced anti-*H. pylori* activity but can also reveal potential strain-specific effects, providing a deep understanding of SARs within the vanilloids library. Thus, the resulting clusters could serve as a rational framework to prioritize compounds for further investigation, guiding subsequent mechanistic studies and library optimization efforts. The results of HCA are presented through dendrograms in Figure 6.

In the HCA dendrograms, the x-axis displays the four *H. pylori* strains, while the y-axis represents the relative distance or dissimilarity between them, with higher vertical lines indicating greater differences in the MIC or MBC values across strains. Conversely, strains that are shown closer together in the dendrogram exhibit more similar antibacterial responses to the synthesized compounds.

Observing Figure 6, in both dendrograms, strains F1 and 23 cluster at the lowest level, indicating that the compounds display highly similar MIC and MBC profiles against these two strains. This suggests comparable susceptibility patterns. In contrast, the NCTC 11637 strain is located at the highest branching point in the MIC-related dendrogram (Figure 6a), demonstrating substantial dissimilarity from the other strains and indicating that the MIC values for this strain differ markedly from those of the others. The F40/499 strain occupies an intermediate position between the two clusters in this MIC dendrogram (Figure 6a). For the MBC-based dendrogram (Figure 6b), the pattern is reversed: F40/499 emerges as the most distant strain, whereas NCTC 11637 forms an intermediate cluster relative to the others. Overall, the HCA results for both MIC and MBC data consistently highlight the strong similarity in antibacterial responses between strains F1 and 23, which cluster tightly together. Greater dissimilarity is observed for the remaining strains, with variations in their relative positions depending on whether MIC or MBC values are considered.

**Figure 7:**
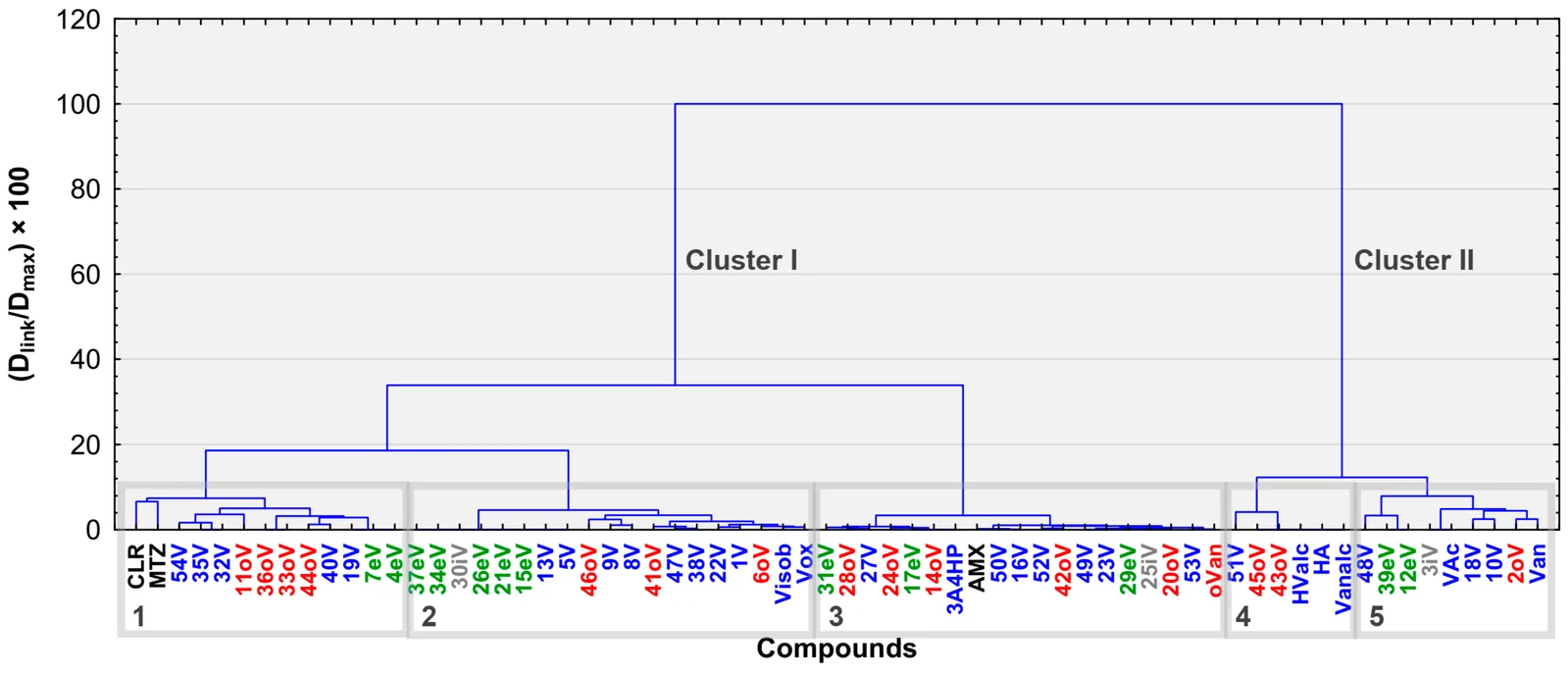
HCA dendrogram of the following tested vanilloids: the parent compounds [reported as **Van** (blue): vanillin; **oVan**: *o*-vanillin (red)], commercial analogues [reported as **Visob**: vanillin isobutyrate; **Vox**: vanillin oxime; **HValc**: homovanillin alcohol; **HA**: homovanillic acid; **Vanalc**: vanillin alcohol; **Vac**: vanillin acetate; **3A4HP**: 3-allyl-4-hydroxyacetophenone], synthetic derivatives [reported as the number of the compound followed by a code and color corresponding to the vanilloid core, as V: vanillin (blue); oV: *o*-vanillin (red); iV: *iso*-vanillin (gray); eV: ethylvanillin (green)], and reference antibiotics [AMX; CLR; MTZ (black)].

Figure 7 shows the HCA dendrogram obtained for the parent compounds, their commercial analogues and synthetic derivatives, and reference antibiotics (AMX, CLR, MTZ), based on their MIC and MBC values across the four *H. pylori* strains.

Two major clusters are clearly distinguishable: Cluster I, comprising sub-clusters 1–3, and Cluster II, comprising sub-clusters 4 and 5. Overall, Cluster I groups compounds endowed with stronger anti-*H. pylori* activity, either across all the tested strains or selectively toward at least one strain. Conversely, Cluster II contains compounds with the weakest antibacterial activity, showing consistently higher MIC and MBC values against all tested strains. Within Cluster I, sub-cluster 3 includes the most active compounds in the whole library, together with AMX as a reference antibiotic that exhibited the highest antibacterial potency. Sub-cluster 2, positioned closer to sub-cluster 3, contains compounds with significant activity, although slightly weaker than those in sub-cluster 3. Sub-cluster 1 comprises the least active compounds along with the reference antibiotics CLR and MTZ, both showing reduced activity towards at least one strain, specifically MTZ against NCTC 11637 and CLR against the F40/499 strain.

All three reference antibiotics fall within cluster I, reflecting that each of them demonstrates substantial activity against at least one *H. pylori* strain. Notably, the dendrogram in Figure 7 did not reveal strict clustering according to the parent vanilloid core or the main chemical series, indicating that the anti-*H. pylori* activity does not directly correlate with specific structural features within this series.

As part of the exploratory data analysis, we also applied artificial neural network-based clustering (CANN) to complement the insights obtained from hierarchical clustering. While the dendrograms in Figure 6 provide an interpretable, hierarchical organization of similarities among the compounds, the CANN approach, implemented as a self-organizing feature map (SOFM), was employed to uncover more complex, deeper, non-linear patterns that may not be detectable by means of distance-based methods alone. To perform this analysis, along with the compounds reported in Table 1, we also considered the vanillin-based azo derivatives previously published by us (Table S1) [7]. The resulting SOFM exhibited a training error of 0.119, a test error of 0.145, and a validation error of 0.287, thereby indicating a satisfactory learning performance and generalization capability. The trained network consisted of six neurons, arranged and labeled according to their grid coordinates: (1, 1), (1, 2), (1, 3), (2, 1), (2, 2), and (2, 3). Each neuron represents a cluster centroid, and compounds are assigned to the neuron whose weight vector most closely matches their MIC/MBC activity profile. The distribution of compounds across these six neurons is presented in Table 4.

Table 4 illustrates how SOFM grouped compounds based on multi-dimensional similarity patterns. While hierarchical clustering organizes compounds along a tree-like structure, the CANN-based map projects them onto a two-dimensional topology, allowing the recognition of subtle, non-linear relationships in their antibacterial behavior. By comparing the compounds distribution in clusters obtained by HCA with their assignment to SOFM neurons, it is evident that the two clustering approaches produce highly consistent groupings, with only a few exceptions.

Most compounds belonging to Cluster I (sub-clusters 1, 2, and 3) were mapped to five neurons: (1, 1), (1, 2), (2, 1), (2, 2), and (2, 3), and were characterized by low activation values, indicating short Euclidean distances between each compound and its corresponding neuron centroid. Compounds from Cluster II (sub-clusters 4 and 5) were predominantly assigned to a single neuron (1, 3), reflecting their shared profile of weak antibacterial activity across the tested strains. The only exception is compound **48V**, which is positioned in neuron (1, 2). Overall, the distribution of compounds across neurons closely mirrors their allocation to sub-clusters 1–5 with slight deviations. In particular, compounds in sub-cluster 3 are grouped within neuron (1, 1); compounds in sub-cluster 1 are predominantly located in neuron (1, 2); compounds in sub-cluster 2 are mainly in neuron (2, 1); MTZ appears to be placed alone in neuron (2, 2), consistent with its distinctive activity profile. Moreover, compounds from the external test set were assigned to the same clusters as in HCA, further supporting the robustness of the SOFM model. Consistent with the HCA results, the CANN analysis confirmed the absence of strict structural grouping, indicating that antibacterial activity within this series does not correlate directly with specific structural features.

#### 2.5.2. Principal Component Analysis

**Figure 8:**
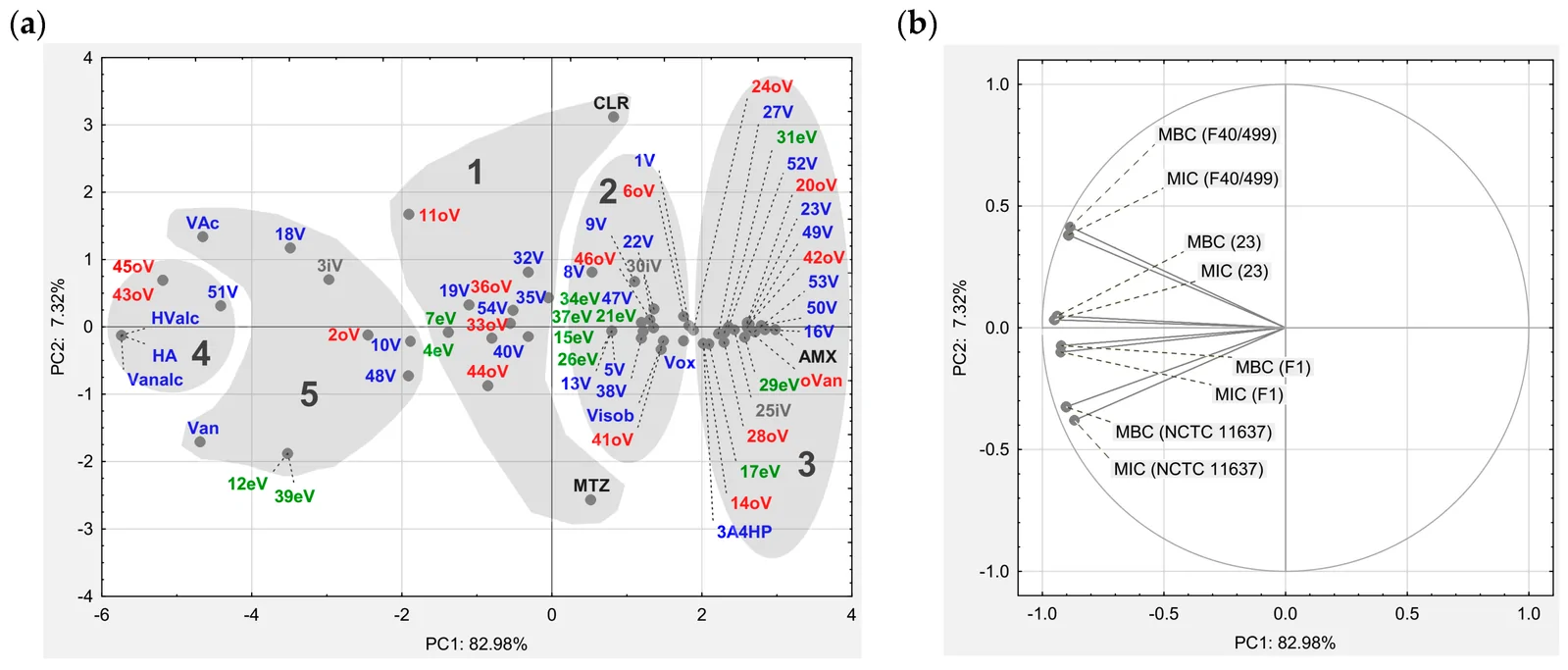
Distribution of (**a**) the compounds in the score plot and (**b**) activity data in the loadings plot. Parent compounds are reported as **Van** (blue): vanillin; **oVan**: o-vanillin (red); commercial analogues are reported as **Visob**: vanillin isobutyrate; **Vox**: vanillin oxime; **HValc**: homovanillin alcohol; **HA**: homovanillic acid; **Vanalc**: vanillin alcohol; **Vac**: vanillin acetate; **3A4HP**: 3-allyl-4-hydroxyacetophenone; synthetic derivatives are reported as the number of the compound followed by a code and color corresponding to the vanilloid core, as V: vanillin (blue); oV: o-vanillin (red); iV: iso-vanillin (gray); eV: ethylvanillin (green); reference antibiotics AMX; CLR; MTZ are in black.

Principal Component Analysis (PCA) was performed to obtain an overall view of how the compounds are distributed within the multidimensional space defined by their antibacterial activities against *H. pylori* strains. The score and loading plots are presented in Figure 8.

In the score plot (Figure 8a), a clear trend can be observed along the PC1 axis: the most active compounds, together with AMX, are located at the positive end of PC1, whereas compounds with low activity cluster toward its negative end. The grouping pattern is partially consistent with the clusters identified by HCA of the compounds, highlighted by gray regions on the score plot. However, PCA additionally reveals which variable (activity towards specific strain) primarily drives these separations, as illustrated in the loading plot (Figure 8b). Specifically, MIC and MBC values against strains F40/499 and NCTC 11637 seem to exhibit the strongest influence on compound distribution along the PC2 axis, while the influence of all the variables is quite the same along the PC1 axis. Along the PC2 axis, a pronounced separation is observed for CLR and MTZ, which are at opposite ends of the axis. Specifically, CLR showed high activity against reference strain NCTC 11637 but markedly lower activity against clinical strain F40/499, whereas MTZ displayed the opposite pattern. Conversely, AMX lies near the line defined by y = 0, consistent with its high antibacterial potency across all the tested strains.

### 2.6. In Silico ADME Predictions

All the synthesized compounds were analyzed using SwissADME, a free online tool for the in silico evaluation of ADME properties [63,64,65]. Most of the designed derivatives were not identified as potential Pan-Assay Interference Compounds (PAINS). However, three thiosemicarbazone compounds **1V**, **2oV**, and **4eV** showed PAINS alerts, associated with the presence of a phenolic moiety (Supplementary Material). Interestingly, the most active compound **16V** exhibited favourable drug-like properties, fully complying with Lipinski’s rules [66,67]. Moreover, **16V** showed predicted high gastrointestinal absorption; a key feature to be considered for a potential efficacy against *H. pylori*, which colonizes the gastric mucosa [68,69].

### 2.7. Molecular Docking

Molecular docking was performed as an exploratory analysis to investigate the possible accommodation of the synthesized compounds within the catalytic site of *H. pylori* urease. This enzyme was selected as a putative molecular target because it plays a crucial role in bacterial survival and colonization of the gastric environment. By catalyzing the hydrolysis of urea into ammonia and carbon dioxide, urease contributes to the local neutralization of gastric acidity, thereby allowing *H. pylori* to persist within the gastric mucosa. Accordingly, interference with urease activity represents a potentially relevant strategy for limiting bacterial survival.

**Figure 9:**
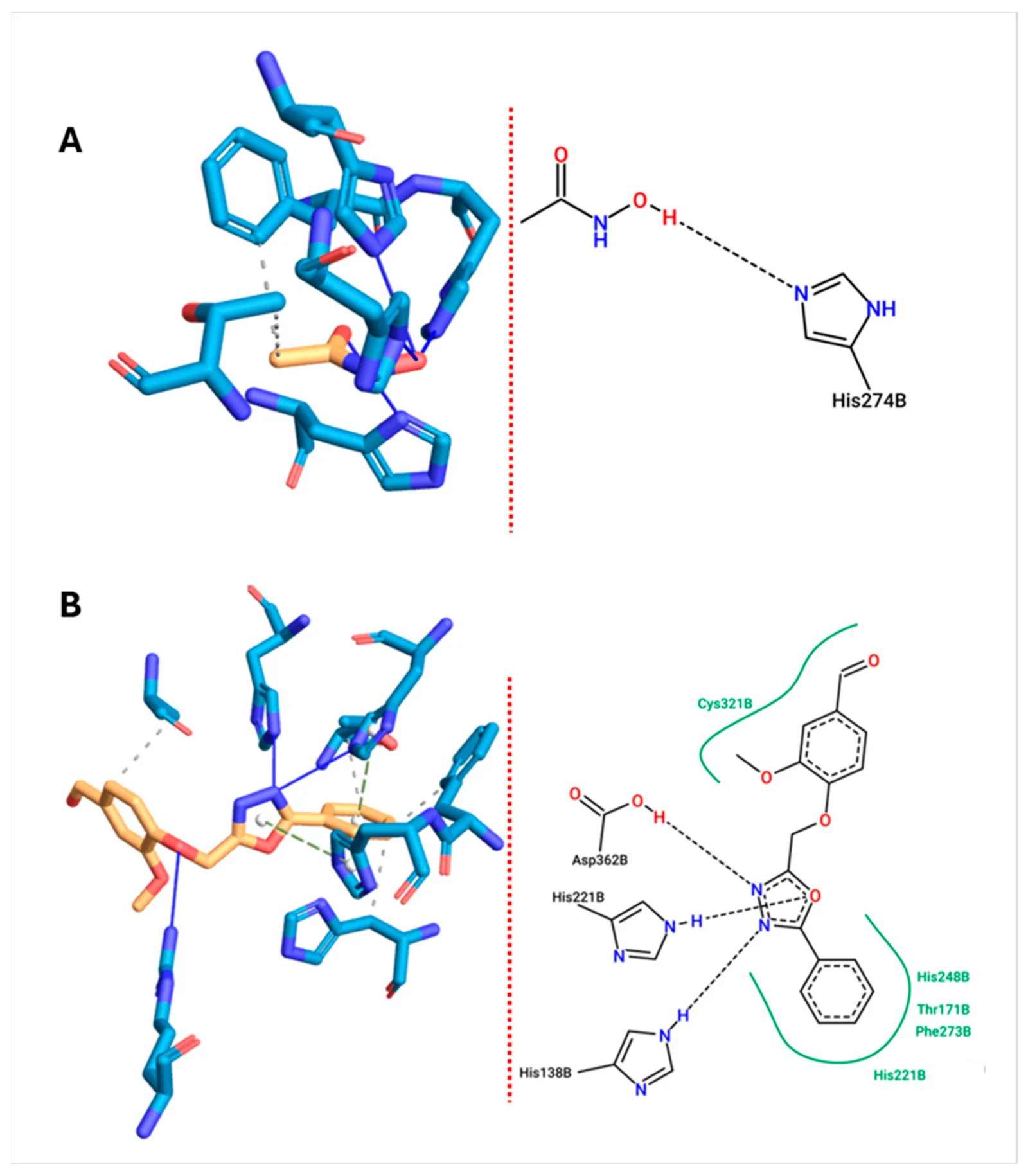
Predicted binding modes of acetohydroxamic acid and compound **16V** within the catalytic site of *H. pylori* urease (PDB ID: 1E9Y). 3D representations are shown on the left and corresponding 2D interaction diagrams on the right. (**A**) Redocked pose of the co-crystallized acetohydroxamic acid. (**B**) Top-ranked docking pose of compound **16V**, showing predicted polar contacts with His138B, His221B, and Asp362B, and hydrophobic interactions with residues lining the binding pocket. Dashed lines indicate polar interactions, while green arcs indicate hydrophobic contacts.

For the docking study, the X-ray crystal structure of *H. pylori* urease in complex with acetohydroxamic acid was selected (PDB ID: 1E9Y) [70]. The docking protocol was first evaluated by redocking the co-crystallized acetohydroxamic acid into its native binding site, yielding an RMSD value of 1.207 Å. The redocked pose reproduced the experimentally observed orientation of the reference ligand within the binuclear nickel-containing catalytic site and showed a hydrogen-bond interaction with His274B, as illustrated in Figure 9A.

Docking simulations were subsequently performed for the entire series of vanilloid derivatives. Among the investigated compounds, **16V**, which was also the most active derivative in the antibacterial assays, showed the most favorable docking score (−8.2 kcal/mol; Table S2). Its predicted binding pose within the urease catalytic pocket is shown in Figure 9B. The oxadiazole moiety of **16V** was predicted to establish polar interactions with residues His221B, Asp362B, and His138B. In particular, the oxadiazole oxygen was predicted to interact with His221B, whereas the two endocyclic nitrogen atoms were oriented toward Asp362B and His138B, respectively.

The docking scores obtained for the complete compound library and for the co-crystallized reference inhibitor acetohydroxamic acid are reported in Table S2. Although these results suggest that **16V** may be accommodated within the urease catalytic pocket, they should be considered preliminary and do not establish urease inhibition as the mechanism responsible for its antibacterial activity. Direct enzymatic studies will therefore be required to confirm target engagement.

## 3. Materials and Methods

### 3.1. Chemical Synthesis of Vanilloids

#### General Chemistry

All reagents and solvents were obtained from Merck (Milan, Italy). Purification of the synthesized compounds was carried out by flash column chromatography using silica gel with a particle size of 230–400 mesh. The progress of the reactions was monitored by thin-layer chromatography (TLC) on aluminum-supported silica gel plates coated with silica gel 60 F_254_. Spots were detected by UV irradiation at 254 nm. NMR spectra were acquired using 300/400/600 MHz NMR spectrometers (Varian-Mercury 300 from Agilent Technologies, Santa Clara, CA, USA, Bruker Avance 400 from Bruker Corporation, Karlsruhe, Germany, and Jeol JNM-ECZ 600 R from JEOL Ltd., Tokyo, Japan). Chemical shifts are given as *δ* values in parts per million (ppm), using the residual solvent peaks of CDCl_3_ and DMSO-*d*_6_ as internal references. Data are reported as chemical shift, multiplicity, integration, and coupling constant (*J*). The abbreviations are: s, singlet; d, doublet; t, triplet; q, quartet; qi, quintet; m, multiplet or overlapping multiplets; br, broad signal; and ap, apparent signal. Microwave-assisted syntheses were carried out using a Biotage^®^ Initiator+ reactor (Biotage Sweden AB, Uppsala, Sweden) equipped with vials suitable for single-mode irradiation. Melting points were determined with a Stuart^®^ SMP1 apparatus (Fisher Scientific Italia, Segrate, Milan, Italy) and are reported in °C without correction. Elemental composition was assessed for carbon, hydrogen, and nitrogen using a Perkin-Elmer 240B microanalyzer (PerkinElmer Italia SpA, Milan, Italy). For all tested compounds, the experimental values deviated by no more than ±0.4% from the calculated ones. Detailed synthetic procedures and characterization data for all compounds are provided in the Supplementary Materials.

### 3.2. Bacterial Strains and Culture Conditions

*H. pylori* strains were maintained at −80 °C in Wilkins Chalgren Broth (Thermo Fisher Scientific, Segrate, Milan, Italy) with 10% (*v*/*v*) horse serum (Seromed) and 20% (*v*/*v*) glycerol (Merck, Milan, Italy) until required for experiments. Before being used, the strains were subcultured twice on Columbia agar base (Thermo Fisher Scientific, Segrate, Milan, Italy) supplemented with 10% horse serum and 0.25% Bacto yeast extract (Thermo Fisher Scientific, Segrate, Milan, Italy). Plates were incubated for 72 h at 37 °C in microaerophily (85% N_2_, 10% CO_2_, 5% O_2_).

Gram-negative strains *(Escherichia coli* ATCC 35218, *Pseudomonas aeruginosa* ATCC 19660) and Gram-positive strains (*Staphylococcus aureus* clinical strain ST25, *Staphylococcus aureus* ATCC 29213) were selected for this study. Gram-negative strains were cultured on MacConkey Agar (Thermo Fisher Scientific, Segrate, Milan, Italy) for 18 h at 37 °C, except for *P. aeruginosa,* which was cultivated on cetrimide agar. *S. aureus* strains were cultivated on mannitol salt agar (MSA) (Thermo Fisher Scientific, Segrate, Milan, Italy) for 24 h at 37 °C.

### 3.3. Minimum Inhibitory Concentration (MIC) and Minimum Bactericidal Concentration (MBC) Determination

MIC and MBC values on *H. pylori* NCTC 11637 and three clinical isolates were determined by a modified broth microdilution assay, as previously described [71]. Each compound was dissolved in DMSO (10 mg/mL), and two-fold serial dilutions were prepared in 96-well microtiter panels containing 100 µL of MegaCell^TM^ RPMI-1640 Medium (Merck, Milan, Italy) with 3% fetal calf serum (FCS) (HyClone, Logan, UT, USA). All compounds were tested in the range of 128–0.5 μg/mL. Each well was inoculated with 100 µL of *H. pylori* suspension at a final concentration of approximately 5 × 10^5^ CFU/well. The plates were incubated at 37 °C in microaerophily. After 72 h, the plates were examined visually, and MIC was defined as the lowest concentration of compounds inhibiting visible bacterial growth. Medium-only and DMSO vehicle controls were also included. The highest final DMSO concentration was approximately 1.25% and did not inhibit the growth of any of the tested strains.

Then, aliquots (10 μL) of all suspensions without visible growth were plated on Columbia agar base supplemented with 10% horse serum, 0.25% bacto yeast extract, and incubated at 37 °C for 72 h in microaerophily. The MBC was defined as the lowest concentration of the compound that killed ≥ 99.9% of the initial inoculum. Antibiotic susceptibility was assessed according to EUCAST guidelines, and MICs and MBCs are expressed in μg/mL as the average from experiments performed at least in triplicate. Antibiotic susceptibility of the strains: MTZ^R^ = metronidazole resistant (MIC > 8 μg/mL); MTZ^S^ = metronidazole susceptible (MIC ≤ 8 μg/mL); CLR^R^ = clarithromycin resistant (MIC > 0.25 μg/mL); CLR^S^ = clarithromycin susceptible (MIC ≤ 0.25 μg/mL); AMX^S^ = amoxicillin susceptible (MIC ≤ 0.125 μg/mL).

MICs for *E. coli* ATCC 35218, *P. aeruginosa* ATCC 19660, *S. aureus* ATCC 29213, and *S. aureus* clinical strain ST25 were performed using the microdilution method in cation-adjusted Mueller-Hinton broth (CAMHB) [72] on a 96-well microtiter plate containing 1 × 10^5^ CFU/well. The plate was incubated at 37 °C for 24 h. MIC with ciprofloxacin (CPX) was performed as a quality control. The isolated strains have been previously obtained during routine activity of our hospital laboratory. The clinical isolates had been previously obtained during routine diagnostic activity and were fully anonymized before their use in this study. No additional human samples or identifiable patient data were collected. Therefore, according to institutional regulations, additional ethical approval and informed consent were not required. The strains were identified based on the colony appearance, Gram staining, and positive reactions in biochemical tests.

### 3.4. Cell Culture and Treatments

Human gastric epithelial SV40-immortalized non-tumorigenic GES-1 cells (kindly provided by Prof. Dawid Kidane, Department of Pharmacology and Toxicology, University of Texas, Austin, TX, USA) were cultured in RPMI medium (HyClone, Logan, UT, USA) supplemented with 10% decomplemented fetal calf serum (FCS) (HyClone, Logan, UT, USA), 1% levoglutamine (Merck, Milan, Italy), 1% penicillin/streptomycin (HyClone, Logan, UT, USA), and 10 mM Hepes (Merck, Milan, Italy). Cells were maintained at 37 °C in a humidified atmosphere with 5% CO_2_. Cells were seeded into 96-well plates at 10^5^ cells/mL and allowed to attach overnight. Cells were then treated for 24 h with compounds **16V**, **20oV**, and **29eV** at concentrations ranging from 0.5 to 64 µg/mL. Control cells with only medium and control cells with medium containing the corresponding DMSO concentration were also included in each experiment. The highest final DMSO concentration was approximately 0.6% and did not affect GES-1 cell viability.

At the end of treatment, cell viability was assessed by MTT assay as previously described [73,74]. Data were statistically analyzed by GraphPad Prism 10.6.1 (San Diego, CA, USA) using the ordinary two-way analysis of variance (ANOVA) followed by Dunnett’s multiple comparison tests, with a single pooled variance. Results were expressed as mean ± standard deviation. Each *p*-value was adjusted to account for multiple comparisons, with a 0.05 alpha threshold and 95% confidence interval.

### 3.5. Killing Kinetics

Time–kill kinetic curves of *H. pylori* NCTC 11637 and clinical strain F40/499 were obtained using compound **16V** at 0.5×, 1×, and 2× the MIC, at different time points (0, 3, 6, 12, 24 h, and 48 h of incubation). The number of CFUs was assessed by serial dilution. The results were expressed as viable count (log_10_ CFU/mL).

### 3.6. Chemometric Analyses

The chemometric analysis was performed using Statistica v.12 software. For MIC/MBC values reported as “>128”, the numerical value of 256 was assigned in all chemometric analyses to ensure consistent numerical treatment across the data set [75]. In HCA, Ward’s algorithm was used as an amalgamation (linkage) rule, and Euclidean distances were calculated as a distance measure. The data on the *y*-axis were normalized by using the equation D_link_/D_max_ × 100, where D_link_ refers to the linkage distance at which two clusters are merged, and it actually stresses how dissimilar the clusters are at the merging point, while D_max_ means the largest dissimilarity at which any two clusters were joined. In this way, the linkage distances were expressed as a percentage of the maximum linkage distance.

CANN analysis was based on Kohonen training, which was used to reveal the clusters in the data set. The training was performed, and five compounds were initially omitted from the set and were used as an external test set (**Van**, **27V**, **44oV**, **25iV**, **34eV**). The external test set was formed by random selection of compounds to provide an unbiased evaluation of the model’s generalization ability without introducing any systematic preference. The training was further performed: 70% of the data was used in the training, 15% in the test, and 15% in the validation procedure. Topological height was set at 2, while the topological width was 3. Kohonen training was based on 5000 training cycles. The PCA was based on correlations, and the variances were calculated as *SS*/(*N* − 1), where *SS* refers to the sum of squares and *N* is the number of objects (samples). The PCA resulted in a model in which the first two PCs cover 90.30% of the total variability. PC1 covers 82.98% and PC2 7.31% of the total variance.

### 3.7. Molecular Docking Studies Against Urease

Selection and Preparation of the Target Protein: To elucidate the targeted interaction profiles of the synthesized compounds intended for the treatment of *Helicobacter pylori*, the three-dimensional structure of the target protein was retrieved from the Protein Data Bank (PDB ID: 1E9Y). Although the target structure is a complex macromolecule elucidated by XRD, Chain B was specifically isolated and selected for the docking simulations. This was done to most accurately simulate in vitro physiological conditions within an in silico environment and to precisely evaluate the structural conformation of the active site [76].

During the receptor preparation phase, water molecules, the co-crystallized ligand, and other non-essential heteroatoms were removed from the protein structure, whereas the two catalytic Ni^2+^ ions were retained. Missing hydrogen atoms were added, protonation states were adjusted to physiological pH, and energy minimization was performed using standard algorithms. The coordinates of the binding pocket (active site) were centered using the co-crystallized acetohydroxamic acid ligand (PDB ligand code: HAE) present in the 1E9Y crystal structure as a reference. The grid box parameters were optimized to dimensions of 20 × 20 × 20 Å to comprehensively encompass this specific interaction region.

Preparation of Ligands: The compounds designed and synthesized within the scope of this study were designated as test ligands. In addition, acetohydroxamic acid was included as a reference drug. The 2D structures of all molecules were sketched and converted into their 3D conformations. Subsequently, geometric optimization and ligand energy minimization were completed using the MMFF94 force field, rendering the molecules ready for docking simulations.

Docking Protocol Using MzDOCK: The in silico molecular docking simulations were executed using the MzDOCK 2.7 software [77]. The synthesized compounds and acetohydroxamic acid were docked into the designated 20 × 20 × 20 Å grid area on Chain B of the 1E9Y protein. The binding affinities (binding free energies) of each ligand were calculated utilizing the scoring functions of the software. Among the nine generated binding poses, one pose was selected as the reference structure, and all subsequent analyses were performed based on this pose. The poses exhibiting the lowest energy and the most stable conformations were selected. Furthermore, hydrogen bonds, hydrophobic interactions, van der Waals forces, and π-π interactions between the test compounds and the amino acid residues in the target protein’s active site were analyzed in detail (Tables S3 and S4). The obtained in silico data were utilized to predict and compare the potential in silico inhibitory activities of the synthesized compounds against a reference urease inhibitor.

## 4. Conclusions

Searching for new antibacterial compounds within small molecules can provide important advantages in terms of predicted metabolism, flexible structural design, and SAR assessment, thus tackling drug-resistant bacteria and offering a platform for further optimizations by means of iterative modular synthesis and data-driven approaches. The application of this concept to natural compounds inspired researchers and accelerated the development of novel agents. In a similar vein to our previous research, we started from a basic natural scaffold (**Van** and its derivatives) to obtain semisynthetic compounds against *H. pylori*. Compounds **16V**, **20oV,** and **29eV** appeared to be the most potent (MIC_90_/MBC_90_ values of 4, 8, and 16 µg/mL, respectively). The most active compound in vitro was **16V**, which also showed a favorable binding score in molecular docking studies against urease. The large library presented here clearly demonstrated this successful MedChem strategy and provided new hit compounds to develop, especially for their narrow antibacterial spectrum against the additional species tested and good cell viability against a human cell line. These results also confirmed the pivotal role of naturally occurring metabolites as an important material source of biologically active compounds. Lastly, additional studies on their mechanism of action, gastric stability, activity under acidic/gastric-relevant conditions, emergence of resistance, synergy with standard antibiotics, and in vivo efficacy would be needed.

## Figures and Tables

**Table 1:** Anti-*H. pylori* activity of parent compounds, commercially available vanilloids, synthetic derivatives **1**–**46** and **55oV**, and reference antibiotics against four strains. Synthetic derivatives are reported with an alphanumeric code, with the number of the compound followed by a code corresponding to the vanilloid core, as V: vanillin, oV: *o*-vanillin, iV: *iso*-vanillin, eV: ethylvanillin.

	MIC/MBC (µg/mL)				MIC_50_/MIC_90_	MBC_90_
	NCTC 11637	F1	23	F40/499	(µg/mL)	(µg/mL)
**commercially available vanilloids**						
*parent compound*						
**Vanillin** **(Van)**	>128/>128	>128/>128	>128/>128	128/128	128/>128	>128
*isomers*						
***o-*** **Vanillin** **(oVan)**	8/16	4/4	16/16	8/8	4/16	16
***iso*** **-Vanillin** **(iVan)**	>128/>128	>128/>128	>128/>128	128/128	>128/>128	>128
*analogues*						
**Ethylvanillin** **(eVan)**	128/128	128/>128	128/128	128/128	128/>128	>128
**Vanillin alcohol (Vanalc)**	>128/>128	>128/>128	>128/>128	>128/>128	>128/>128	>128
**Homovanillyl alcohol (HValc)**	>128/>128	>128/>128	>128/>128	>128/>128	>128/>128	>128
**Homovanillic acid (HA)**	>128/>128	>128/>128	>128/>128	>128/>128	>128/>128	>128
**Vanillin oxime** **(Vox)**	32/64	32/32	32/32	32/32	32/32	64
*Commercially related compounds*						
**Vanillin acetate** **(Vac)**	128/128	>128/>128	>128/>128	>128/>128	>128/>128	>128
**Vanillin isobutyrate (Visob)**	64/64	32/32	32/32	32/64	32/64	64
**3′-allyl-4′-hydroxyacetophenone** **(3A4HP)**	32/32	32/32	32/32	16/16	32/32	32
*Synthetic vanilloids*						
**1V**	16/32	32/64	32/32	32/32	32/64	64
**2oV**	128/128	128/>128	128/>128	128/128	128/>128	>128
**3iV**	128/128	128/>128	128/>128	128/>128	128/>128	>128
**4eV**	128/128	128/128	128/128	128/128	128/128	128
**5V**	64/64	64/64	64/64	64/64	64/64	64
**6oV**	32/32	32/32	32/32	32/64	32/64	64
**7eV**	128/128	128/128	128/128	128/128	128/128	128
**8V**	64/64	32/32	64/64	128/128	64/128	128
**9V**	32/32	64/64	32/32	64/128	32/64	128
**10V**	128/128	128/>128	128/128	128/128	128/128	>128
**11oV**	128/128	64/64	128/128	>128/>128	64/128	>128
**12eV**	>128/>128	>128/>128	128/128	128/128	128/>128	>128
**13V**	64/64	64/64	64/64	64/64	64/64	64
**14oV**	32/32	32/32	32/32	16/16	32/32	32
**15eV**	64/64	64/64	64/64	64/64	64/64	64
**16V**	4/4	4/4	4/4	4/4	4/4	4
**17eV**	32/32	32/32	16/32	16/16	16/32	32
**18V**	128/128	>128/>128	128/128	>128/>128	128/>128	>128
**19V**	64/128	128/128	128/128	128/128	128/128	128
**20oV**	8/8	8/8	8/16	16/16	8/8	8
**21eV**	32/64	64/64	32/64	32/64	32/64	64
**22V**	32/32	64/64	32/32	64/64	32/64	64
**23V**	8/8	8/16	8/8	16/16	8/16	16
**24oV**	32/32	32/32	32/32	32/32	32/32	32
**25iV**	16/16	16/16	16/16	16/16	16/16	16
**26eV**	64/64	64/64	64/64	64/64	64/64	64
**27V**	16/32	16/16	32/32	16/16	16/32	32
**28oV**	32/32	16/16	16/16	16/16	16/16	32
**29eV**	16/16	16/16	8/8	8/8	8/16	16
**30iV**	32/64	32/64	32/64	32/64	32/32	64
**31eV**	16/16	32/32	16/16	16/16	16/32	32
**32V**	64/64	64/64	128/128	128/128	64/128	128
**33oV**	128/128	128/128	64/64	128/128	128/128	128
**34eV**	64/64	64/64	64/64	64/64	64/64	64
**35V**	64/64	64/64	128/128	64/128	64/128	128
**36oV**	64/128	128/128	64/64	128/128	64/128	128
**37eV**	64/64	64/64	64/64	64/64	64/64	64
**38V**	64/64	32/32	64/64	32/64	32/64	64
**39eV**	>128/>128	>128/>128	128/128	128/128	128/>128	>128
**40V**	64/64	128/128	128/128	64/64	64/128	128
**41oV**	64/64	16/16	64/64	32/32	32/64	64
**42oV**	4/4	4/4	16/32	8/8	4/16	32
**43oV**	128/>128	>128/>128	>128/>128	>128/>128	>128/>128	>128
**44oV**	128/128	128/128	128/128	64/64	128/128	128
**45oV**	128/>128	>128/>128	>128/>128	>128/>128	>128/>128	>128
**46oV**	16/32	64/128	32/32	32/64	32/64	128
**55oV**	16/32	8/8	16/32	8/8	8/16	32
*Reference Antibiotics*						
**Metronidazole (MTZ)**	256/256	2/2	1/1	32/32	2/256	256
**Clarithromycin (CLR)**	0.064/0.064	4/8	0.064/0.064	>256/>256	0.064/>256	>256
**Amoxicillin (AMX)**	0.016/0.016	0.064/0.064	0.016/0.016	0.064/0.064	0.016/0.064	0.064
Antibiotic susceptibility	MTZ^R^	MTZ^S^	MTZ^S^	MTZ^R^	-	-
	CLR^S^	CLR^R^	CLR^S^	CLR^R^	-	-
	AMX^S^	AMX^S^	AMX^S^	AMX^S^	-	-

MIC and MBC values are expressed in μg/mL as the average from experiments performed in triplicate. Antibiotic susceptibility was assessed according to the most recent EUCAST guidelines (Clinical Breakpoint Tables v. 16.0, valid from 1 January 2026) [62]. MTZ^R^ = metronidazole resistant (MIC > 8 μg/mL); MTZ^S^ = metronidazole susceptible (MIC ≤ 8 μg/mL); CLR^R^ = clarithromycin resistant (MIC > 0.25 μg/mL); CLR^S^ = clarithromycin susceptible (MIC ≤ 0.25 μg/mL); AMX^S^ = amoxicillin susceptible (MIC ≤ 0.125 μg/mL). Compounds **47V–54V** are previously reported azo derivatives [7].

**Table 2:** Selectivity indices of the most active vanilloid derivatives against *H. pylori*.

Compound	IC_50_ in GES-1 Cells (µg/mL)	MIC_50_ Against *H. pylori* NCTC 11637 (µg/mL)	Selectivity Index (SI)
**16V**	>64	4	>16
**20oV**	>64	8	>8
**29eV**	>64	16	>4

IC_50_ represents the concentration reducing GES-1 cell viability by 50% after 24 h of treatment. Since none of the compounds reduced cell viability below 50% within the investigated concentration range, IC_50_ values were reported as greater than the highest tested concentration of 64 µg/mL. The selectivity index was calculated as SI = IC_50_/MIC, using the MIC value determined against *H. pylori* NCTC 11637, and is therefore expressed as a lower-bound value.

**Table 3:** Antimicrobial activity of compounds **16V**, **20oV**, and **29eV** determined by the microdilution assay.

		MIC (µg/mL)			
Bacterial Strains		16V	20oV	29eV	CPX
Gram-negative	*E. coli* ATCC 35218	>128	>128	>128	0.0075
	*P. aeruginosa* ATCC 19660	>128	>128	>128	1
Gram-positive	*S. aureus* ATCC 29213	>128	>128	>128	0.25
	*S. aureus* ST25	>128	>128	>128	2

MIC values were determined by broth microdilution according to Clinical & Laboratory Standards Institute (CLSI) protocols, as described in Section 3. CPX, ciprofloxacin.

**Table 4:** The location of the studied vanilloids in the neural network compared to their locations on the HCA dendrogram.

HCA Cluster No.	Compounds	Data Set	Neuron Location	Activation	HCA Cluster No.	Compounds	Data Set	Neuron Location	Activation	HCA Cluster No.	Compounds	Data Set	Neuron Location	Activation
3	**oVan**	Train	(1, 1)	0.077	5	**Van**	External set	(1, 3)	0.622	2	**30iV**	Train	(2, 1)	0.127
3	**25iV**	External set	(1, 1)	0.029	5	**VAc**	Test	(1, 3)	0.668	2	**21eV**	Train	(2, 1)	0.131
3	**29eV**	Train	(1, 1)	0.039	5	**12eV**	Train	(1, 3)	0.713	2	**22V**	Train	(2, 1)	0.163
3	**49V**	Train	(1, 1)	0.063	5	**39eV**	Train	(1, 3)	0.713	2	**Visob**	Train	(2, 1)	0.172
3	**23V**	Test	(1, 1)	0.072	5	**18V**	Train	(1, 3)	0.754	2	**6oV**	Train	(2, 1)	0.172
3	**20oV**	Train	(1, 1)	0.074	4	**Vanalc**	Train	(1, 3)	0.372	2	**5V**	Train	(2, 1)	0.173
3	**53V**	Train	(1, 1)	0.083	4	**HA**	Train	(1, 3)	0.372	2	**13V**	Test	(2, 1)	0.173
3	**28oV**	Train	(1, 1)	0.090	4	**HValc**	Train	(1, 3)	0.372	2	**15eV**	Train	(2, 1)	0.173
3	**31eV**	Train	(1, 1)	0.093	4	**43oV**	Validation	(1, 3)	0.510	2	**26eV**	Test	(2, 1)	0.173
3	**52V**	Validation	(1, 1)	0.111	4	**45oV**	Train	(1, 3)	0.510	2	**37eV**	Train	(2, 1)	0.173
3	**50V**	Test	(1, 1)	0.112	4	**51V**	Train	(1, 3)	0.856	2	**34eV**	External set	(2, 1)	0.173
3	**27V**	External set	(1, 1)	0.117						2	**38V**	Train	(2, 1)	0.182
3	**42oV**	Validation	(1, 1)	0.119						2	**Vox**	Train	(2, 1)	0.197
3	**16V**	Train	(1, 1)	0.119						2	**1V**	Train	(2, 1)	0.210
3	**17eV**	Train	(1, 1)	0.142						2	**47V**	Validation	(2, 1)	0.254
3	**3A4HP**	Train	(1, 1)	0.159						2	**41oV**	Train	(2, 1)	0.271
3	**14oV**	Train	(1, 1)	0.159						2	**9V**	Train	(2, 1)	0.306
3	**AMX**	Train	(1, 1)	0.162						2	**46oV**	Train	(2, 1)	0.329
3	**24oV**	Test	(1, 1)	0.195						2	**8V**	Test	(2, 1)	0.455
1	**4eV**	Train	(1, 2)	0.198						1	**CLR**	Validation	(2, 1)	1.211
1	**7eV**	Train	(1, 2)	0.198						1	**MTZ**	Train	(2, 2)	0.000
1	**54V**	Train	(1, 2)	0.270						1	**11oV**	Train	(2, 3)	0.719
1	**19V**	Validation	(1, 2)	0.277						5	**3iV**	Train	(2, 3)	0.396
1	**33oV**	Train	(1, 2)	0.295						5	**2oV**	Train	(2, 3)	0.494
1	**36oV**	Validation	(1, 2)	0.353						5	**10V**	Validation	(2, 3)	0.569
1	**32V**	Train	(1, 2)	0.370										
1	**44oV**	External set	(1, 2)	0.405										
1	**35V**	Test	(1, 2)	0.447										
1	**40V**	Validation	(1, 2)	0.489										
5	**48V**	Test	(1, 2)	0.582										

Compounds **47V–54V** were previously reported vanillin-based azo derivatives included in Table S1 and reference [7].

## Data Availability

The original contributions presented in this study are included in the article and Supplementary Material. Further inquiries can be directed to the corresponding author.

## Supplementary Materials

The following supporting information can be downloaded at https://www.mdpi.com/article/10.3390/antibiotics15080729/s1: Table S1: Anti-*Hp* activity (MIC and MBC values) of previously synthesized vanillin-based azo compounds used for chemometric analyses; Table S2: Docking scores of the compound library and the reference compound; Table S3: Bond lengths and types formed by **16V** with amino acids in the residue region; Table S4: Bond lengths and types formed by the HAE compound with amino acids in the residue region. Additional in silico ADME prediction studies and Figures S1–S92: characterization NMR data for the newly synthesized compounds.
