# Supplementary material for: Synthesis, Antibacterial Evaluation, and Chemometric Profiling of a Vanilloid-Based Compounds Library Active Against Helicobacter pylori
Source: Antibiotics (Basel). 2026 Jul 27;15(8):729. doi: 10.3390/antibiotics15080729 (PMC13509194; doi:10.3390/antibiotics15080729)
Supplement: Supplementary file 1 [file antibiotics-15-00729-s001.zip › antibiotics-4361861-supplementary.pdf]

Table S1. Anti-*Hp* activity (MIC and MBC values) of previously synthesized vanillin-based azocompounds used for chemometric analyses.

		Structure	MIC/MBC (µg/mL)*				MIC_{50}/MIC_{90} (µg/mL)	MBC_{90} (µg/mL)
			F1	23	NCTC 11637	F4		
Van azo derivatives	47V	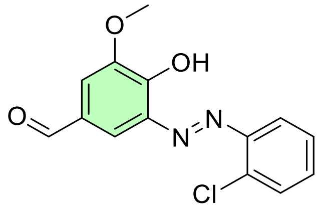	16/16	64/64	64/64	64/64	64/64	64
	48V	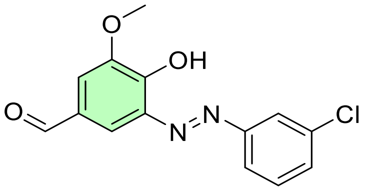	128/128	128/128	128/>128	128/128	128/>128	>128
	49V	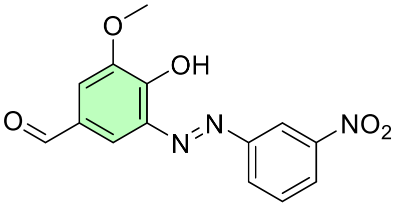	16/16	8/8	8/8	8/16	8/16	16
	50V	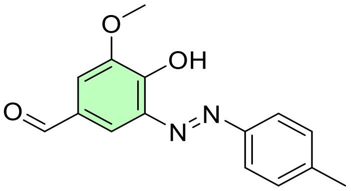	2/2	8/8	4/4	8/8	4/8	8
	51V	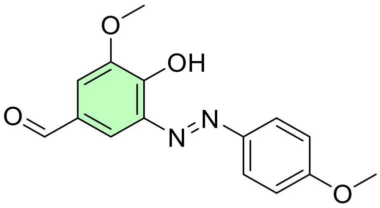	64/128	>128/>128	>128/>128	>128/>128	>128/>128	>128
	52V	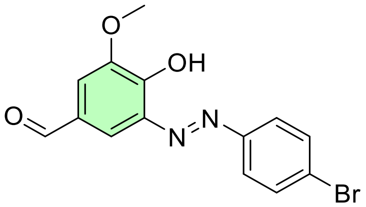	8/8	32/32	16/16	16/16	16/32	32
	53V	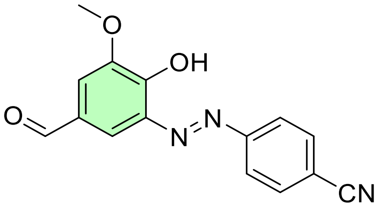	4/4	8/8	8/16	8/8	8/8	16
	54V	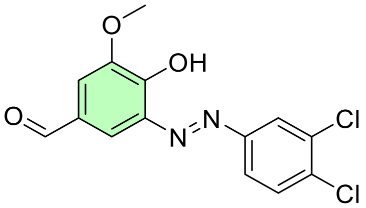	64 /64	64/128	128/128	128/128	64/128	128

Table S2. Docking scores of compound library and the reference compound.

Compound	Docking Score (kJ/mol)	Compound	Docking Score (kJ/mol)
**Acetohydroxamic acid**	-4.3	**46oV**	-4.5
**16V**	-8.2	**24oV**	-4.4
**17eV**	-7.7	**25iV**	-4.4
**10V**	-6.7	**28oV**	-4.2
**22V**	-6.3	**40V**	-4.1
**27V**	-6.2	**13V**	-3.7
**2oV**	-6.1	**26eV**	-3.4
**11oV**	-6.0	**18V**	-3.2
**12eV**	-6.0	**41oV**	-3.1
**1V**	-5.9	**14oV**	-2.9
**4eV**	-5.8	**45oV**	-2.9
**9V**	-5.8	**55oV**	-2.7
**42oV**	-5.7	**33oV**	-2
**29eV**	-5.7	**34eV**	-1.4
**3iV**	-5.7	**44oV**	-1.2
**5V**	-5.7	**37eV**	-1.1
**8V**	-5.6	**15eV**	1
**6oV**	-5.6	**43oV**	-0.9
**31eV**	-5.6	**36oV**	-0.6
**20oV**	-5.6	**39eV**	-0.5
**21eV**	-5.4	**38V**	-0.3
**7eV**	-5.4	**35V**	-0.2
**19V**	-5.3	**32V**	-0.2
**23V**	-4.8		
**30iV**	-4.6		

Table S3. Bond lengths and types formed by **16V** with amino acids in the residue region.

**Residue**	**Interaction Type**	**Distance (Å)**
Thr171	Hydrophobic	3.64
His221	Hydrophobic	3.81
His248	Hydrophobic	3.29
Phe273	Hydrophobic	3.00
Ala365	Hydrophobic	3.27
His136	H-bond	1.93
His138	H-bond	1.66
His221	H-bond	2.33
His274	Pi-Stacking	4.98

Table S4. Bond lengths and types formed by the HAE compound with amino acids in the residue region.

**Residue**	**Interaction Type**	**Distance (Å)**
Hıs248	Hydrophobic	3.59
Phe273	Hydrophobic	3.32
Hıs136	H-bond	3.18
Thr171	H-bond	2.08

Procedures and characterization data for thiosemicarbazones 1-4

The appropriate vanilloid (**Van**, **iVan**, **oVan**, and **eVan**) (1.0 eq) was dissolved in ethanol (0.5 M), followed by the addition of thiosemicarbazide (1.2 eq) and a catalytic amount of acetic acid. The stirring reaction mixture was placed in a microwave reactor and exposed to microwave irradiation at 110 °C for 15 min. The formed solid was washed with H_2O, Et_2O, and hexane (10 mL each) to afford compounds **1-4**, without requiring additional purification of the crude product.

1V. 2-(4-hydroxy-3-methoxybenzylidene)hydrazine-1-carbothioamide. White solid, m.p.: 198-200 °C, 99% yield. ^{1}H NMR (300 MHz, DMSO-*d*$_6$) δ 3.80 (s, 3H, OCH_3), 6.75 (d, J = 8.1 Hz, 1H, Ar), 7.01 (dd, J = 8.2, 1.9 Hz, 1H, Ar), 7.45 (d, J = 1.9 Hz, 1H, Ar), 7.91 (s, 1H, N=CH), 7.94 (s, 1H, NH_2), 8.10 (s, 1H, NH_2), 9.44 (s, 1H, Ar-OH), 11.24 (s, 1H, NHN=). ^{13}C NMR (75 MHz, DMSO-*d*$_6$) δ 56.2, 109.7, 115.6, 122.8, 126.0, 143.3, 148.5, 149.2, 177.8. Anal. calcd for $C_9H_{11}N_3O_2S$: C, 47.99; H, 4.92; N, 18.65. Found: C, 48.07; H, 4.94; N, 18.68.

2oV. 2-(2-hydroxy-3-methoxybenzylidene)hydrazine-1-carbothioamide. White solid, m.p.: 229-231 °C, 99% yield. ^{1}H NMR (300 MHz, DMSO-*d*$_6$) δ 3.78 (s, 3H, OCH_3), 6.74 (t, J = 8.0 Hz, 1H, Ar), 6.93 (dd, J = 8.0, 1.5 Hz, 1H, Ar), 7.50-7.53 (m, 1H, Ar), 7.87 (s, 1H, NH_2), 8.10 (s, 1H, NH_2), 8.38 (s, 1H, N=CH), 9.16 (s, 1H, Ar-OH), 11.39 (s, 1H, NHN=). ^{13}C NMR (75 MHz, DMSO-*d*$_6$) δ 56.3, 113.2, 118.6, 119.4, 121.2, 139.9, 146.4, 148.3, 178.1. Anal. calcd for $C_9H_{11}N_3O_2S$: C, 47.99; H, 4.92; N, 18.65. Found: C, 48.07; H, 4.94; N, 18.69.

3iV. 2-(3-hydroxy-4-methoxybenzylidene)hydrazine-1-carbothioamide. White solid, m.p.: 158-160 °C, 98% yield. ^{1}H NMR (300 MHz, DMSO-*d*$_6$) δ 3.78 (s, 3H, OCH_3), 6.91 (d, J = 8.4 Hz, 1H, Ar), 7.09 (dd, J = 8.4, 2.1 Hz, 1H, Ar), 7.24 (d, J = 2.0 Hz, 1H, Ar), 7.79 (s, 1H, NH_2), 7.89 (s, 1H, N=CH), 8.07 (s, 1H, NH_2), 9.03 (s, 1H, Ar-OH), 11.25 (s, 1H, NHN=). ^{13}C NMR (75 MHz, DMSO-*d*$_6$) δ 56.1, 112.2, 113.7, 120.5, 127.5, 143.2, 147.1, 150.0, 178.0. Anal. calcd for $C_9H_{11}N_3O_2S$: C, 47.99; H, 4.92; N, 18.65. Found: C, 47.94; H, 4.90; N, 18.67.

4eV. 2-(3-ethoxy-4-hydroxybenzylidene)hydrazine-1-carbothioamide. White solid, m.p.: 196-198 °C, 98% yield. ^{1}H NMR (300 MHz, DMSO-*d*$_6$) δ 1.31 (t, J = 6.9 Hz, 3H, $OCH_2\underline{CH_3}$), 4.05 (q, J = 6.9 Hz, 2H, $O\underline{CH_2}CH_3$), 6.76 (d, J = 8.1 Hz, 1H, Ar), 6.99 (dd, J = 8.2, 1.7 Hz, 1H, Ar), 7.43 (d, J = 1.8 Hz, 1H, Ar), 7.89 (s, 1H, N=CH), 7.93 (s, 1H, NH_2), 8.09 (s, 1H, NH_2), 9.36 (s, 1H, Ar-OH), 11.23 (s, 1H, NHN=). ^{13}C NMR (75 MHz, DMSO-*d*$_6$) δ 15.2, 64.3, 110.7, 115.6, 122.8, 126.0, 143.3, 147.7, 149.4, 177.7. Anal. calcd for $C_{10}H_{13}N_3O_2S$: C, 50.19; H, 5.48; N, 17.56. Found: C, 50.23; H, 5.50; N, 17.58.

Procedures and characterization data for *O*-alkylated derivatives 5-12

To a solution of vanillinoid (1.0 eq) in acetonitrile (0.4 M), the suitable alkyl halide (1.5 eq) and potassium carbonate (4.0 eq) were added, and the reaction mixture was stirred at reflux for 16 h. Then, the reaction mixture was poured into a sodium carbonate aqueous solution and extracted three times with dichloromethane (20 mL). The combined organic layers were dried over anhydrous sodium sulfate, filtered, and concentrated under reduced pressure to afford the crude product. Final purification was achieved by column flash chromatography on silica gel using mixtures of *n*-hexane and ethyl acetate as the eluent to afford the desired compounds.

5V. 3-methoxy-4-(prop-2-yn-1-yloxy)benzaldehyde. White solid, m.p. 89-91 °C. Characterization data according to Lenhart, J. A.; Ling, X.; Gandhi, R.; Guo, T. L.; Gerk, P. M.; Brunzell, D. H.; Zhang, S. "Clicked" Bivalent Ligands Containing Curcumin and Cholesterol as Multifunctional Abeta Oligomerization Inhibitors: Design, Synthesis, and Biological Characterization. J Med Chem 2010, 53 (16), 6198–6209. https://doi.org/10.1021/jm100601q.

6oV. 3-methoxy-2-(prop-2-yn-1-yloxy)benzaldehyde. White solid, m.p.: 51-52 °C, 80% yield. ^{1}H NMR (400 MHz, $CDCl_3$): δ 2.45-2.46 (t, J = 2.4 Hz, 1H, C≡CH), 3.89 (s, 3H, OCH_3), 4.86-4.87 (d, J = 2.4 Hz, 2H, OCH_2), 7.12-7.19 (m, 2H, Ar), 7.43-7.46 (m, 1H, 1H, Ar), 10.48 (s, 1H, CHO). ^{13}C NMR (100 MHz, $CDCl_3$): δ 56.0, 60.8, 78.2, 117.7, 118.9, 124.9, 132.6, 150.1, 154.2, 190.6. Anal. calcd for $C_{11}H_{10}O_3$: C, 69.46; H, 5.30. Found: C, 69.51; H, 5.34.

7eV. 3-ethoxy-4-(prop-2-yn-1-yloxy)benzaldehyde. White solid, m.p.: 77-79 °C, 71% yield. ^1H NMR (300 MHz, $CDCl_3$) *δ* 1.48 (t, *J* = 7.0 Hz, 3H, $OCH_2\underline{CH_3}$), 2.55 (t, *J* = 2.7 Hz, 1H, C≡CH), 4.16 (q, *J* = 7.0 Hz, 2H, $O\underline{CH_2}CH_3$), 4.85 (d, *J* = 2.4 Hz, 2H, OCH_2), 7.14 (d, *J* = 8.1 Hz, 1H, Ar), 7.41-7.46 (m, 2H, Ar), 9.85 (s, 1H, CHO). ^{13}C NMR (75 MHz, $CDCl_3$) *δ* 14.6, 56.6, 64.5, 110.7, 113.1, 126.0, 130.9, 190.9. Anal. calcd for $C_{12}H_{12}O_3$: C, 70.58; H, 5.92. Found: C, 70.63; H, 5.95.

8V. 4-(2-chloroethoxy)-3-methoxybenzaldehyde. Orangish solid, m.p.: 60-62 °C, 65% yield. ^1H NMR (600 MHz, $CDCl_3$): *δ* 3.87 (t, *J* = 6.2 Hz, 2H, $OCH_2\underline{CH_2}Cl$), 3.92 (s, 3H, OCH_3), 4.35 (t, *J* = 6.2 Hz, 2H, $O\underline{CH_2}CH_2Cl$), 6.98 (d, *J* = 8.0 Hz, 1H, Ar), 7.42-7.44 (m, 2H, Ar), 9.85 (s, 1H, CHO). ^{13}C NMR (151 MHz, $CDCl_3$) *δ* 41.3, 56.2, 69.0, 110.0, 112.5, 126.5, 131.0, 150.2, 153.2, 191.0. Anal. calcd for $C_{10}H_{11}ClO_3$: C, 55.96; H, 5.17. Found: C, 56.01; H, 5.19.

9V. 4-(3-chloropropoxy)-3-methoxybenzaldehyde. Colorless oil, 56% yield. ^1H NMR (300 MHz, $CDCl_3$) *δ* 2.28-2.37 (m, 2H, $OCH_2\underline{CH_2}CH_2Cl$), 3.77 (t, *J* = 6.2 Hz, 2H, $OCH_2CH_2\underline{CH_2}Cl$), 3.91 (s, 3H, OCH_3), 4.25 (t, *J* = 6.0 Hz, 2H, $O\underline{CH_2}CH_2CH_2Cl$), 7.00 (d, *J* = 8.1 Hz, 1H, Ar), 7.40-7.46 (m, 2H, Ar), 9.85 (s, 1H, CHO). ^{13}C NMR (75 MHz, $CDCl_3$) *δ* 31.9, 41.3, 56.0, 65.5, 109.4, 111.7, 126.7, 130.3, 149.9, 153.7, 190.9. Anal. calcd for $C_{11}H_{13}ClO_3$: C, 57.78; H, 5.73. Found: C, 57.71; H, 5.70.

10V. ethyl 2-(4-formyl-2-methoxyphenoxy)acetate. Orangish solid, m.p.: 63-65 °C, 72% yield. ^1H NMR (600 MHz, $CDCl_3$): *δ* 1.24-1-30 (m, 3H, $OCH_2\underline{CH_3}$), 3.94 (s, 1H, OCH_3), 4.26 (q, 2H, $O\underline{CH_2}CH_3$), 4.76 (s, 2H, $OCH_2C{=}O$), 6.87 (t, *J* = 18.0 Hz, 1H, Ar), 7.40-7.43 (m, 2H, Ar), 9.85 (s, 1H, CHO). ^{13}C NMR (151 MHz, $CDCl_3$): *δ* 14.2, 56.2, 61.7, 66.1, 110.0, 112.5, 126.2, 131.3, 150.1, 152.7, 168.1, 190.9. Anal. calcd for $C_{12}H_{14}O_5$: C, 60.50; H, 5.92. Found: C, 60.45; H, 5.95.

11oV. ethyl 2-(2-formyl-6-methoxyphenoxy)acetate. White solid, m.p.: 135-137 °C, 69% yield. ^1H NMR (600 MHz, $CDCl_3$): *δ* 1.24-1.28 (m, 3H, $OCH_2\underline{CH_3}$), 3.88 (s, 3H, OCH_3), 4.21 (q, 2H, OCH_2), 4.82 (s, 2H, $O\underline{CH_2}CH_3$), 7,12-7.13 (m, 2H, Ar), 7,43-7,45 (m, 1H, Ar), 10.62 (s, 1H, CHO). ^{13} C NMR (151 MHz, $CDCl_3$): *δ* 14.2, 56.2, 61.3, 69.7, 118.0, 119.3, 124.4, 130.0, 150.2, 152.0, 169.4, 190.8. Anal. calcd for $C_{12}H_{14}O_5$: C, 60.50; H, 5.92. Found: C, 60.55; H, 5.95.

12eV. ethyl 2-(2-ethoxy-4-formylphenoxy)acetate. White solid, m.p.: 69-71 °C, 55% yield. ^1H NMR (300 MHz, $CDCl_3$) *δ* 1.28 (t, *J* = 7.1 Hz, 3H, $COCH_2\underline{CH_3}$), 1.48 (t, *J* = 7.0 Hz, 3H, $OCH_2\underline{CH_3}$), 4.17 (q, *J* = 7.0 Hz, 2H, $O\underline{CH_2}CH_3$), 4.26 (q, *J* = 7.1 Hz, 2H, $CO\underline{CH_2}CH_3$), 4.77 (s, 2H, $OCH_2C{=}O$), 6.88 (d, *J* = 8.1 Hz, 1H, Ar), 7.38-7.42 (m, 2H, Ar), 9.84 (s, 1H, CHO). ^{13}C NMR (75 MHz, $CDCl_3$) *δ* 14.1, 14.6, 61.5, 64.6, 66.1, 111.2, 113.1, 125.9, 131.2, 149.4, 152.8, 168.2, 190.9. Anal. calcd for $C_{13}H_{16}O_5$: C, 61.90; H, 6.39. Found: C, 61.86; H, 6.34.

Procedures and characterization data for *O*-alkylated dimers 13-15 and *O*-benzylated 32-37

To a solution of vanillinoid (2.0 eq) in acetonitrile (0.4 M), the suitable alkyl/benzyl halide (1.0 eq) and potassium carbonate (4.0 eq) were added, and the reaction mixture was stirred at reflux for 16 h. Then, the reaction mixture was poured into a sodium carbonate aqueous solution and extracted three times with dichloromethane (20 mL). The combined organic layers were dried over anhydrous sodium sulfate, filtered, and concentrated under reduced pressure to afford the crude product. Final purification was achieved by column flash chromatography on silica gel using mixtures of *n*-hexane and ethyl acetate as the eluent to afford the desired compounds.

13V. 4,4'-(pentane-1,5-diylbis(oxy))bis(3-methoxybenzaldehyde. Yellow solid, m.p.: 109-111 °C., 49% yield. ^1H NMR (400 MHz, $CDCl_3$): *δ* 1.59-1.71 (m, 2H, CH_2), 1.95-1.99 (m, 4H, $2CH_2$), 3.90 (s, 6H, 2 OCH_3), 4.12-4.14 (t, *J* = 4.0 Hz, 4H, $2OCH_2$), 6.95 (d, *J* = 8 Hz, 2H, Ar), 7.39 (s, 2H, Ar), 7.40-7.43 (m, 2H, Ar), 9.83 (s, 2H, CHO). ^{13}C NMR (101 MHz, $CDCl_3$): *δ* 22.6, 28.8, 56.1, 68.9, 109.4, 111.5, 126.8, 130.1, 150.0, 154.1, 191.0. Anal. calcd for $C_{21}H_{24}O_6$: C, 67.73; H, 6.50. Found: C, 67.68; H, 6.49.

14oV. 2,2'-(pentane-1,5-diylbis(oxy))bis(3-methoxybenzaldehyde). Yellow oil, 47% yield. ^1H NMR (400 MHz, $CDCl_3$): *δ* 1.66-1.70 (m, 2H, CH_2), 1.86-1.90 (m, 4H, $2CH_2$), 3.88 (s, 6H, 2 OCH_3), 4.14-4.16 (t, *J* = 4.0 Hz, 4H, 2 x OCH_2), 7.09-7.14 (m, 4H, Ar), 7.41 (d, *J* = 4.0 Hz, 2H, Ar), 10.44 (s, 1H, CHO),

10.44 (s, 1H, CHO). ^{13}C NMR (101 MHz, $CDCl_3$): δ 22.5, 29.9, 56.1, 74.8, 118.2, 119.3, 124.1, 130.1, 152.1, 153.2, 190.4. Anal. calcd for $C_{21}H_{24}O_6$: C, 67.73; H, 6.50. Found: C, 67.70; H, 6.55.

15eV. 4,4'-(pentane-1,5-diylbis(oxy))bis(3-ethoxybenzaldehyde). White solid, m.p.: 94-96 °C, 72% yield. ^{1}H NMR (300 MHz, $CDCl_3$) δ 1.45 (t, J = 7.0 Hz, 6H, 2 x $OCH_2\underline{CH_3}$), 1.67-1.75 (m, 2H, $OCH_2CH_2\underline{CH_2}CH_2CH_2O$), 1.98 (dt, J = 14.4, 6.7 Hz, 4H, $OCH_2\underline{CH_2}CH_2\underline{CH_2}CH_2O$), 4.09-4.16 (m, 8H, $O\underline{CH_2}CH_2CH_2CH_2\underline{CH_2}O$ and 2 x $O\underline{CH_2}CH_3$), 6.95 (d, J = 8.0 Hz, 2H, Ar), 7.38-7.43 (m, 4H, Ar), 9.82 (s, 2H, 2 x CHO). ^{13}C NMR (75 MHz, $CDCl_3$) δ 14.7, 22.6, 28.6, 64.5, 68.9, 110.8, 111.8, 126.5, 130.0, 149.2, 154.4, 190.9. Anal. calcd for $C_{23}H_{28}O_6$: C, 68.98; H, 7.05. Found: C, 68.99; H, 7.07.

32V. 4,4'-((1,2-phenylenebis(methylene))bis(oxy))bis(3-methoxybenzaldehyde). White solid, m.p.: 151-153 °C, 82% yield. ^{1}H NMR (300 MHz, $CDCl_3$): δ 3.89 (s, 6H, 2 x OCH_3), 5.38 (s, 4H, 2 x OCH_2), 7.04 (d, J = 8.4 Hz, 2H, Ar), 7.35-7.40 (m, 6H, Ar), 7.48-7.51 (m, 2H, Ar), 9.83 (s, 2H, 2 x CHO). ^{13}C NMR (75 MHz, $CDCl_3$): δ 55.9, 69.3, 109.3, 112.3, 126.4, 128.7, 128.9, 130.5, 134.2, 150.1, 153.3, 190.8. Anal. calcd for $C_{24}H_{22}O_6$: C, 70.93; H, 5.46. Found: C, 70.98; H, 5.50.

33oV. 2,2'-((1,2-phenylenebis(methylene))bis(oxy))bis(3-methoxybenzaldehyde). White solid, m.p.: 147-149 °C. 91% yield. ^{1}H NMR (300 MHz, $CDCl_3$): δ 3.88 (s, 6H, 2 x OCH_3), 5.33 (s, 4H, 2 x OCH_2), 7.12-7.18 (m, 4H, Ar), 7.36-7.40 (m, 4H, Ar), 7.45-7.49 (m, 2H, Ar), 10.21 (s, 2H, 2 x CHO). ^{13}C NMR (75 MHz, $CDCl_3$): δ 56.0, 73.7, 118.0, 119.2, 124.4, 129.0, 130.2, 135.2, 151.1, 153.1, 190.0. Anal. calcd for $C_{24}H_{22}O_6$: C, 70.93; H, 5.46. Found: C, 70.98; H, 5.50.

34eV. 4,4'-((1,2-phenylenebis(methylene))bis(oxy))bis(3-ethoxybenzaldehyde). White solid, m.p.: 128-130 °C. 85% yield. ^{1}H NMR (300 MHz, $CDCl_3$) δ 1.43 (t, J = 7.0 Hz, 6H, 2 x $OCH_2\underline{CH_3}$), 4.11 (q, J = 7.0 Hz, 4H, 2 x $O\underline{CH_2}CH_3$), 5.39 (s, 4H, 2 x OCH_2), 7.02 (d, J = 8.7 Hz, 2H, Ar), 7.35-7.39 (m, 6H, Ar), 7.50-7.53 (m, 2H, Ar), 9.81 (s, 2H, 2 x CHO). ^{13}C NMR (75 MHz, $CDCl_3$) δ 14.7, 64.3, 69.4, 110.5, 112.6, 126.1, 128.5, 128.6, 130.5, 134.3, 149.4, 153.5, 190.9. Anal. calcd for $C_{26}H_{26}O_6$: C, 71.87; H, 6.03. Found: C, 71.82; H, 6.07.

35V. 4,4'-((1,4-phenylenebis(methylene))bis(oxy))bis(3-methoxybenzaldehyde). White solid, m.p.: 155-157 °C. 95% yield. ^{1}H NMR (300 MHz, $CDCl_3$): δ 3.94 (s, 6H, 2 x OCH_3), 5.24 (s, 4H, 2 x OCH_2), 6.98 (d, J = 8.1 Hz, 2H, Ar), 7.37-7.43 (m, 4H, Ar), 7.46 (s, 4H, Ar), 9.84 (s, 2H, 2 x CHO). ^{13}C NMR (75 MHz, $CDCl_3$): δ 56.1, 70.5, 109.4, 110.0, 112.4, 126.5, 127.6, 130.4, 136.1, 150.1, 153.5, 190.9. Anal. calcd for $C_{24}H_{22}O_6$: C, 70.93; H, 5.46. Found: C, 70.89; H, 5.42.

36oV. 2,2'-((1,4-phenylenebis(methylene))bis(oxy))bis(3-methoxybenzaldehyde). White solid, m.p.: 157-159 °C, 70% yield. ^{1}H NMR (300 MHz, $CDCl_3$): δ 3.94 (s, 6H, 2 x OCH_3), 5.18 (s, 4H, 2 x OCH_2), 7.11-7.20 (m, 4H, Ar), 7.37-7.40 (m, 6H, Ar), 10.25 (s, 2H, 2 x CHO). ^{13}C NMR (75 MHz, $CDCl_3$): δ 56.1, 75.9, 118.0, 119.1, 124.3, 128.8, 130.2, 136.7, 151.0, 153.0, 190.2. Anal. calcd for $C_{24}H_{22}O_6$: C, 70.93; H, 5.46. Found: C, 70.99; H, 5.42.

37eV. 4,4'-((1,4-phenylenebis(methylene))bis(oxy))bis(3-ethoxybenzaldehyde). White solid, m.p.: 152-154 °C, 42% yield. ^{1}H NMR (300 MHz, $CDCl_3$) δ 1.48 (t, J = 7.0 Hz, 6H, 2 x $OCH_2\underline{CH_3}$), 4.17 (q, J = 7.0 Hz, 4H, 2 x $O\underline{CH_2}CH_3$), 5.24 (s, 4H, 2 x OCH_2), 6.98 (d, J = 8.7 Hz, 2H, Ar), 7.38 (dd, J = 8.4, 2.4 Hz, 2H, Ar), 7.42 (d, J = 1.8 Hz, 2H, Ar), 7.46 (s, 4H, Ar), 9.83 (s, 2H, 2 x CHO). ^{13}C NMR (75 MHz, $CDCl_3$) δ 14.7, 64.6, 70.6, 110.9, 112.9, 126.3, 127.4, 130.5, 136.2, 149.5, 153.8, 190.9. Anal. calcd for $C_{26}H_{26}O_6$: C, 71.87; H, 6.03. Found: C, 71.92; H, 6.06.

Procedures and characterization data for *O*-benzylated 16, 17, and 19-29 and *O*-naphthylmethylated 30 and 31 vanilloids

To a solution of vanillinoid (1.0 eq) in acetonitrile (0.4 M), the suitable benzyl or naphthylmethyl halide (3.0 eq) and potassium carbonate (4.0 eq) were added, and the reaction mixture was stirred at reflux for 16 h. Then, the reaction mixture was poured into a sodium carbonate aqueous solution and extracted three times with dichloromethane (20 mL). The combined organic layers were dried over anhydrous sodium sulfate, filtered, and concentrated under reduced pressure to afford the

crude product. Final purification was achieved by column flash chromatography on silica gel using mixtures of *n*-hexane and ethyl acetate as the eluent to afford the desired compounds.

16V. 3-methoxy-4-((5-phenyl-1,3,4-oxadiazol-2-yl)methoxy)benzaldehyde. White solid, m.p.: 152-154 °C, 41% yield. ^{1}H NMR (600 MHz, $CDCl_3$): *δ* 3.94 (s, 3H, OCH_3), 5.48 (s, 2H, CH_2), 7.24-7.26 (m, 1H, Ar), 7.44-7.45 (m, 2H, Ar), 7.49-7.51 (m, 2H, Ar), 7.53-7.56 (m, 1H, Ar), 8.05-8.06 (m, 2H, Ar), 9.87 (s, 1H, CHO). ^{13}C NMR (151 MHz, $CDCl_3$) *δ* 59.2, 61.0, 110.0, 113.7, 123.5, 126.3, 127.3, 129.2, 131.8, 132.3, 150.4, 152.2, 161.7, 166.1, 190.9. Anal. calcd for $C_{17}H_{14}N_2O_4$: C, 65.80; H, 4.55; N, 9.03. Found: C, 65.85; H, 4.56; N, 9.05.

17eV. 3-ethoxy-4-((5-phenyl-1,3,4-oxadiazol-2-yl)methoxy)benzaldehyde. White solid, m.p.: 120-122 °C, 55% yield. ^{1}H NMR (300 MHz, $CDCl_3$) *δ* 1.47 (t, *J* = 7.0 Hz, 3H, $OCH_2\underline{CH_3}$), 4.17 (q, *J* = 7.0 Hz, 2H, $O\underline{CH_2}CH_3$), 5.48 (s, 2H, OCH_2), 7.24 (d, *J* = 8.8 Hz, 1H, Ar), 7.41-7.56 (m, 5H, Ar), 8.04-8.08 (m, 2H, Ar), 9.86 (s, 1H, CHO), ^{13}C NMR (75 MHz, $CDCl_3$) *δ* 14.6, 61.1, 64.6, 111.1, 114.5, 123.4, 126.0, 127.1, 129.1, 131.8, 132.2, 149.8, 152.3, 161.8, 165.9, 190.9. Anal. calcd for $C_{18}H_{16}N_2O_4$: C, 66.66; H, 4.97; N, 8.64. Found: C, 66.71; H, 4.94; N, 8.62.

19V. 3-methoxy-4-((3-nitrobenzyl)oxy)benzaldehyde. Yellow solid, m.p.: 151-153 °C, 62% yield. 1H NMR (400 MHz, $CDCl_3$) *δ* 3.97 (d, *J* = 1.8 Hz, 2H), 5.31 (s, 1H), 6.99 (s, 2H), 7.42 (dd, *J* = 8.1, 1.9 Hz, 2H), 7.44 (s, 5H), 7.59 (t, *J* = 7.9 Hz, 2H), 7.80 (d, *J* = 7.6 Hz, 2H), 8.20 (d, *J* = 8.1 Hz, 2H), 8.34 (s, 1H), 9.87 (d, *J* = 8.1 Hz, 2H). ^{13}C NMR (101 MHz, $CDCl_3$) *δ* 56.2, 69.8, 109.8, 112.8, 122.3, 123.4, 126.4, 130.0, 131.1, 133.2, 138.4, 148.7, 150.4, 153.0, 191.0. Anal. calcd for $C_{15}H_{13}NO_5$: C, 62.72; H, 4.56; N, 4.88. Found: C, 62.68; H, 4.53; N, 4.85.

20oV. 3-methoxy-2-((3-nitrobenzyl)oxy)benzaldehydec. White solid, m.p.: 83-85 °C, 52% yield. ^{1}H NMR (300 MHz, $CDCl_3$) *δ* 3.95 (s, 3H, OCH_3), 5.25 (s, 2H, OCH_2), 7.18-7.20 (m, 2H, Ar), 7.40-7.43 (m, 1H, Ar), 7.54-7.60 (m, 1H, Ar), 7.77-7.81 (m, 1H, Ar), 8.19-8.24 (m, 1H, Ar), 8.34 (tt, *J* = 1.7, 0.7 Hz, 1H, Ar), 10.34 (s, 1H, CHO). ^{13}C NMR (75 MHz, $CDCl_3$) *δ* 56.1, 74.7, 118.1, 119.7, 123.0, 123.2, 124.8, 129.6, 130.0, 133.3, 133.9, 138.9, 150.4, 152.8, 189.6. Anal. calcd for $C_{15}H_{13}NO_5$: C, 62.72; H, 4.56; N, 4.88. Found: C, 62.78; H, 4.59; N, 4.85.

21eV. 3-ethoxy-4-((3-nitrobenzyl)oxy)benzaldehyde. White solid, m.p.: 115-117 °C, 61% yield. ^{1}H NMR (300 MHz, $CDCl_3$) *δ* 1.51 (t, *J* = 7.0 Hz, 3H, $OCH_2\underline{CH_3}$), 4.19 (q, *J* = 7.0 Hz, 2H, $O\underline{CH_2}CH_3$), 5.31 (s, 2H, OCH_2), 6.99 (d, *J* = 8.1 Hz, 1H, Ar), 7.39-7.44 (m, 2H, Ar), 7.58 (t, *J* = 7.9 Hz, 1H, Ar), 7.79 (ddq, *J* = 7.7, 1.7, 0.8 Hz, 1H, Ar), 8.19 (ddd, *J* = 8.2, 2.4, 1.0 Hz, 1H, Ar), 8.37 (tt, *J* = 1.6, 0.7 Hz, 1H, Ar), 9.85 (s, 1H, CHO). ^{13}C NMR (75 MHz, $CDCl_3$) *δ* 14.7, 64.6, 69.6, 111.0, 113.2, 122.0, 123.1, 126.1, 129.7, 131.0, 132.8, 138.6, 149.6, 153.1,190.9. Anal. calcd for $C_{16}H_{15}NO_5$: C, 63.78; H, 5.02; N, 4.65. Found: C, 63.84; H, 5.00; N, 4.69.

22V. 3-methoxy-4-((4-nitrobenzyl)oxy)benzaldehyde. Yellow solid, m.p.: 119-120 °C, 79% yield. ^{1}H NMR (400 MHz, $CDCl_3$): *δ* 3.96 (s, 3H, OCH_3), 5.32 (s, 2H, OCH_2), 6.95 (d, *J* = 4.0 Hz, 1H, Ar), 7.39-7.41 (m, 1H, Ar), 7.45 (m, 1H, Ar), 7.62 (d, *J* = 8.0 Hz, 2H, Ar), 8.25 (d, *J* = 4.0 Hz, 2H, Ar), 9.85 (s, 1H, CHO). ^{13}C NMR (101 MHz, $CDCl_3$): *δ* 56.2, 69.7, 109.8, 112.6, 124.1, 126.4, 127.6, 127.6, 131.0, 143.5, 147.9, 150.3, 152.9, 190.9. Anal. calcd for $C_{15}H_{13}NO_5$: C, 62.72; H, 4.56; N, 4.88. Found: C, 62.76; H, 4.52; N, 4.83.

23V. 3-methoxy-4-((3-(trifluoromethyl)benzyl)oxy)benzaldehyde. White solid, m.p.: 57-59 °C, 74% yield. ^{1}H NMR (400 MHz, $CDCl_3$): *δ* 3.95 (s, 3H, OCH_3), 5.26 (s, 2H, OCH_2), 6.98 (d, *J* = 4.0 Hz, 1H, Ar), 7.40-7.41 (m, 1H, Ar), 7.44 (m, 1H, Ar), 7.50-7.52 (t, *J* = 4.0 Hz, 1H, Ar), 7.58-7.60 (m, 1H, Ar), 7.62-7.64 (m, 1H, Ar), 7.71 (m, 1H, Ar), 9.85 (s, 1H, CHO). ^{13}C NMR (101 MHz, $CDCl_3$): *δ* 56.2, 70.3, 109.7, 112.7, 124.1, 124.1, 124.1, 125.2, 125.2, 125.2, 126.5, 129.4, 130.6, 130.8, 137.2, 150.3, 153.3, 190.9. Anal. calcd for $C_{16}H_{13}F_3O_3$: C, 61.94; H, 4.22. Found: C, 61.904; H, 4.20.

24oV. 3-methoxy-2-((3-(trifluoromethyl)benzyl)oxy)benzaldehyde. White solid, m.p.: 60-62 °C, 72% yield. ^{1}H NMR (300 MHz, $CDCl_3$) *δ* 3.93 (s, 3H, OCH_3), 5.22 (s, 2H, OCH_2), 7.17.-7.18 (m, 2H, Ar), 7.40-7.43 (m, 1H, Ar), 7.48-7.53 (m, 1H, Ar) 7.59-7.64 (m, 2H, Ar), 7.71 (s, 1H, Ar), 10.32 (s, 1H,

CHO). ^{13}C NMR (75 MHz, $CDCl_3$) δ 56.1, 75.4, 118.1, 119.5, 124.6, 125.1, 125.2, 129.1, 130.1, 131.4, 131.4, 137.7, 150.7, 152.9, 189.8. Anal. calcd for $C_{16}H_{13}F_3O_3$: C, 61.94; H, 4.22. Found: C, 61.99; H, 4.25.

25iV. 4-methoxy-3-((3-(trifluoromethyl)benzyl)oxy)benzaldehyde. White solid, m.p.: 79-81 °C, 66% yield. ^{1}H NMR (300 MHz, $CDCl_3$) δ 3.97 (s, 3H, OCH_3), 5.22 (s, 2H, OCH_2), 7.02 (d, J = 8.2 Hz, 1H, Ar), 7.45-7.53 (m, 3H, Ar), 7.57-7.65 (m, 2H, Ar), 7.74 (s, 1H, Ar), 9.83 (s, 1H, CHO). ^{13}C NMR (75 MHz, $CDCl_3$) δ 56.2, 70.3, 110.9, 111.5, 124.2, 124.2, 125.0, 125.0, 127.4, 129.1, 130.0, 130.7, 137.4, 148.4, 155.1, 190.7. Anal. calcd for $C_{16}H_{13}F_3O_3$: C, 61.94; H, 4.22. Found: C, 62.00; H, 4.24.

26eV. 3-ethoxy-4-((3-(trifluoromethyl)benzyl)oxy)benzaldehyde. White solid, m.p.: 86-88 °C, 94% yield. ^{1}H NMR (300 MHz, $CDCl_3$) δ 1.49 (t, J = 7.0 Hz, 3H, $OCH_2\underline{CH_3}$), 4.18 (q, J = 7.0 Hz, 2H, $O\underline{CH_2}CH_3$), 5.27 (s, 2H, OCH_2), 6.99 (d, J = 8.1 Hz, 1H, Ar), 7.39-7.44 (m, 2H, Ar), 7.48-7.53 (m, 1H, Ar), 7.58-7.64 (m, 2H, Ar), 7.75 (s, 1H, Ar), 9.85 (s, 1H, CHO). ^{13}C NMR (75 MHz, $CDCl_3$) δ 14.6, 64.6, 70.2, 111.1, 113.2, 123.8, 124.9, 126.1, 129.1, 130.2, 130.8, 137.4, 149.6, 153.4, 190.9. Anal. calcd for $C_{17}H_{15}F_3O_3$: C, 62.96; H, 4.66. Found: C, 63.02; H, 4.69.

27V. 4-((4-bromobenzyl)oxy)-3-methoxybenzaldehyde. Pale pink solid, m.p.: 98-99 °C, 67% yield. ^{1}H NMR (400 MHz, $CDCl_3$): δ 3.94 (s, 3H, OCH_3), 5.17 (s, 2H, OCH_2), 6.95 (d, J = 4.0 Hz, 1H, Ar), 7.30-7.31 (m, 2H, Ar), 7.37-7.39 (m, 1H, Ar), 7.42 (m, 1H, Ar), 7.49-7.51 (m, 2H, Ar), 9.83 (s, 1H, CHO). ^{13}C NMR (101 MHz, $CDCl_3$): δ 56.2, 70.3, 109.6, 112.6, 122.3, 126.5, 129.0, 130.7, 131.9, 132.0, 135.2, 150.2, 153.4, 191.0. Anal. calcd for $C_{15}H_{13}BrO_3$: C, 56.10; H, 4.08. Found: C, 56.16; H, 4.11.

28oV. 2-((4-bromobenzyl)oxy)-3-methoxybenzaldehyde. White solid, m.p.: 79-80 °C, 55% yield. ^{1}H NMR (400 MHz, $CDCl_3$): δ 3.92 (s, 3H, OCH_3), 5.11 (s, 2H, OCH_2), 7.13-7.18 (m, 2H, Ar), 7.26-7.27 (m, 2H, Ar), 7.38-7.39 (m, 1H, Ar), 7.48-7.49 (d, J = 4.0 Hz, 2H, Ar), 10.26 (s, 1H, Ar). ^{13}C NMR (101 MHz, $CDCl_3$): δ 56.2, 75.5, 118.2, 119.4, 122.6, 124.5, 130.2, 130.3, 131.8, 135.6, 150.9, 153.0, 190.1. Anal. calcd for $C_{15}H_{13}BrO_3$: C, 56.10; H, 4.08. Found: C, 56.05; H, 4.06.

29eV. 4-((4-bromobenzyl)oxy)-3-ethoxybenzaldehyde. White solid, m.p.: 80-82 °C, 61% yield. ^{1}H NMR (300 MHz, $CDCl_3$) δ 1.48 (t, J = 7.0 Hz, 3H, $OCH_2\underline{CH_3}$), 4.17 (q, J = 7.0 Hz, 2H, $O\underline{CH_2}CH_3$), 5.18 (s, 2H, OCH_2), 6.95 (d, J = 8.1 Hz, 1H, Ar), 7.30-7.33 (m, 2H, Ar), 7.36-7.42 (m, 2H, Ar), 7.49-7.53 (m, 2H, Ar), 9.83 (s, 1H, CHO). ^{13}C NMR (75 MHz, $CDCl_3$) δ 14.7, 64.6, 70.1, 110.9, 112.9, 122.0, 126.2, 128.7, 130.6, 131.8, 135.3, 149.5, 153.5, 190.9. Anal. calcd for $C_{16}H_{15}BrO_3$: C, 57.33; H, 4.51. Found: C, 57.39; H, 4.55.

30iV. 4-methoxy-3-(naphthalen-1-ylmethoxy)benzaldehyde. White solid, m.p.: 125-127 °C, 61% yield. ^{1}H NMR (300 MHz, $CDCl_3$) δ 3.93 (s, 3H, OCH_3), 5.61 (s, 2H, OCH_2), 7.01 (d, J = 8.3 Hz, 1H, Ar), 7.44-7.65 (m, 6H, Ar), 7.84-7.91 (m, 2H, Ar), 8.07-8.10 (m, 1H, Ar), 9.84 (s, 1H, CHO). ^{13}C NMR (75 MHz, $CDCl_3$) δ 56.2, 69.6, 111.0, 111.9, 123.6, 125.3, 125.9, 126.5, 126.6, 127.0, 128.7, 129.1, 130.1, 131.5, 131.7, 133.8, 148.9, 155.3, 190.8. Anal. calcd for $C_{19}H_{16}O_3$: C, 78.06; H, 5.52. Found: C, 78.02; H, 5.47.

31eV. 3-ethoxy-4-(naphthalen-1-ylmethoxy)benzaldehyde. White solid, m.p.: 88-90 °C, 49% yield. ^{1}H NMR (300 MHz, $CDCl_3$) δ 1.45 (t, J = 7.0 Hz, 3H, $OCH_2\underline{CH_3}$), 4.16 (q, J = 7.0 Hz, 2H, $O\underline{CH_2}CH_3$), 5.68 (s, 2H, OCH_2), 7.09 (d, J = 8.2 Hz, 1H, Ar), 7.39 (dd, J = 8.2, 1.9 Hz, 1H, Ar), 7.44-7.48 (m, 2H, Ar), 7.50-7.57 (m, 2H, Ar), 7.59-7.62 (m, 1H, Ar), 7.83-7.92 (m, 2H, Ar), 8.08-8.12 (m, 1H, Ar), 9.84 (s, 1H, CHO). ^{13}C NMR (75 MHz, $CDCl_3$) δ 14.7, 64.6, 69.7, 111.1, 113.5, 123.4, 125.3, 126.0, 126.0, 126.3, 126.4, 128.8, 129.0, 130.6, 131.2, 131.5, 133.7, 149.7, 153.9, 191.0. Anal. calcd for $C_{20}H_{18}O_3$: C, 78.41; H, 5.92. Found: C, 78.49; H, 5.95.

Procedures and characterization data for derivative 18V

Compound **16V** (1.0 eq) was dissolved in ethanol (0.5 M), followed by the addition of thiosemicarbazide (1.2 eq) and a catalytic amount of acetic acid. The stirring reaction mixture was placed in a microwave reactor and exposed to microwave irradiation at 110 °C for 15 min. The formed solid was washed with H_2O, Et_2O, and hexane (10 mL each) without requiring additional purification of the crude product.

18V. 2-(3-methoxy-4-((5-phenyl-1,3,4-oxadiazol-2-yl)methoxy)benzylidene)hydrazine-1-carbothioamide. White solid, m.p.: 227-229 °C, 88% yield. ^{1}H NMR (300 MHz, DMSO-d_6) δ 3.82 (s, 3H, OCH_3), 5.48 (s, 2H, OCH_2), 7.17 (s, 2H, Ar), 7.56 -7.68 (m, 4H, Ar), 7.96-8.02 (m, 3H, N=CH and 2 Ar), 8.08 (s, 1H, NH_2), 8.22 (s, 1H, NH_2), 11.39 (s, 1H, NHN=). ^{13}C NMR (75 MHz, DMSO-d_6) δ 56.3, 60.8, 109.6, 114.6, 122.2, 123.4, 127.1, 129.2, 129.3, 130.0, 132.8, 142.5, 148.6, 150.1, 150.1, 163.0, 165.3, 178.1. Anal. calcd for $C_{18}H_{17}N_5O_3S$: C, 56.39; H, 4.47; N, 18.27. Found: C, 56.43; H, 4.45; N, 18.30.

Procedures and characterization data for esters 38V, 39eV, and 55oV

To a solution of suitable vanilloid (**Van**, **oV**, or **eVan**) (1 eq) in dichloromethane (0.2 M), triethylamine (TEA) (2.0 eq) and dimethylaminopyridine (DMAP) (0.1 eq) were added at 0 °C. Then, 1-adamanthanecarbonyl chloride (1.1 eq) was added, and the mixture was allowed to reach room temperature. After 48 h, the reaction was diluted with dichloromethane (50 mL) and extracted with distilled water (3 x 20 mL). The organic layer was dried on anhydrous sodium sulfate and evaporated under reduced pressure. Crude material was purified by column flash chromatography on silica gel, using ethyl acetate as eluent.

38V. 4-Formyl-2-methoxyphenyl adamantane-1-carboxylate. White solid, m.p.: 133-134 °C, 49% yield. ^{1}H NMR (300 MHz, $CDCl_3$) δ 1.77 (s, 6H, adamantane), 2.08 (s, 9H, adamantane), 3.87 (s, 3H, OCH_3), 7.15-7.18 (m, 1H, Ar), 7.45-7.47 (m, 2H, Ar), 9.94 (s, 1H, CHO). ^{13}C NMR (75 MHz, $CDCl_3$) δ 27.9, 36.4, 38.8, 41.2, 56.1, 110.7, 123.4, 124.8, 135.0, 145.7, 152.0, 175.2, 191.1. Anal. calcd for $C_{19}H_{22}O_4$: C, 72.59; H, 7.05. Found: C, 72.65; H, 7.10.

39eV. 2-ethoxy-4-formylphenyl adamantane-1-carboxylate. Yellow solid, m.p.: 115-117 °C, 58% yield. ^{1}H NMR (300 MHz, $CDCl_3$) δ 1.40 (t, *J* = 7.0 Hz, 3H, $OCH_2\underline{CH_3}$), 1.77 (s, 6H, adamantane), 2.08 (s, 9H, adamantane), 4.09 (q, *J* = 7.0 Hz, 2H, $O\underline{CH_2}CH_3$), 7.16 (d, *J* = 8.4 Hz, 1H, Ar), 7.43-7.46 (m, 2H, Ar), 9.92 (s, 1H, CHO). ^{13}C NMR (75 MHz, $CDCl_3$) δ 14.7, 27.9, 27.9, 36.4, 38.8, 38.8, 41.1, 64.5, 111.5, 123.3, 124.6, 135.0, 145.7, 151.4, 175.1, 191.2. Anal. calcd for $C_{20}H_{24}O_4$: C, 73.15; H, 7.37. Found: C, 73.20; H, 7.32.

55oV. 2-Formyl-6-methoxyphenyl adamantane-1-carboxylate. White solid, m.p. 122-124 °C, 51% yield. ^{1}H NMR (300 MHz, $CDCl_3$) δ 1.78 (bs, 6H, adamantane), 2.11 (bs, 9H, adamantane), 3.83 (s, 3H, OCH_3), 7.17 (dd, 1H, *J* = 8.1, 1.2 Hz, Ar), 7.30 (d, 1H, *J* = 8.1 Hz, Ar), 7.45 (dd, 1H, *J* = 8.4, 1.2 Hz, Ar), 10.13 (s, 1H, CHO). ^{13}C NMR (75 MHz, $CDCl_3$) δ 27.8, 36.4, 38.8, 41.3, 56.3, 117.7, 120.1, 126.4, 129.4, 142.8, 151.7, 175.1, 188.4.

Procedures and characterization data for esters 40V and 41oV

To a solution of the suitable vanilloid (**Van** or ***o*Van**) (1.0 eq) and 3-coumarin carboxylic acid (1.0 eq) in dry dichloromethane (0.2 M), *N,N'*-dicyclohexylcarbodiimide (DCC) (1.5 eq) and DMAP (0.5 eq) were added. The reaction mixture was stirred at room temperature for 16 h. Then, the resulting precipitate of *N,N'*-dicyclohexylurea was removed by vacuum filtration. The crude product was then purified by column flash chromatography on silica gel using a mixture of *n*-hexanes and ethyl acetate as eluent.

40V. 4-formyl-2-methoxyphenyl 2-oxo-2*H*-chromene-3-carboxylate. White solid, m.p.: 266-268 °C, 59% yield. ^{1}H NMR (300 MHz, DMSO-d_6) δ 3.87 (s, 3H, OCH_3), 7.42-7.50 (m, 3H, Ar), 7.62-7.67 (m, 2H, Ar), 7.77-7.82 (m, 1H, Ar), 8.00 (dd, *J* = 7.7, 1.6 Hz, 1H, Ar), 9.11 (s, 1H, Ar), 10.00 (s, 1H, CHO). ^{13}C NMR (75 MHz, DMSO-d_6) δ 56.7, 112.6, 116.0, 116.8, 118.3, 124.1, 131.2, 135.8, 135.9, 144.4, 151.5, 152.0, 155.4, 156.2, 157.1, 160.2, 192.5. Anal. calcd for $C_{18}H_{12}O_6$: C, 66.87; H, 3.73. Found: C, 66.80; H, 3.763.

41oV. 2-formyl-6-methoxyphenyl 2-oxo-2*H*-chromene-3-carboxylate. White solid, m.p. 270-272 °C, 52% yield. ^{1}H NMR (300 MHz, DMSO-d_6) δ 3.84 (s, 3H, OCH_3), 7.42-7.57 (m, 5H, Ar), 7.77-7.82 (m, 1H, Ar), 8.00 (dd, *J* = 7.8, 1.6 Hz, 1H, Ar), 9.14 (s, 1H, Ar), 10.17 (s, 1H, CHO). ^{13}C NMR (75 MHz, DMSO-d_6) δ 56.9, 116.1, 116.8, 118.3, 119.4, 121.5, 125.5, 128.0, 129.4, 131.2, 135.8, 140.6, 151.5, 152.0, 155.4, 160.5, 190.5. Anal. calcd for $C_{18}H_{12}O_6$: C, 66.87; H, 3.73. Found: C, 66.93; H, 3.70.

Procedure and characterization data for thiosemicarbazone ***42oV***

To **20oV** (1 eq) in absolute ethanol (0.17 M), thiosemicarbazide (1.1 eq) and a catalytic amount of acetic acid (two drops) were added. Then, the reaction mixture was stirred at reflux for 4 h. Once the reaction was completed, the mixture was poured into ice water and extracted three times with dichloromethane. The combined organic layers were dried over anhydrous sodium sulfate, filtered, and concentrated under reduced pressure to afford the desired product.

42oV. 2-(3-methoxy-2-((3-nitrobenzyl)oxy)benzylidene)hydrazine-1-carbothioamide. Pale yellow crystals, m.p.: 206-208 °C, 90% yield. ^{1}H NMR (300 MHz, DMSO-d_6) δ 3.83 (s, 3H, CH_3O), 5.05 (s, 2H, -CH_2-Ph), 7.08-7.09 (m, 2H, =CH and Ar) (s, 1H, =CH), 7.66-7.71 (m, 2H, Ar), 7.92 (d, 2H, *J* = 6.6, Ar), 8.19 (d, 2H, *J* = 8.1, Ar), 8.29-8.34 (s, 2H, NH_2), 11.43 (s, 1H, NH). ^{13}C NMR (75 MHz, DMSO-d_6) δ 56.3, 74.0, 114.4, 118.0, 123.1, 123.4, 125.0, 128.5, 130.4, 135.2, 138.4, 139.7, 146.6, 148.2, 152.9, 178.3. Anal. Calcd. for $C_{16}H_{16}N_4O_4S$: C, 53.32; H, 4.48; N, 15.55. Found: C, 53.26; H, 4.42; N, 15.59.

Procedure and characterization data for benzylidene hydrazinylthiazoles 43-46oV

To **42oV** (1.0 eq) in absolute ethanol (0.15 M), the appropriate phenacyl bromide derivative (1.1 eq) was added, and the reaction mixture was stirred at reflux for 3-9 h. Then, the reaction mixture was concentrated under reduced pressure and cooled to 0-5 °C. The formed precipitate was filtered under vacuum filtration and washed with petroleum ether and diethyl ether (20 mL).

43oV. 2-(2-(3-methoxy-2-((3-nitrobenzyl)oxy)benzylidene)hydrazineyl)-4-(4-nitrophenyl)thiazole. Reddish orange solid, m.p.: 253-255 °C, 87% yield. ^{1}H NMR (300 MHz, DMSO-d_6) δ 3.85 (s, 3H, CH_3O), 5.11 (s, 2H, -CH_2-Ph), 7.07-7.16 (m, 2H,=CH and Ar), 7.33-7.40 (m,-1H, Ar), 7.65-7.71 (m, 2H, Ar), 7.86-7.90 (m, 1H, Ar), 8.03-8.08 (m, 2H, Ar) , 8.16-8.30 (m, 5H, Ar), 12.21 (s, 1H, NH). ^{13}C NMR (75 MHz, DMSO-d_6) δ 56.3, 74,0.0, 123.2, 123.4, 124.6, 125.3, 126.8, 128.5, 130.4, 135.3, 137.8, 139.7, 141.1, 145.9, 146.7, 148.2, 153.1, 168.9. Anal. Calcd. for $C_{24}H_{19}N_5O_6S$: C, 57.02; H, 3.79; N, 13.85. Found: C, 57.09; H, 3.74; N, 13.80.

44oV. 2-(2-(3-methoxy-2-((3-nitrobenzyl)oxy)benzylidene)hydrazineyl)-4-phenylthiazole. Pale yellow solid, m.p.: 193-195 °C, 98% yield. ^{1}H NMR (300 MHz, DMSO-d_6) δ 3.86 (s, 3H, CH_3O), 5.11 (s, 2H, -CH_2-Ph), 7.08–7.18 (m, 2H, Ar and =CH), 7.25-7.30 (m, 2H, Ar), 7.36–7.41 (m, 3H, Ar), 7.67-7.73 (m, 1H, Ar), 7.81-7.84 (m, 2H, Ar), 7.89-7.93 (m, 1H, Ar), 8.20-8.24 (m, 2H, Ar), 8.31-8.32 (m, 1H, Ar), 12.15 (s, 1H, NH). ^{13}C NMR (75 MHz, DMSO-d_6) δ 56.7, 73.9, 119.1, 119.4, 123.1, 123.5, 125.3, , 129.0, 129.8, 130.5, 135.4, 135.6, 139.8, 143.6, 148.2, 157.1, 166.5. Anal. Calcd. for $C_{24}H_{20}N_4O_4S$: C, 62.60; H, 4.38; N, 12.17. Found: C, 62.69; H, 4.43; N, 12.21.

45oV. 2-(2-(3-methoxy-2-((3-nitrobenzyl)oxy)benzylidene)hydrazineyl)-4-(3-nitrophenyl)thiazole. Yellow solid, m.p.: 190-192 °C, 98% yield. ^{1}H NMR (300 MHz, DMSO-d_6) δ 3.86 (s, 3H, CH_3O), 5.11 (s, 2H, -CH_2-Ph), 7.09-7.18 (m, 2H, =CH and Ar), 7.36-7.39 (m, 1H, Ar), 7.64-7.73 (m, 3H, Ar), 7.89-7.92 (m, 1H, Ar), 8.11-8.14 (m, 1H, Ar), 8.20-8.32 (m, 4H, Ar), 8.63-8.64 (m, 1H, Ar), 12.29 (s, 1H, NH).^{13}C NMR (75 MHz, DMSO-d_6) δ 56.3, 73.9, 113.6, 123.1, 123.5, 124.5, 129.3, 130.3, 131.4, 135.2, 135.4, 136.1, 139.7, 139.8, 152.9, 153.1, 157.2, 164.7. Anal. Calcd. for $C_{24}H_{19}N_5O_6S$: C, 57.02; H, 3.79; N, 13.85. Found C, 56.92; H, 3.83; N, 13.89.

46oV. 4-(2-(2-(3-methoxy-2-((3-nitrobenzyl)oxy)benzylidene)hydrazineyl)thiazol-4-yl)phenol. Orange solid, m.p.: 170-172 °C, 97% yield. ^{1}H NMR (300 MHz, DMSO-d_6) δ 3.85 (s, 3H, CH_3O), 5.10 (s, 2H, -CH_2-Ph), 6.73-6.77 (m, 2H, Ar), 7.02 (s, 1H, =CH), 7.07-7.17 (m, 2H, Ar), 7.35-7.38 (m, 1H, Ar), 7.59-7.72 (m, 3H, Ar), 7.89-7.94 (d, *J* = 8.0 Hz, 1H, Ar), 8.19-8.23 (m, 2H, Ar), 8.30-8.34 (m, 1H, Ar). ^{13}C NMR (75 MHz, DMSO-d_6) δ 56.3, 74.0, 115.8, 117.0, 123.2, 123.4, 125.3, 127.5, 130.4, 135.3, 139.7, 148.2, 153.1, 168.3. Anal. Calcd. for $C_{24}H_{20}N_4O_5S$: C, 60.50; H, 4.23; N, 11.76. Found: C, 60.58; H, 4.28; N, 11.79.

In silico ADME predictions

cmpd1V

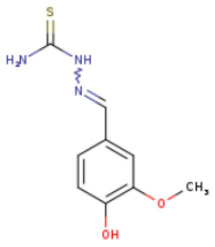

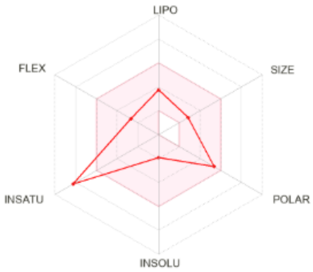

SMILES COc1cc(C=NNC(=S)N)ccc1O

Physicochemical Properties	
Formula	C9H11N3O2S
Molecular weight	225.27 g/mol
Num. heavy atoms	15
Num. arom. heavy atoms	6
Fraction Csp3	0.11
Num. rotatable bonds	4
Num. H-bond acceptors	3
Num. H-bond donors	3
Molar Refractivity	61.72
TPSA	111.96 Å²

Lipophilicity	
Log $P_{o/w}$ (iLOGP)	1.89
Log $P_{o/w}$ (XLOGP3)	1.03
Log $P_{o/w}$ (WLOGP)	0.57
Log $P_{o/w}$ (MLOGP)	0.28
Log $P_{o/w}$ (SILICOS-IT)	1.57
Consensus Log $P_{o/w}$	1.07

Water Solubility	
Log *S* (ESOL)	-1.92
Solubility	2.72e+00 mg/ml ; 1.21e-02 mol/l
Class	Very soluble
Log *S* (Ali)	-2.97
Solubility	2.41e-01 mg/ml ; 1.07e-03 mol/l
Class	Soluble
Log *S* (SILICOS-IT)	-1.90
Solubility	2.86e+00 mg/ml ; 1.27e-02 mol/l
Class	Soluble

Pharmacokinetics	
GI absorption	High
BBB permeant	No
P-gp substrate	No
CYP1A2 inhibitor	No
CYP2C19 inhibitor	No
CYP2C9 inhibitor	No
CYP2D6 inhibitor	No
CYP3A4 inhibitor	No
Log K_p (skin permeation)	-6.94 cm/s

Druglikeness	
Lipinski	Yes; 0 violation
Ghose	Yes
Veber	Yes
Egan	Yes
Muegge	Yes
Bioavailability Score	0.55

Medicinal Chemistry	
PAINS	1 alert: hzone_phenol_B
Brenk	2 alerts: imine_1, thiocarbonyl_group
Leadlikeness	No; 1 violation: MW<250
Synthetic accessibility	2.24

cmpd2oV

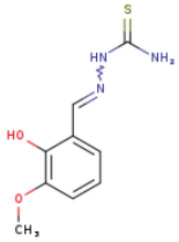

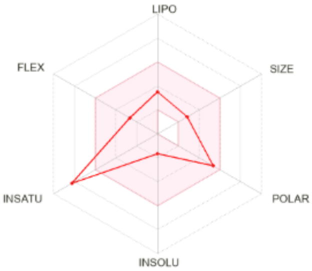

SMILES COc1cccc(c1O)C=NNC(=S)N

Physicochemical Properties	
Formula	C9H11N3O2S
Molecular weight	225.27 g/mol
Num. heavy atoms	15
Num. arom. heavy atoms	6
Fraction Csp3	0.11
Num. rotatable bonds	4
Num. H-bond acceptors	3
Num. H-bond donors	3
Molar Refractivity	61.72
TPSA	111.96 Å²

Lipophilicity	
Log $P_{o/w}$ (iLOGP)	1.99
Log $P_{o/w}$ (XLOGP3)	0.63
Log $P_{o/w}$ (WLOGP)	0.57
Log $P_{o/w}$ (MLOGP)	0.28
Log $P_{o/w}$ (SILICOS-IT)	1.57
Consensus Log $P_{o/w}$	1.01

Water Solubility	
Log *S* (ESOL)	-1.67
Solubility	4.87e+00 mg/ml ; 2.16e-02 mol/l
Class	Very soluble
Log *S* (Ali)	-2.56
Solubility	6.26e-01 mg/ml ; 2.78e-03 mol/l
Class	Soluble
Log *S* (SILICOS-IT)	-1.90
Solubility	2.86e+00 mg/ml ; 1.27e-02 mol/l
Class	Soluble

Pharmacokinetics	
GI absorption	High
BBB permeant	No
P-gp substrate	No
CYP1A2 inhibitor	No
CYP2C19 inhibitor	No
CYP2C9 inhibitor	No
CYP2D6 inhibitor	No
CYP3A4 inhibitor	No
Log K_p (skin permeation)	-7.23 cm/s

Druglikeness	
Lipinski	Yes; 0 violation
Ghose	Yes
Veber	Yes
Egan	Yes
Muegge	Yes
Bioavailability Score	0.55

Medicinal Chemistry	
PAINS	1 alert: hzone_phenol_A
Brenk	2 alerts: imine_1, thiocarbonyl_group
Leadlikeness	No; 1 violation: MW<250
Synthetic accessibility	2.36

cmpd4eV

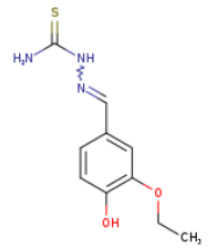

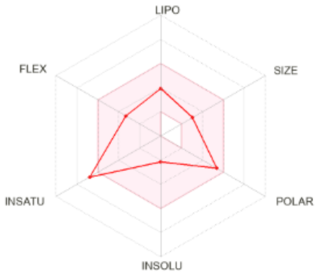

SMILES CCOc1cc(C=NNC(=S)N)ccc1O

Physicochemical Properties	
Formula	C10H13N3O2S
Molecular weight	239.29 g/mol
Num. heavy atoms	16
Num. arom. heavy atoms	6
Fraction Csp3	0.20
Num. rotatable bonds	5
Num. H-bond acceptors	3
Num. H-bond donors	3
Molar Refractivity	66.52
TPSA	111.96 Å²

Lipophilicity	
Log $P_{o/w}$ (iLOGP)	2.13
Log $P_{o/w}$ (XLOGP3)	1.40
Log $P_{o/w}$ (WLOGP)	0.96
Log $P_{o/w}$ (MLOGP)	0.58
Log $P_{o/w}$ (SILICOS-IT)	1.94
Consensus Log $P_{o/w}$	1.40

Water Solubility	
Log *S* (ESOL)	-2.15
Solubility	1.68e+00 mg/ml ; 7.03e-03 mol/l
Class	Soluble
Log *S* (Ali)	-3.36
Solubility	1.06e-01 mg/ml ; 4.41e-04 mol/l
Class	Soluble
Log *S* (SILICOS-IT)	-2.30
Solubility	1.20e+00 mg/ml ; 5.01e-03 mol/l
Class	Soluble

Pharmacokinetics	
GI absorption	High
BBB permeant	No
P-gp substrate	No
CYP1A2 inhibitor	No
CYP2C19 inhibitor	No
CYP2C9 inhibitor	No
CYP2D6 inhibitor	No
CYP3A4 inhibitor	No
Log K_p (skin permeation)	-6.77 cm/s

Druglikeness	
Lipinski	Yes; 0 violation
Ghose	Yes
Veber	Yes
Egan	Yes
Muegge	Yes
Bioavailability Score	0.55

Medicinal Chemistry	
PAINS	1 alert: hzone_phenol_B
Brenk	2 alerts: imine_1, thiocarbonyl_group
Leadlikeness	No; 1 violation: MW<250
Synthetic accessibility	2.38

cmpd16V

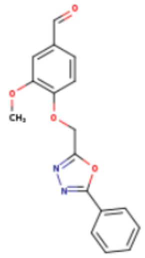

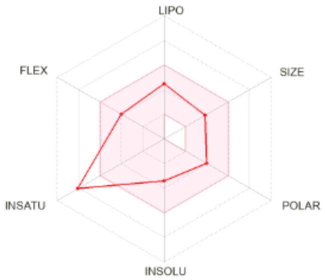

SMILES COc1cc(C=O)ccc1OCc1nnc(o1)c1ccccc1

Physicochemical Properties	
Formula	C17H14N2O4
Molecular weight	310.30 g/mol
Num. heavy atoms	23
Num. arom. heavy atoms	17
Fraction Csp3	0.12
Num. rotatable bonds	6
Num. H-bond acceptors	6
Num. H-bond donors	0
Molar Refractivity	82.59
TPSA	74.45 Å²

Lipophilicity	
Log $P_{o/w}$ (iLOGP)	2.73
Log $P_{o/w}$ (XLOGP3)	2.35
Log $P_{o/w}$ (WLOGP)	2.98
Log $P_{o/w}$ (MLOGP)	1.15
Log $P_{o/w}$ (SILICOS-IT)	3.48
Consensus Log $P_{o/w}$	2.54

Water Solubility	
Log *S* (ESOL)	-3.40
Solubility	1.25e-01 mg/ml ; 4.02e-04 mol/l
Class	Soluble
Log *S* (Ali)	-3.55
Solubility	8.68e-02 mg/ml ; 2.80e-04 mol/l
Class	Soluble
Log *S* (SILICOS-IT)	-6.11
Solubility	2.43e-04 mg/ml ; 7.83e-07 mol/l
Class	Poorly soluble

Pharmacokinetics	
GI absorption	High
BBB permeant	Yes
P-gp substrate	No
CYP1A2 inhibitor	Yes
CYP2C19 inhibitor	Yes
CYP2C9 inhibitor	Yes
CYP2D6 inhibitor	Yes
CYP3A4 inhibitor	Yes
Log K_p (skin permeation)	-6.52 cm/s

Druglikeness	
Lipinski	Yes; 0 violation
Ghose	Yes
Veber	Yes
Egan	Yes
Muegge	Yes
Bioavailability Score	0.55

Medicinal Chemistry	
PAINS	0 alert
Brenk	1 alert: aldehyde
Leadlikeness	Yes
Synthetic accessibility	3.01

NMR spectra of the newly synthesized compounds

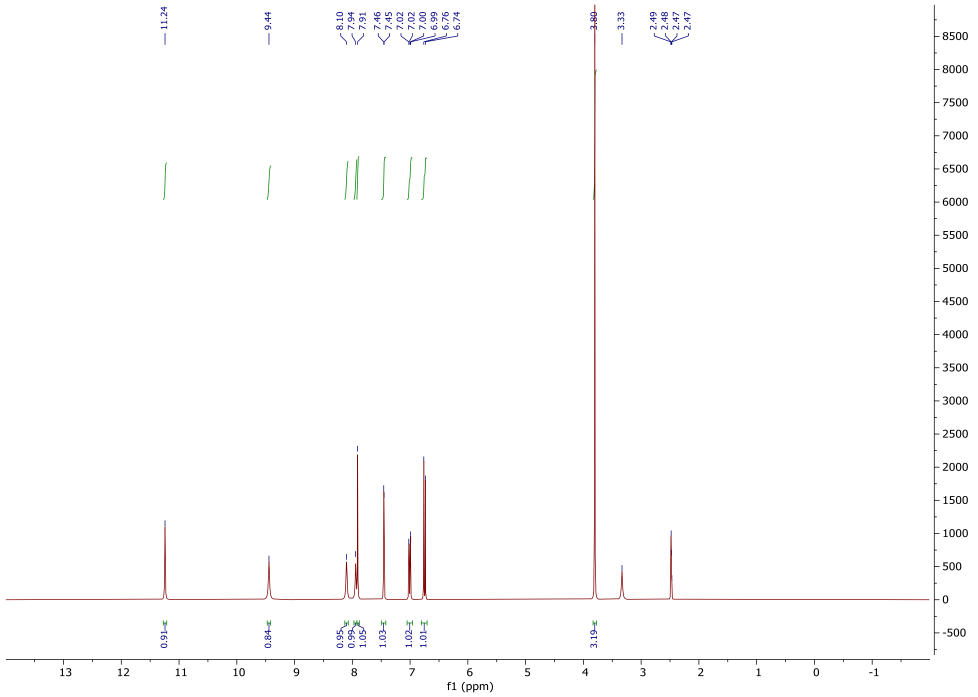

Figure S1. ^{1}H NMR of compound **1V**.

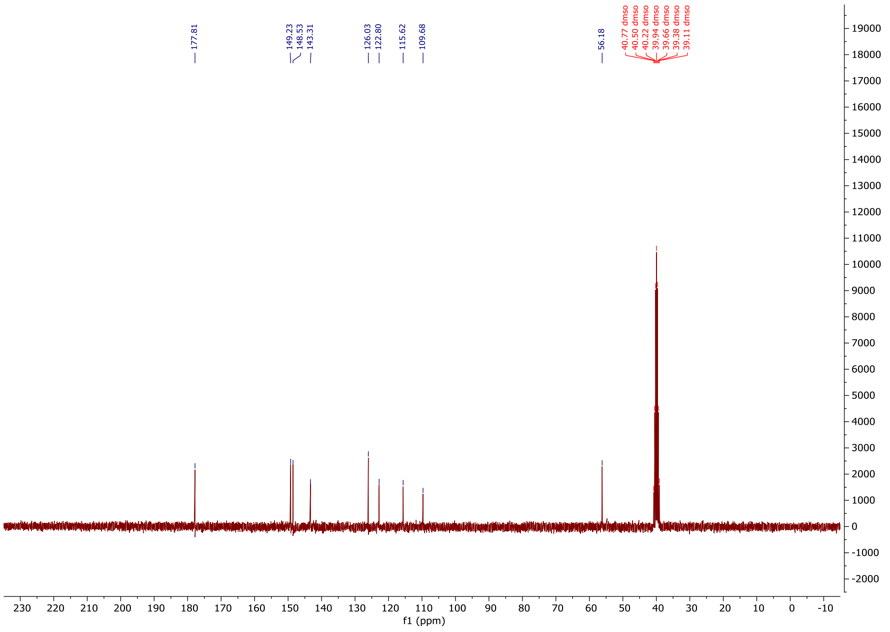

Figure S2. ^{13}C NMR of compound **1V**.

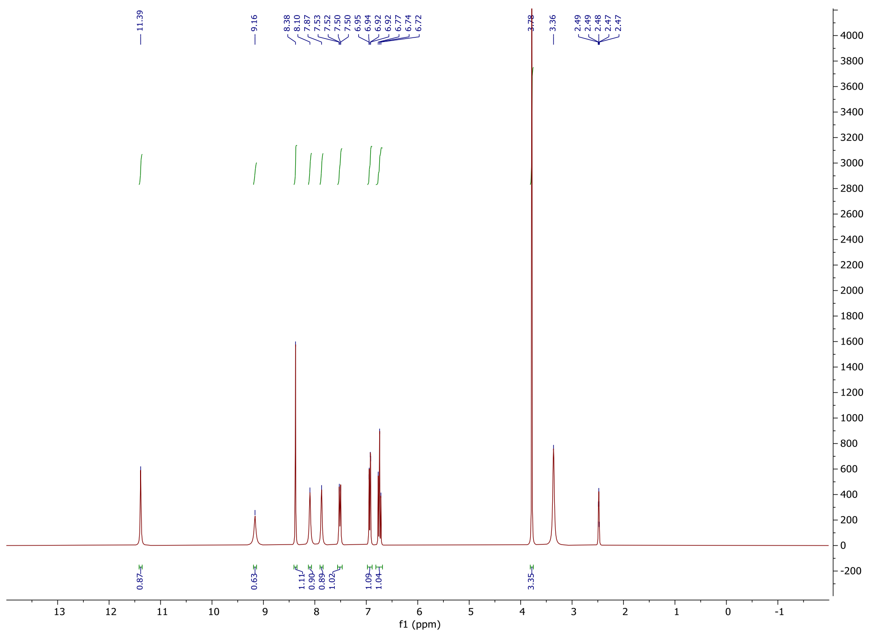

Figure S3. ^{1}H NMR of compound **2oV**.

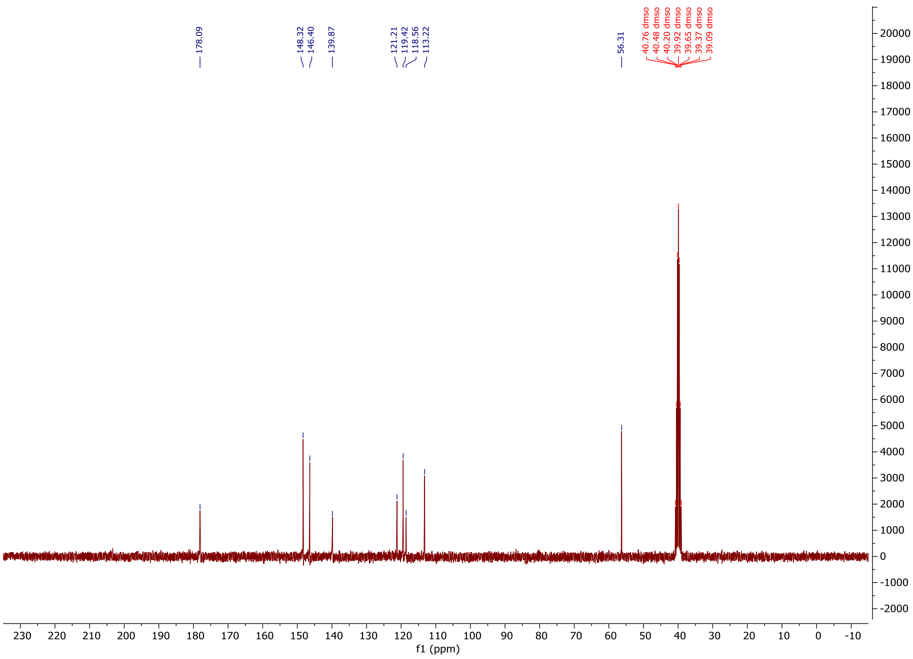

Figure S4. ^{13}C NMR of compound **2oV**.

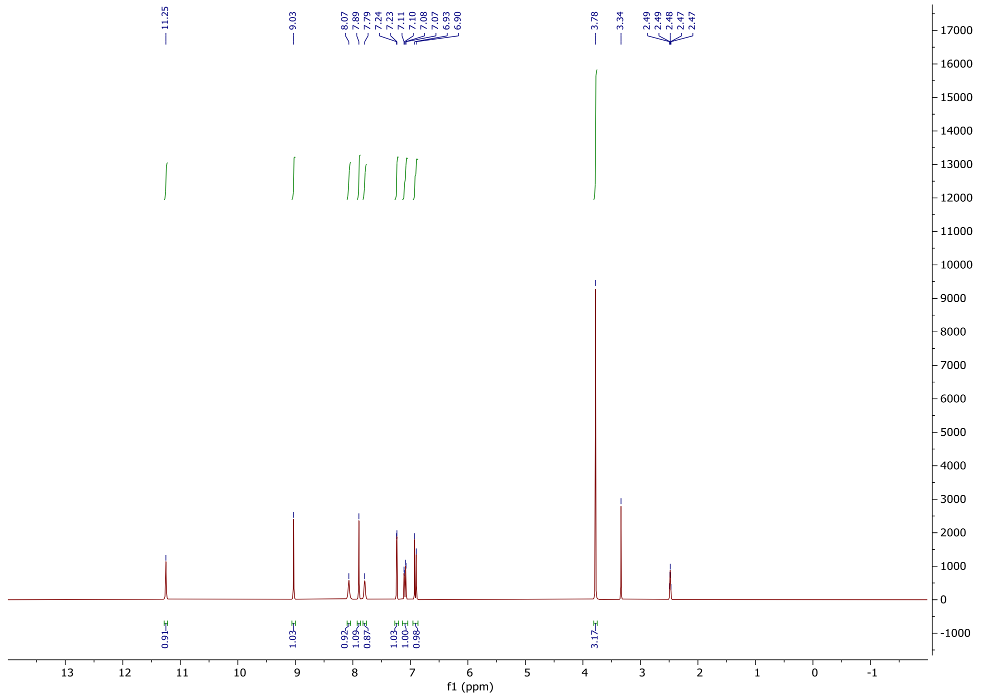

Figure S5. ^{1}H NMR of compound **3iV**.

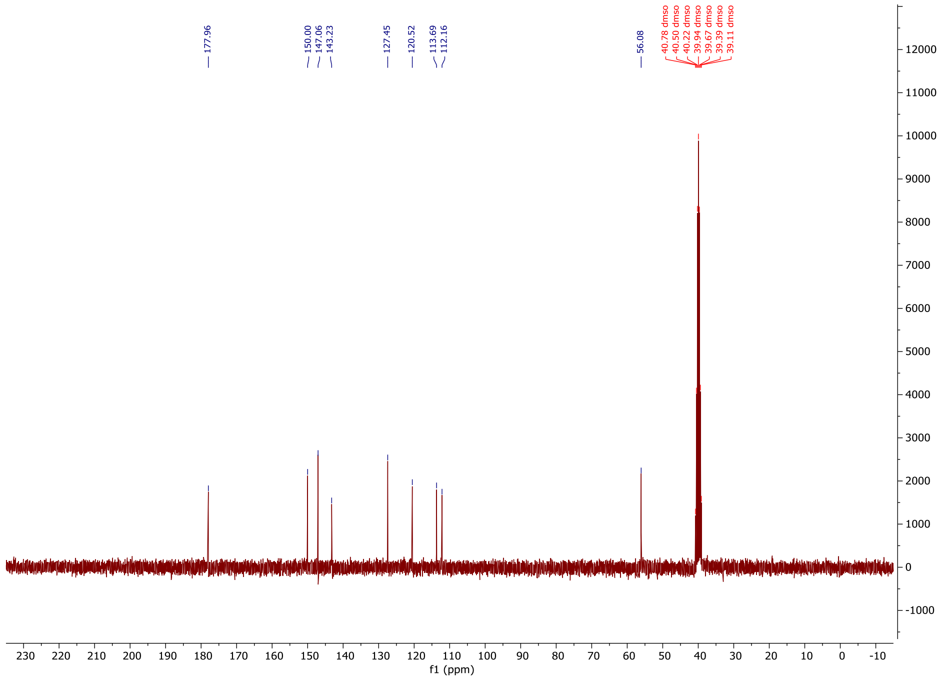

Figure S6. ^{13}C NMR of compound **3iV**.

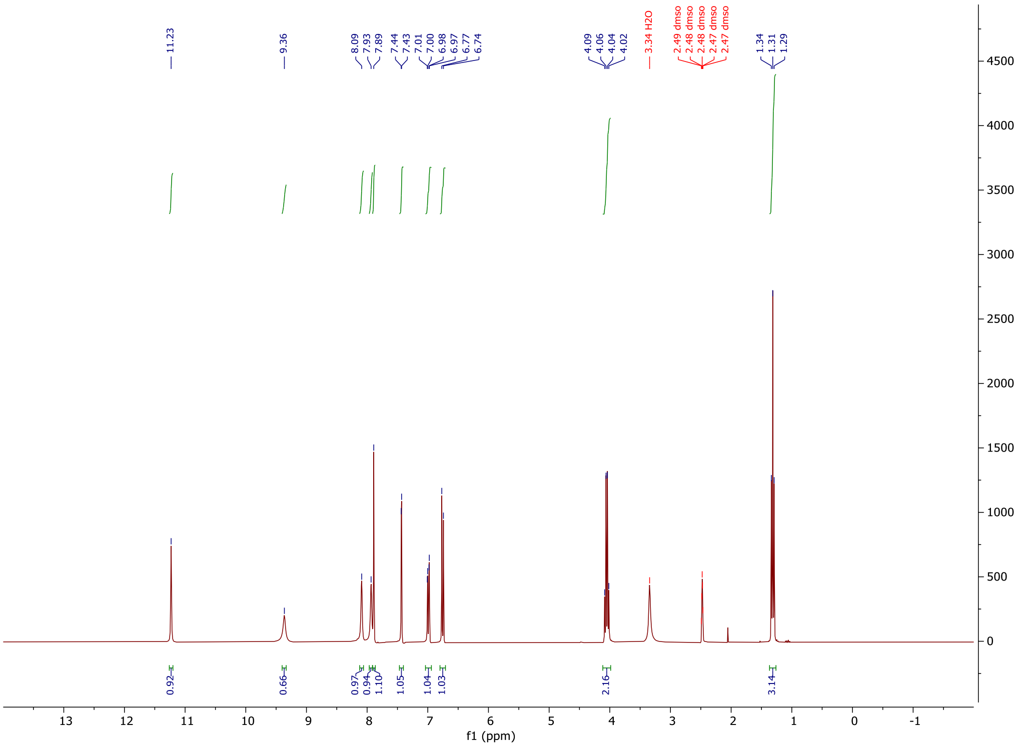

Figure S7. ^{1}H NMR of compound **4eV**.

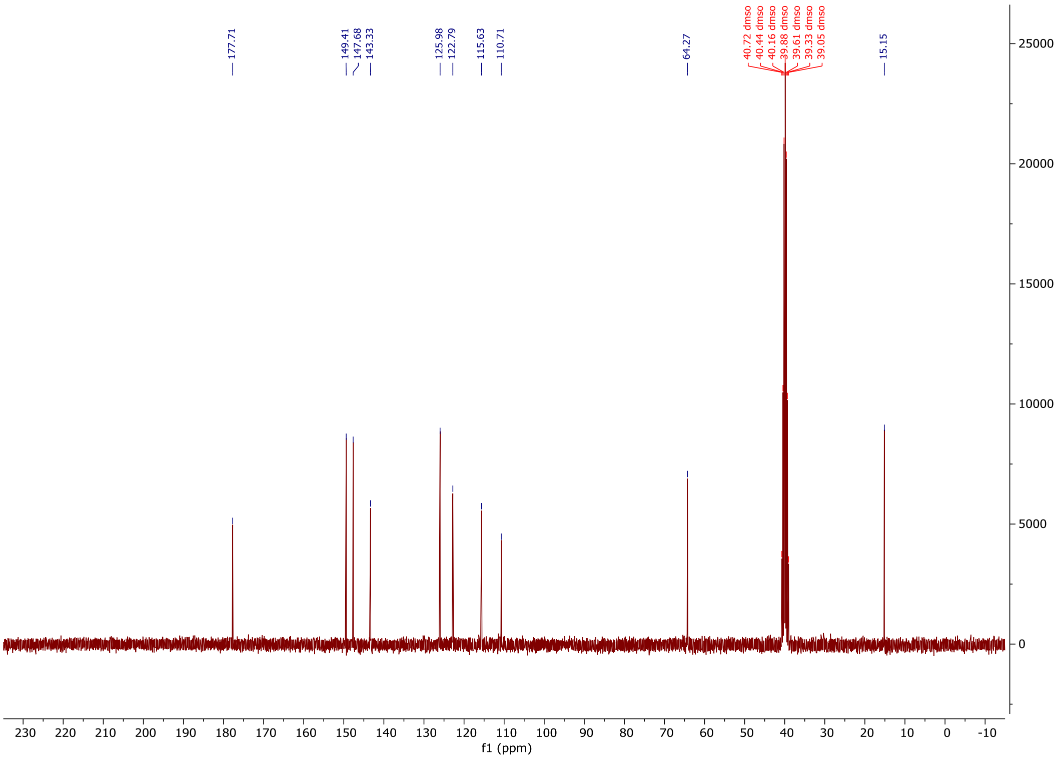

Figure S8. ^{13}C NMR of compound **4eV**.

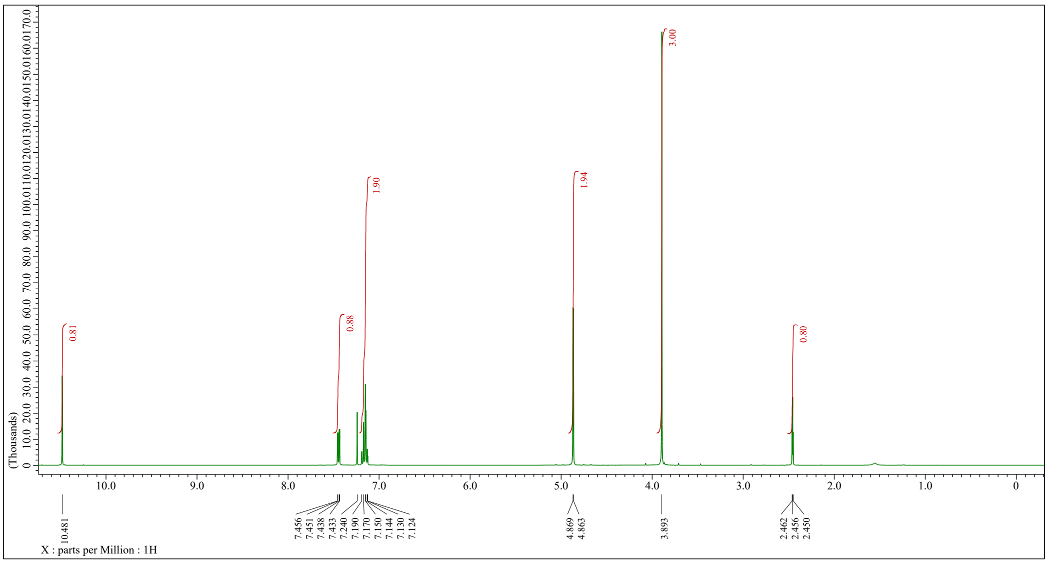

Figure S9. ^{1}H NMR of compound **6oV**.

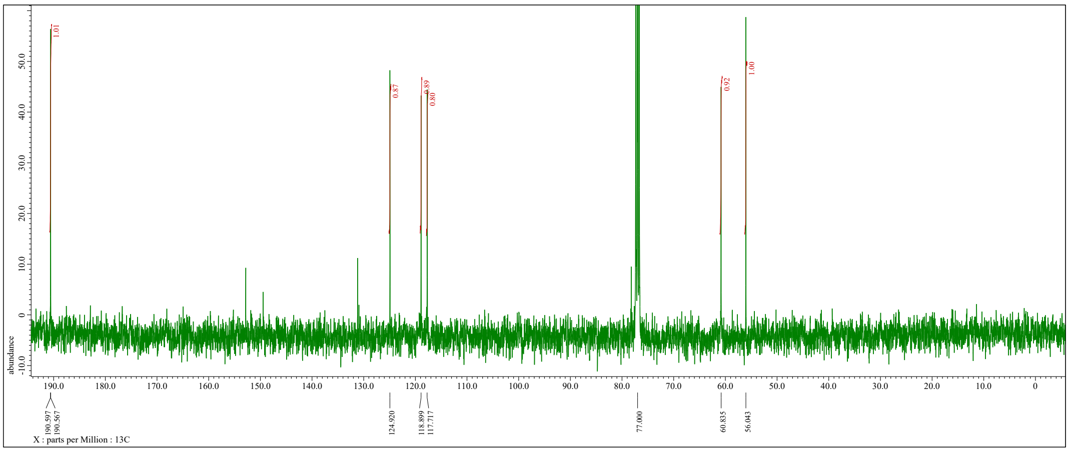

Figure S10. ^{13}C NMR of compound **6oV**.

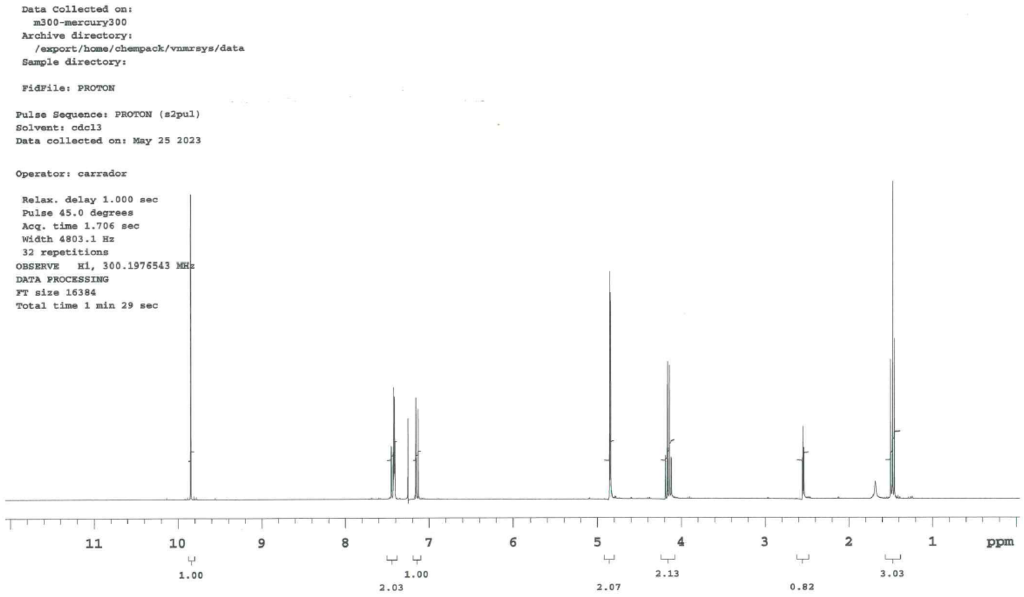

Figure S11. ^{1}H NMR of compound **7eV**.

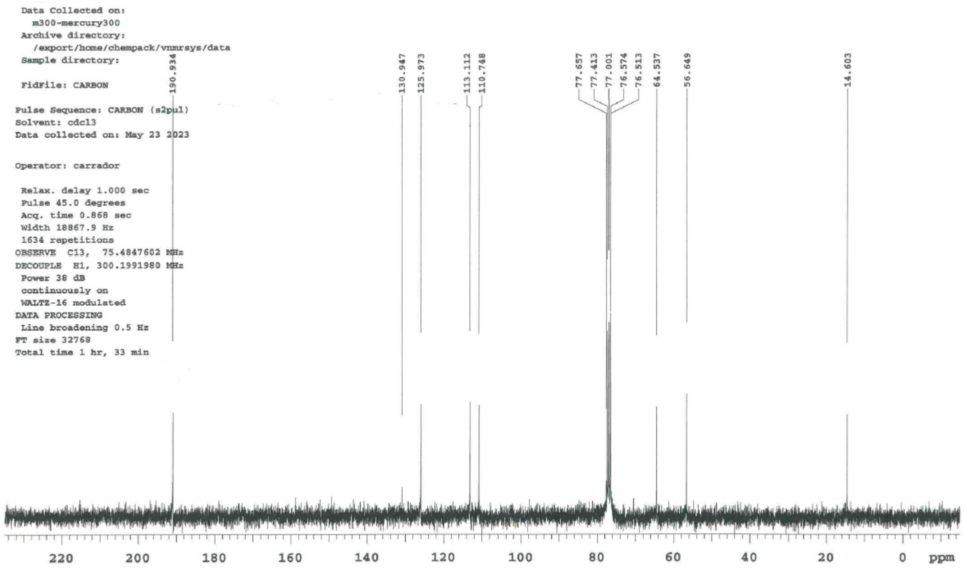

Figure S12. ^{13}C NMR of compound **7eV**.

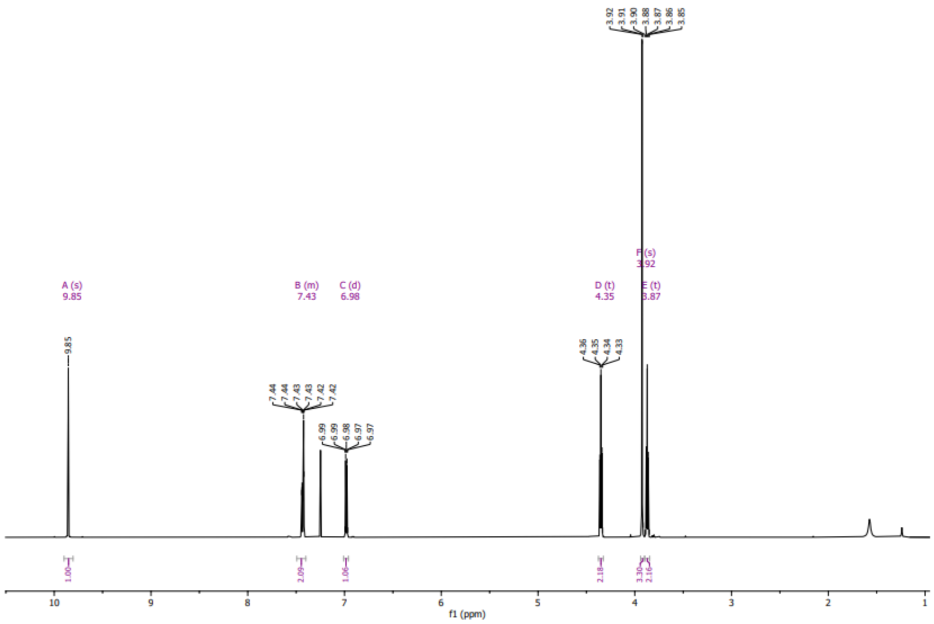

Figure S13. ^{1}H NMR of compound **8V**.

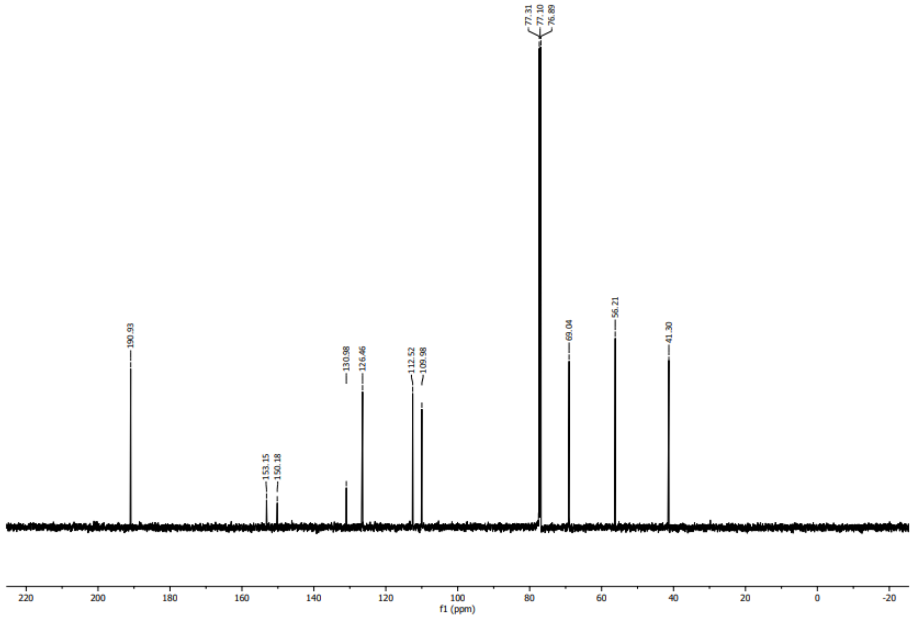

Figure S14. ^{13}C NMR of compound **8V**.

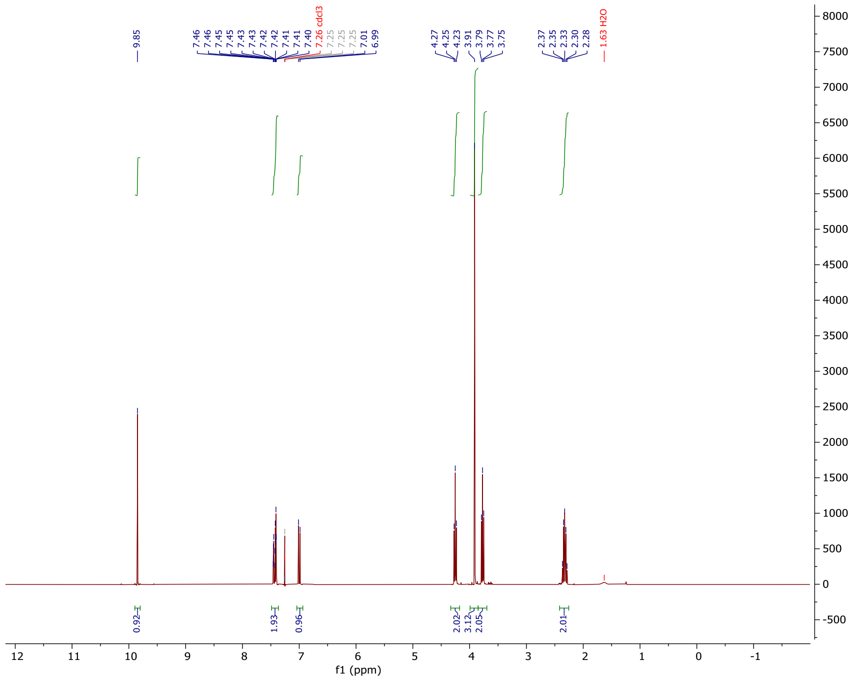

Figure S15. ^{1}H NMR of compound **9V**.

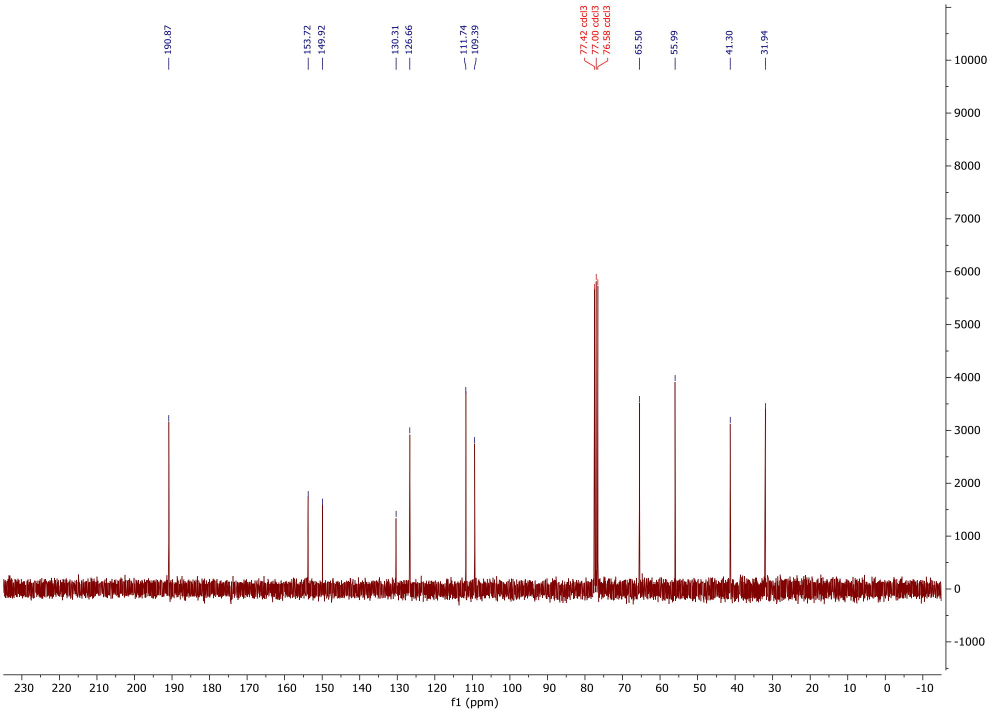

Figure S16. ^{13}C NMR of compound **9V**.

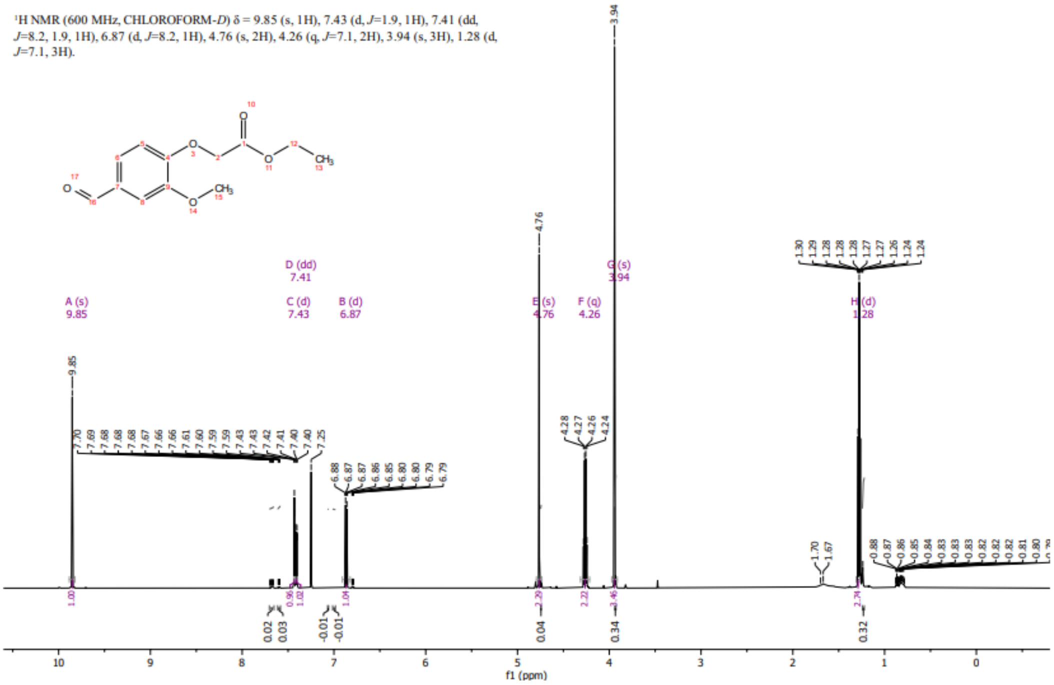

Figure S17. ^{1}H NMR of compound **10V**.

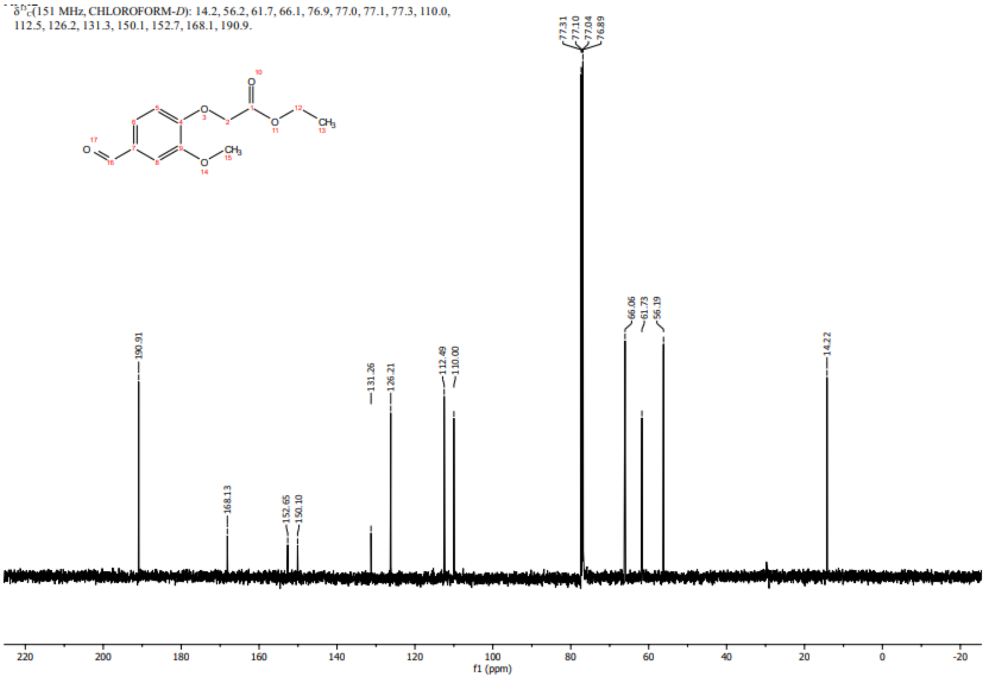

Figure S18. ^{13}C NMR of compound **10V**.

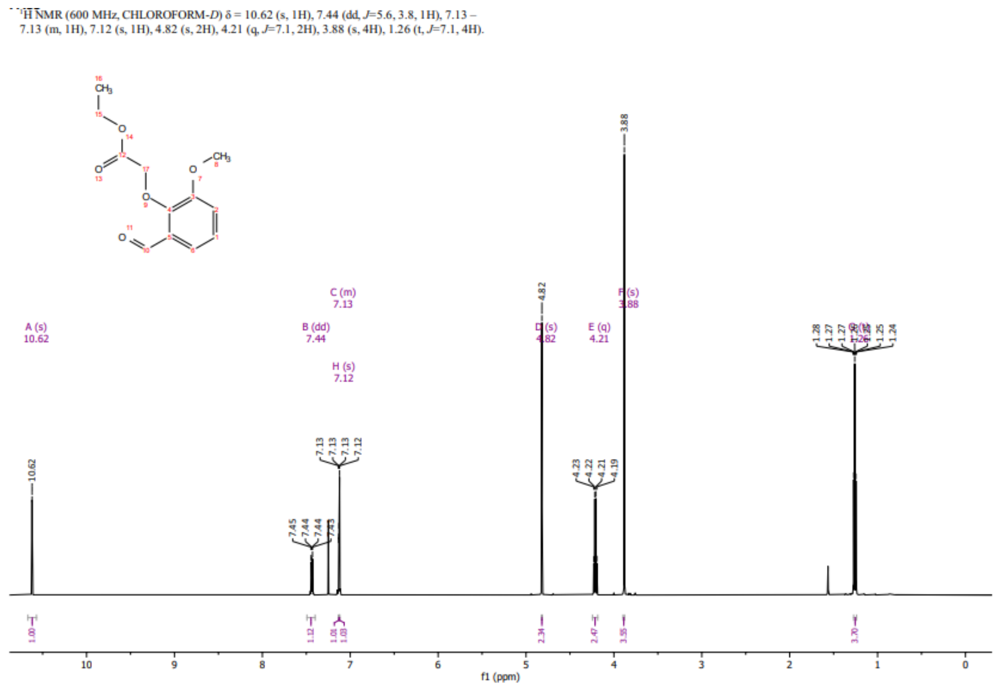

Figure S19. ^{1}H NMR of compound **11oV**.

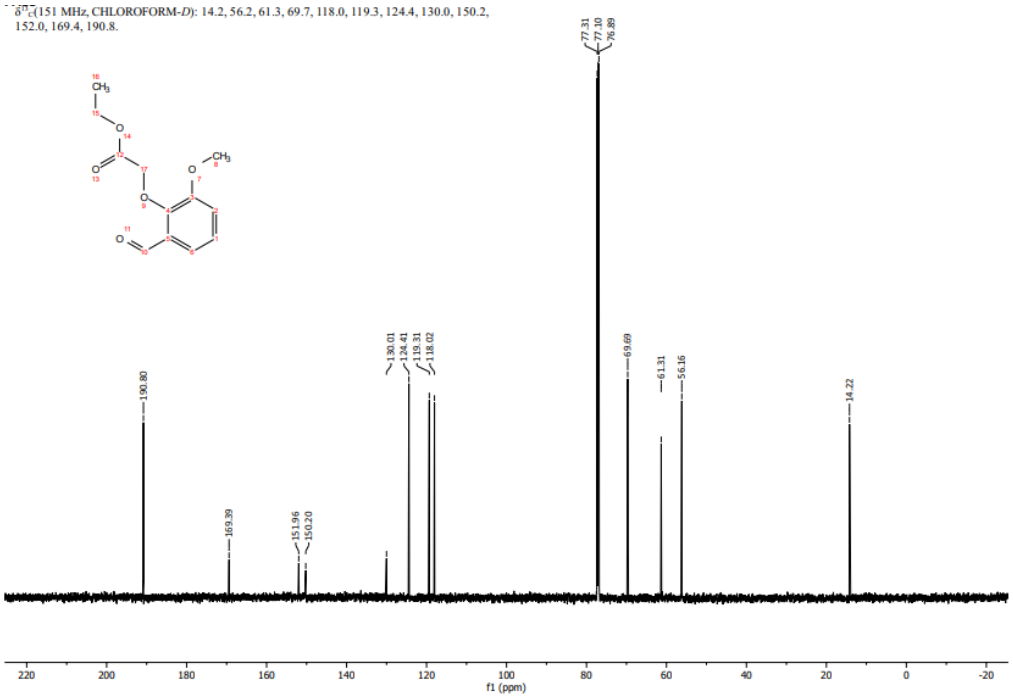

Figure S20. ^{13}C NMR of compound **11oV**.

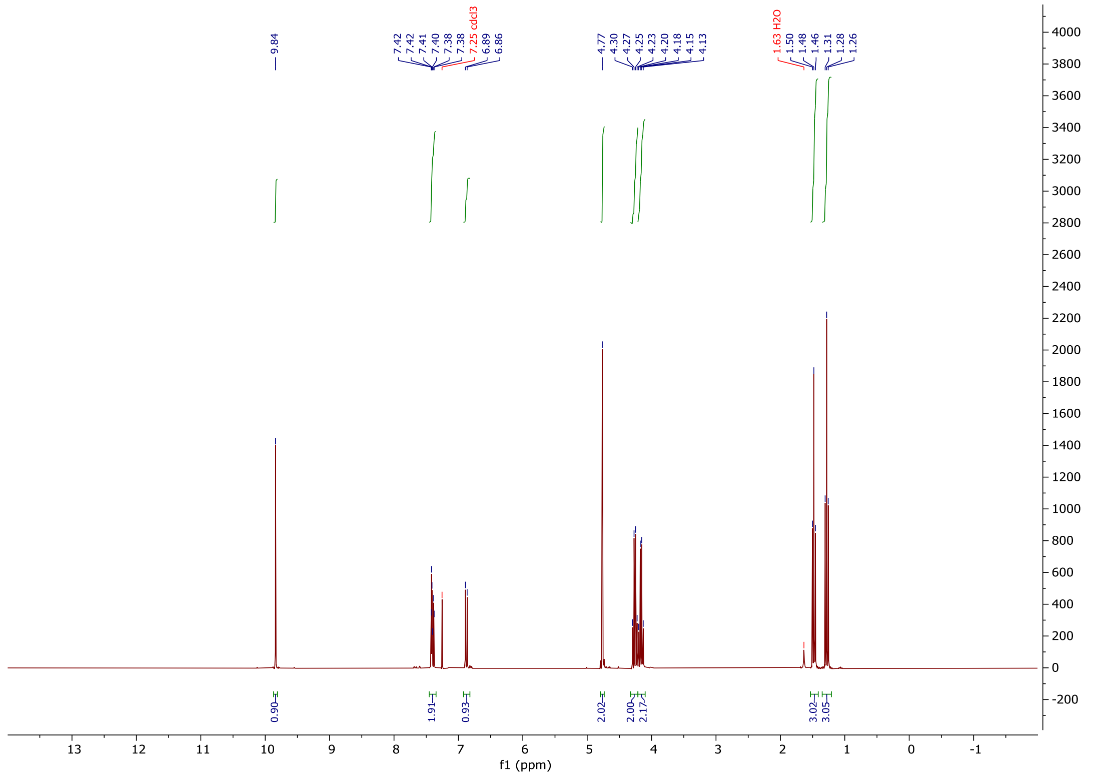

Figure S21. ^{1}H NMR of compound **12eV**.

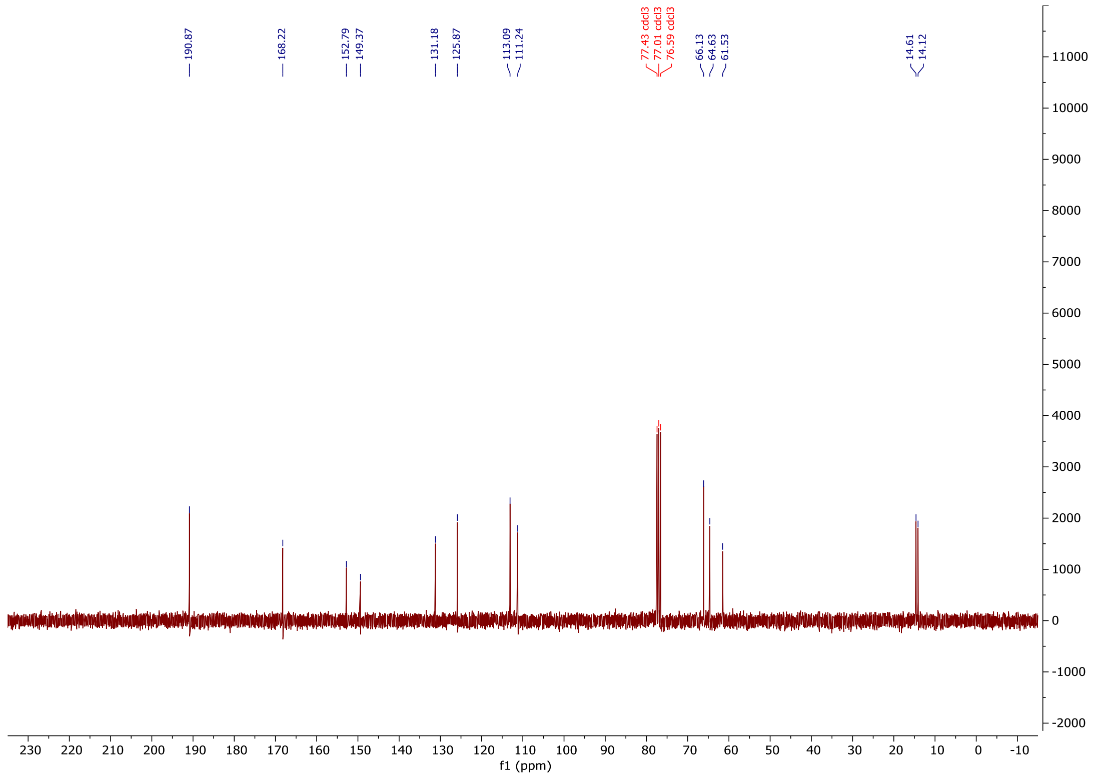

Figure S22. ^{13}C NMR of compound **12eV**.

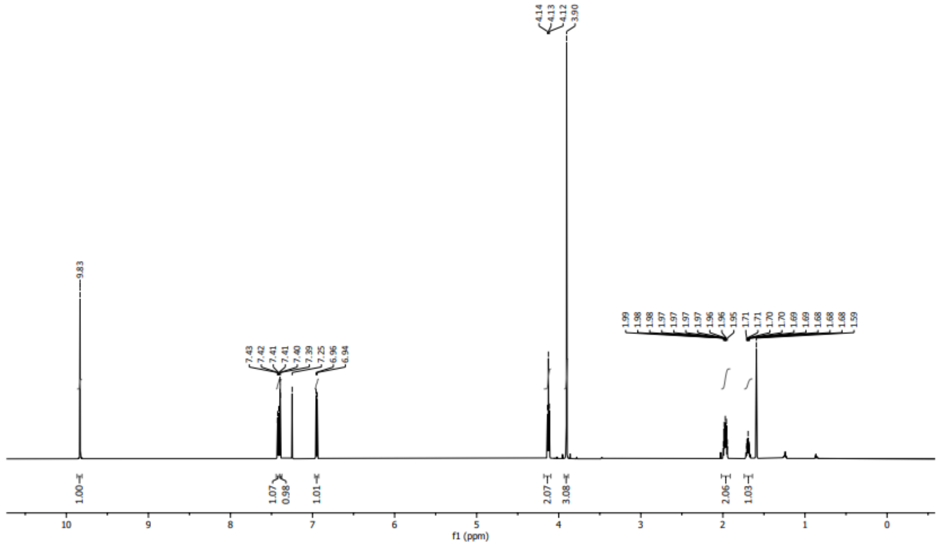

Figure S23. ^{1}H NMR of compound **13V**.

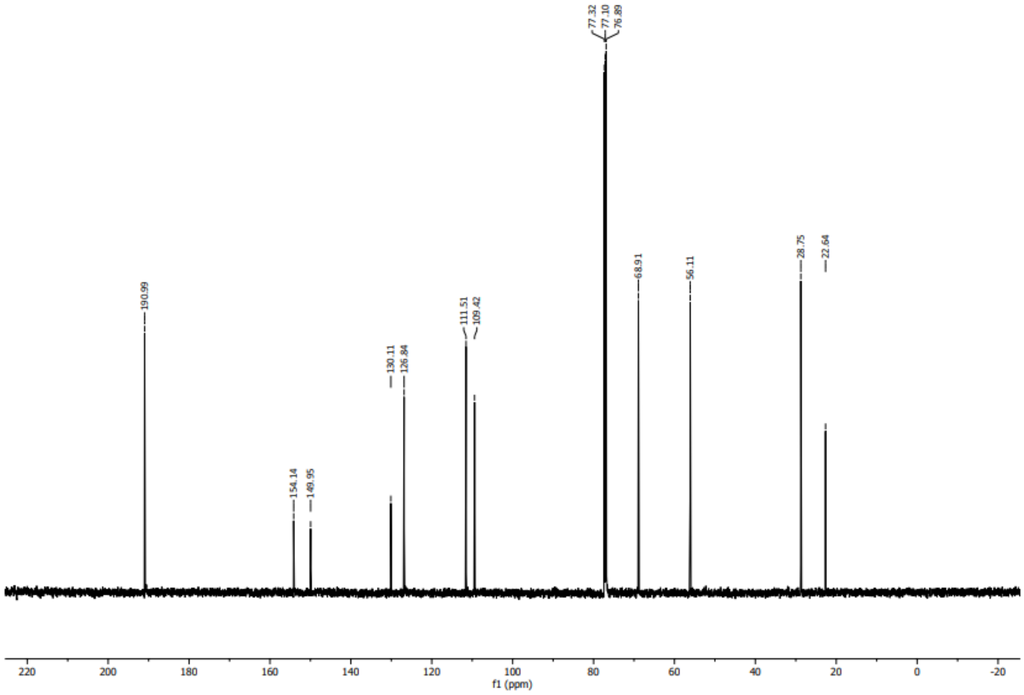

Figure S24. ^{13}C NMR of compound **13V**.

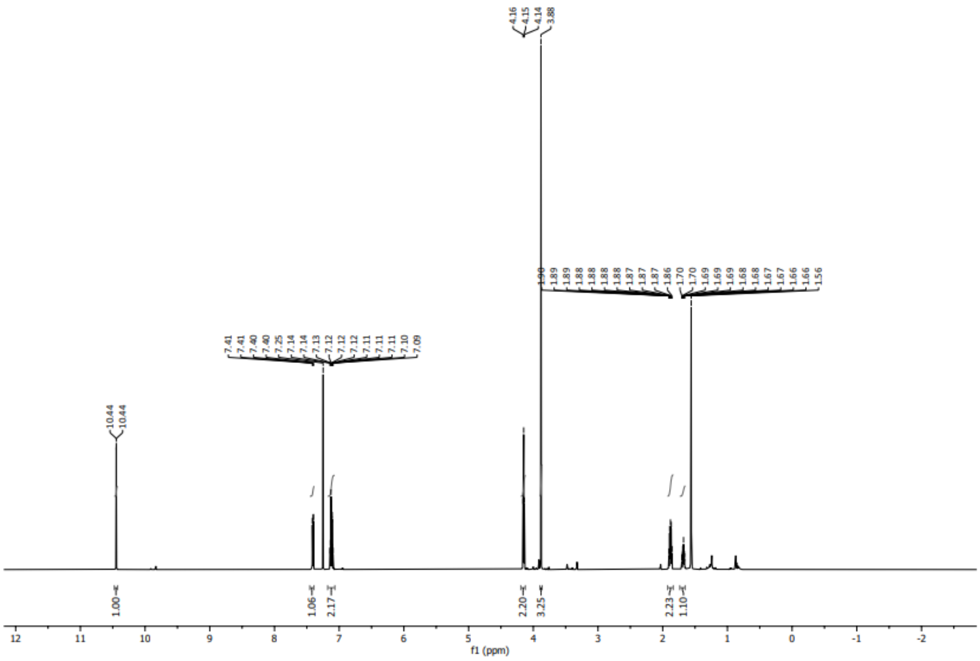

Figure S25. ^{1}H NMR of compound **14oV**.

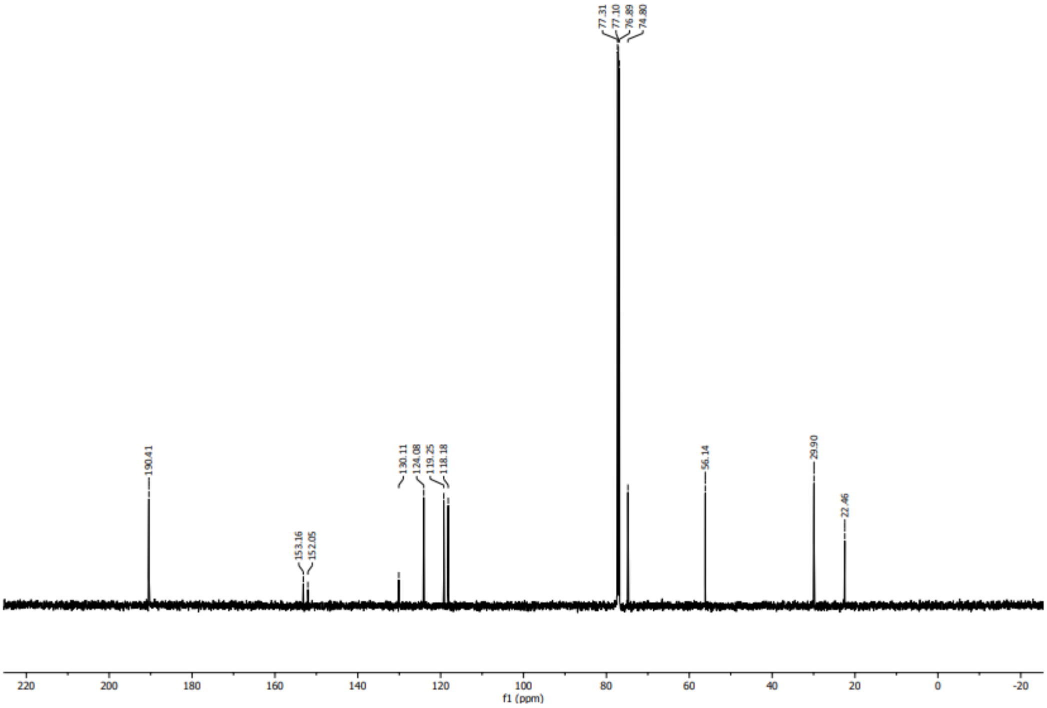

Figure S26. ^{13}C NMR of compound **14oV**.

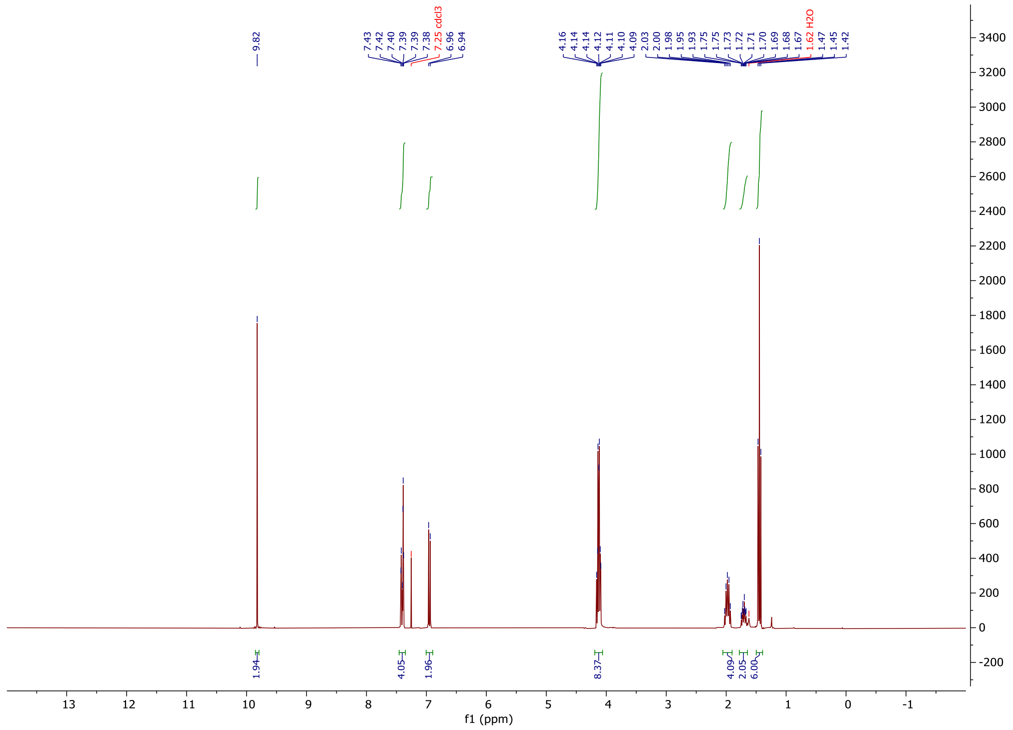

Figure S27. ¹H NMR of compound **15eV**.

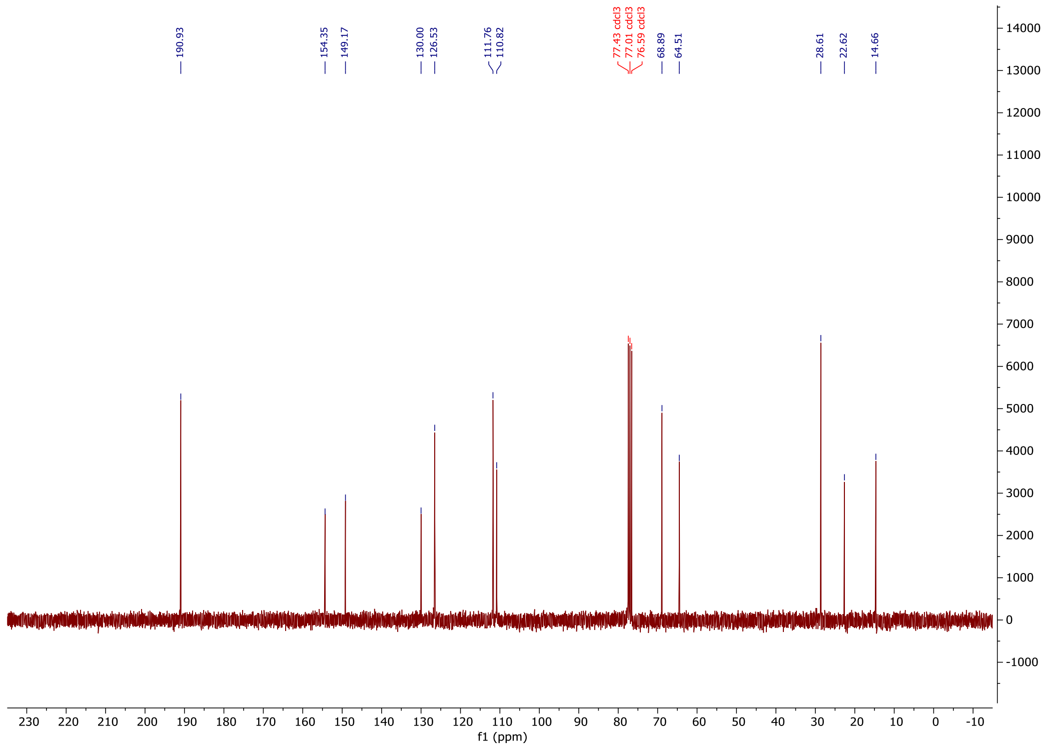

Figure S28. ¹³C NMR of compound **15eV**.

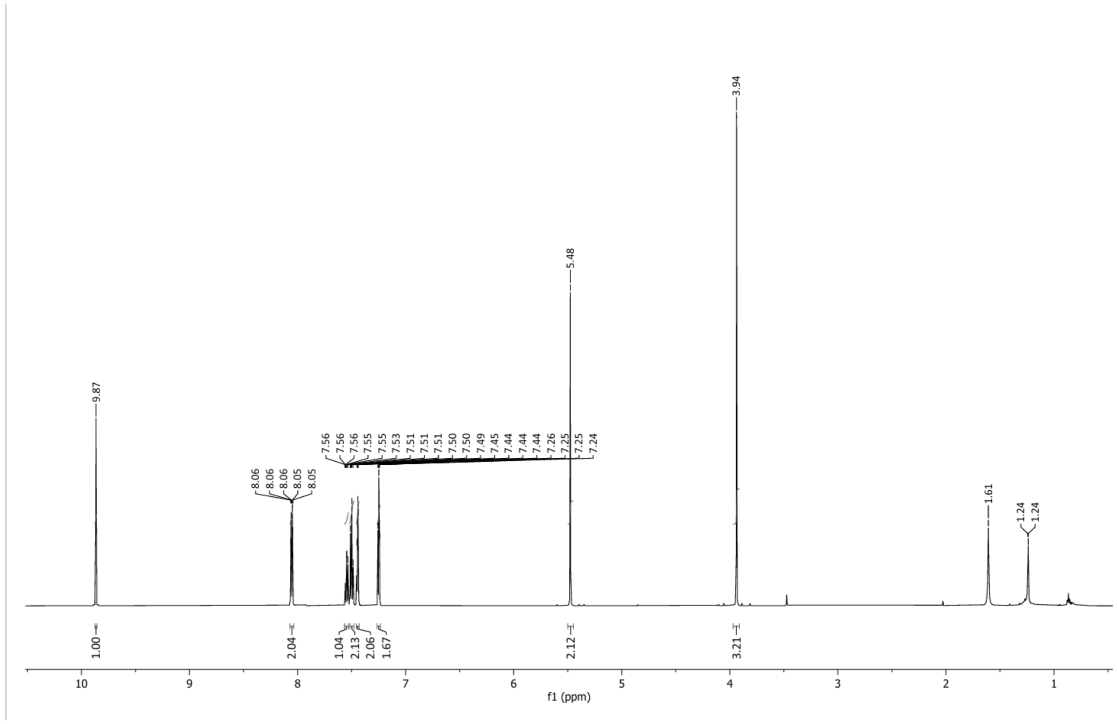

Figure S29. ^{1}H NMR of compound **16V**.

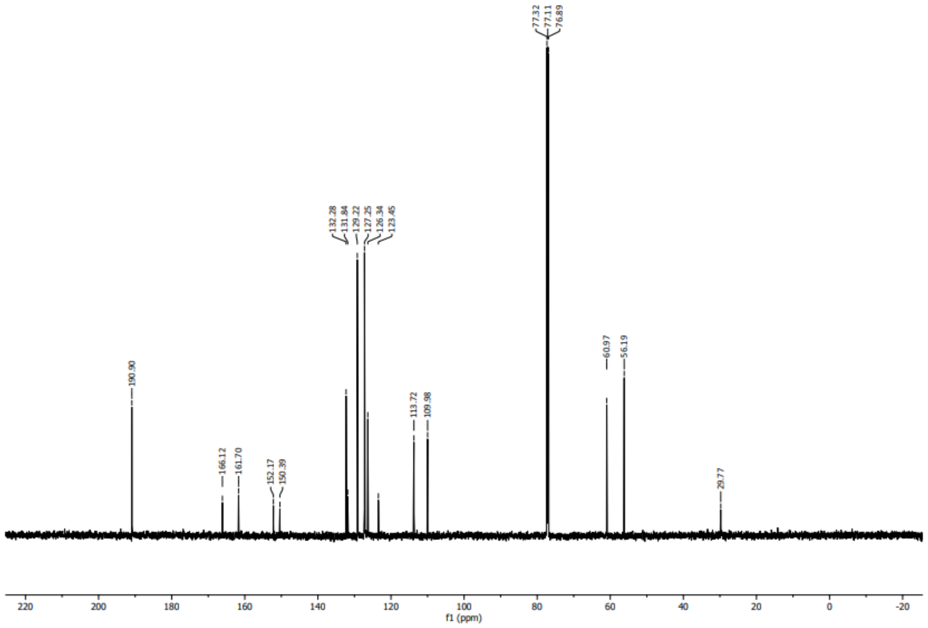

Figure S30. ^{13}C NMR of compound **16V**.

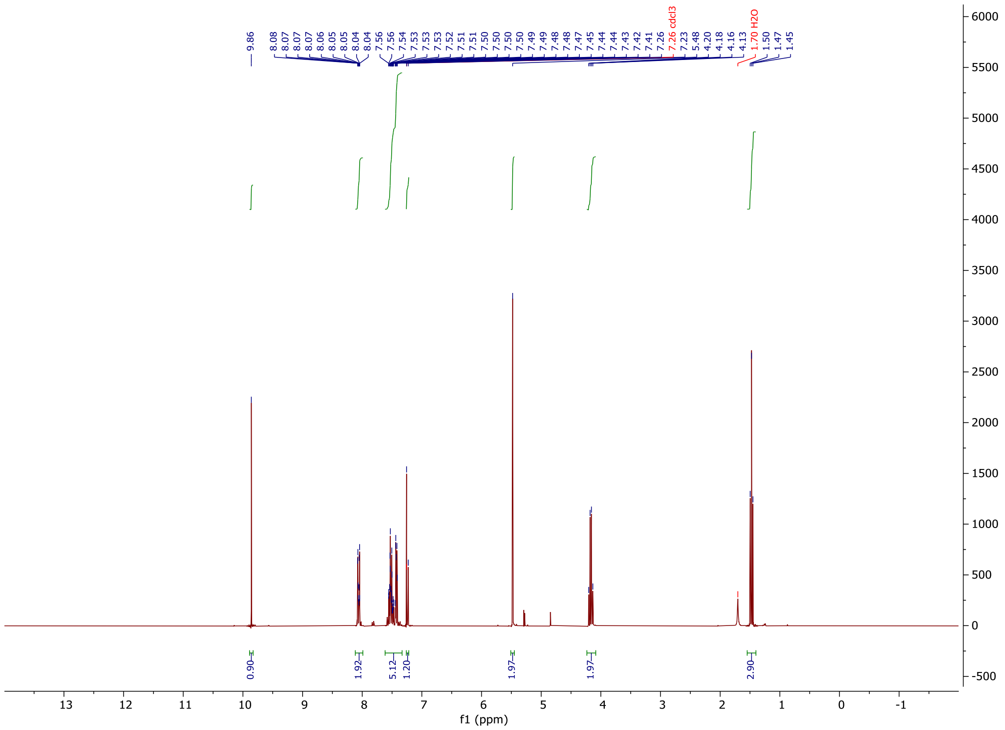

Figure S31. ¹H NMR of compound **17eV**.

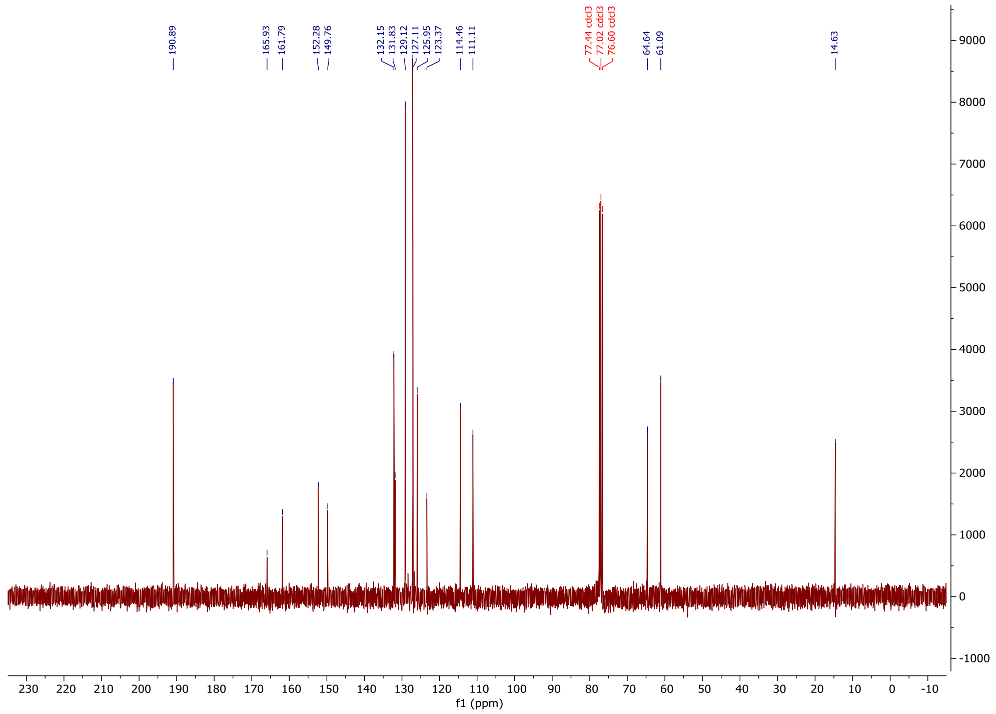

Figure S32. ¹³C NMR of compound **17eV**.

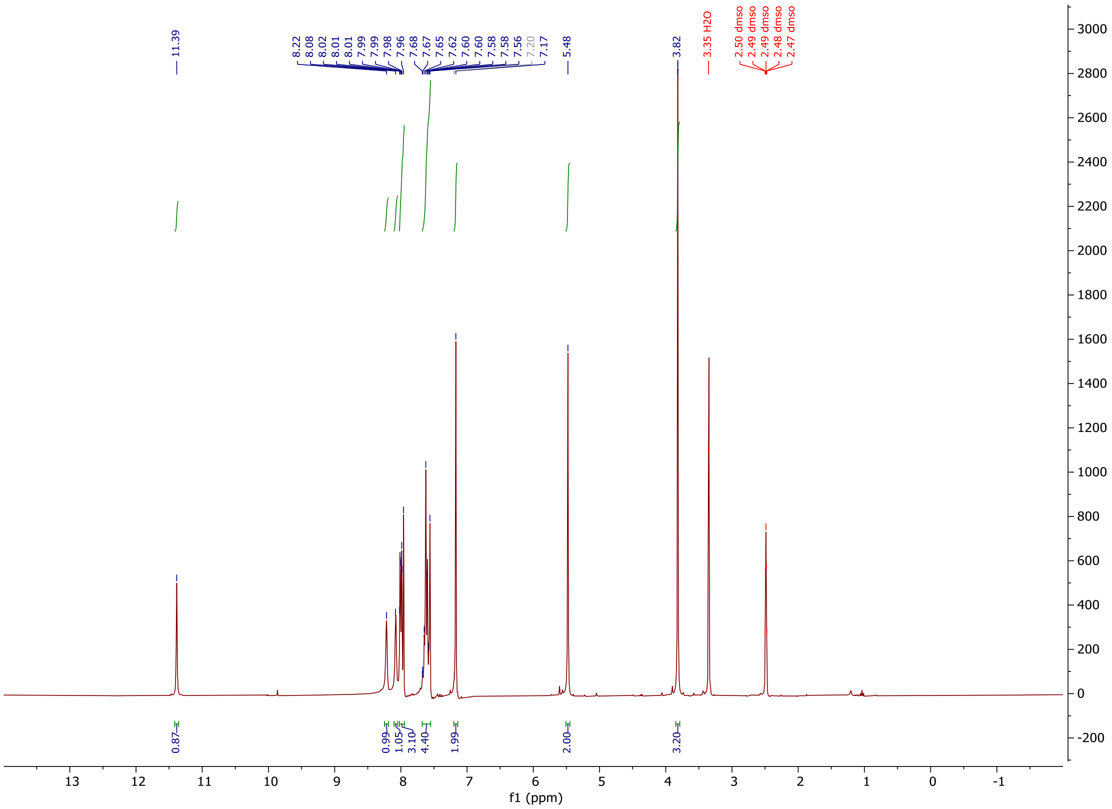

Figure S33. ^{1}H NMR of compound **18V**.

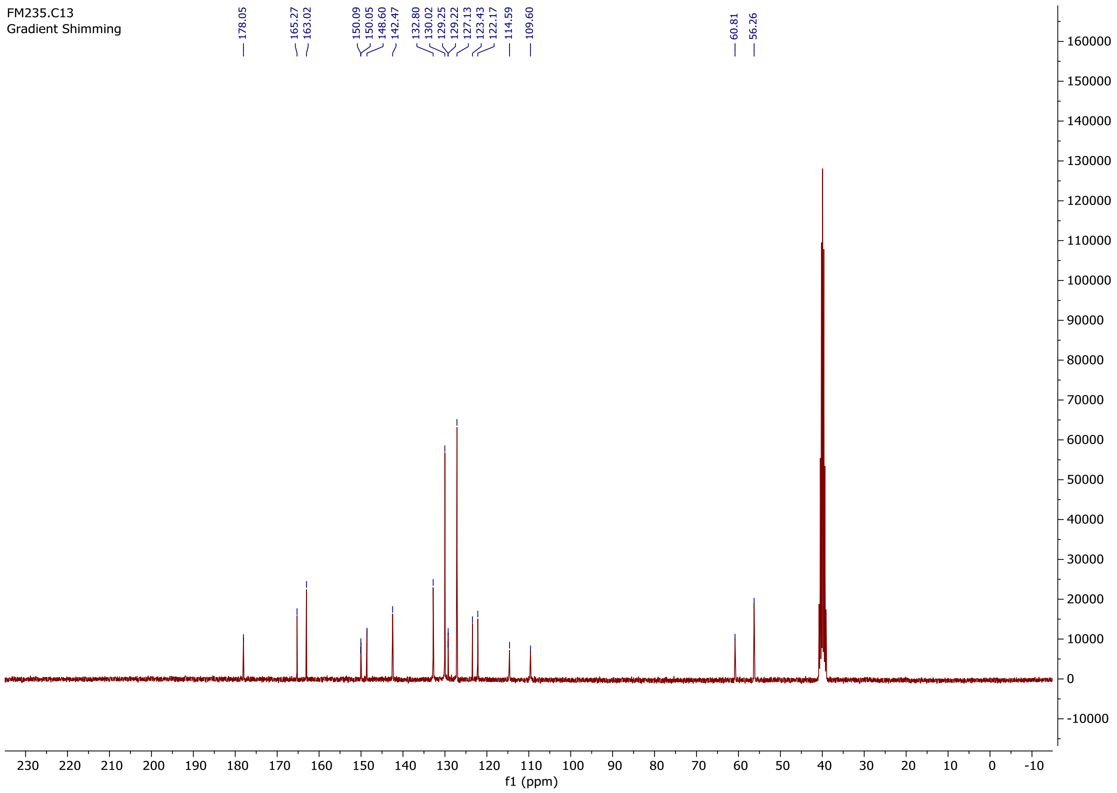

Figure S34. ^{13}C NMR of compound **18V**.

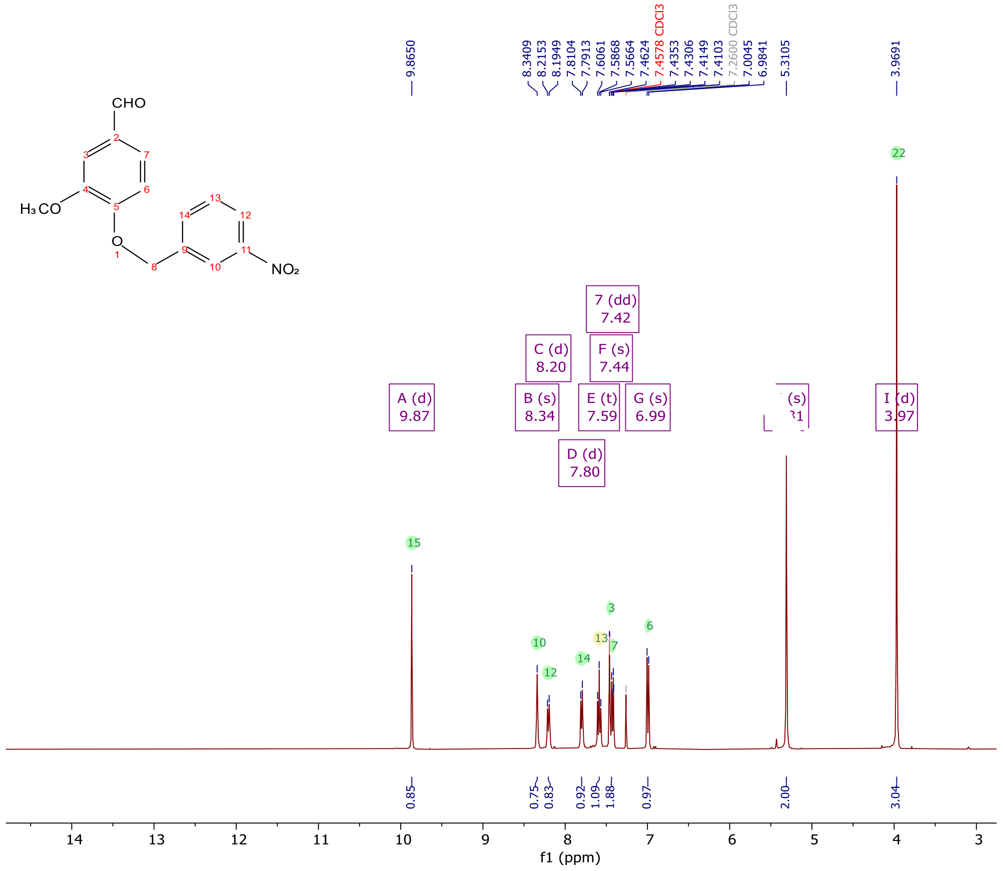

Figure S35. ^{1}H NMR of compound **19V**.

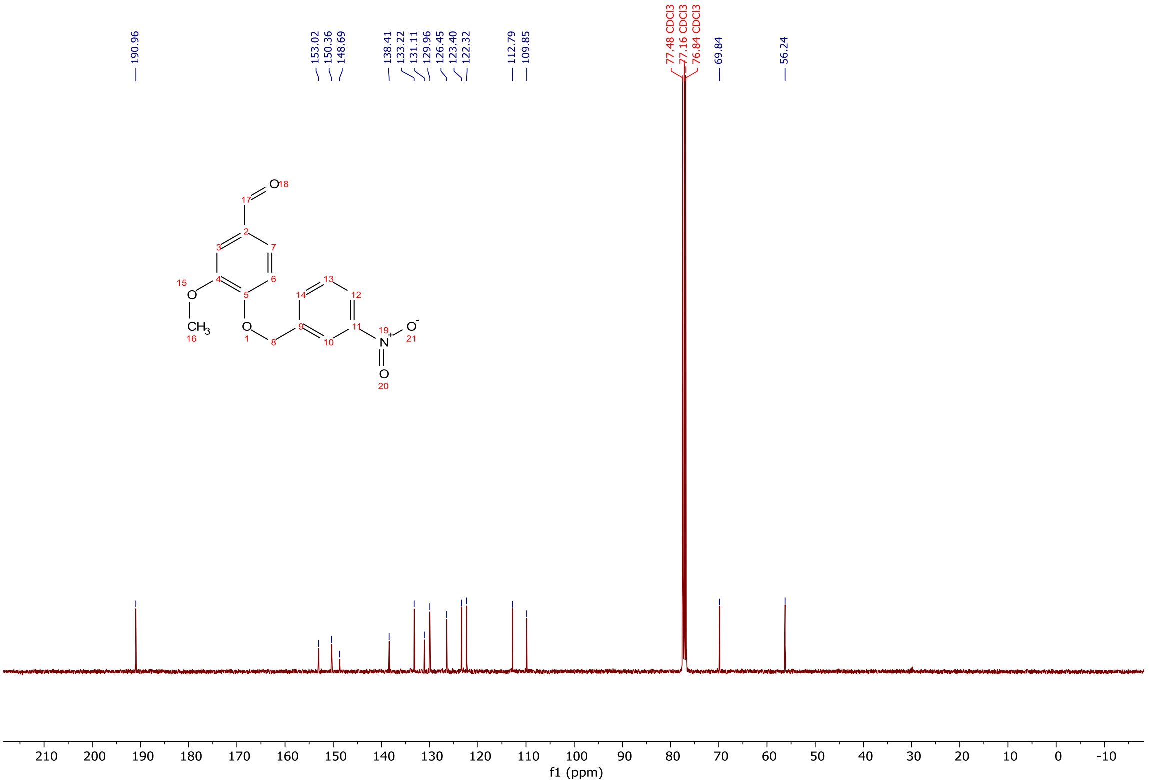

Figure S36. ^{13}C NMR of compound **19V**.

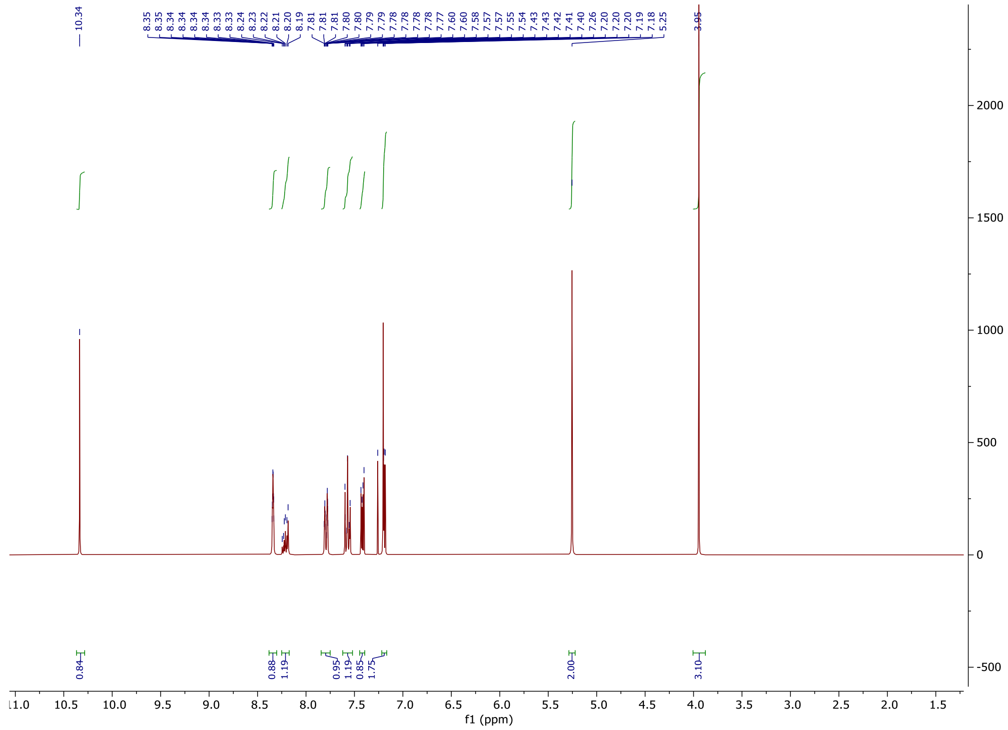

Figure S37. ^{1}H NMR of compound **20oV**.

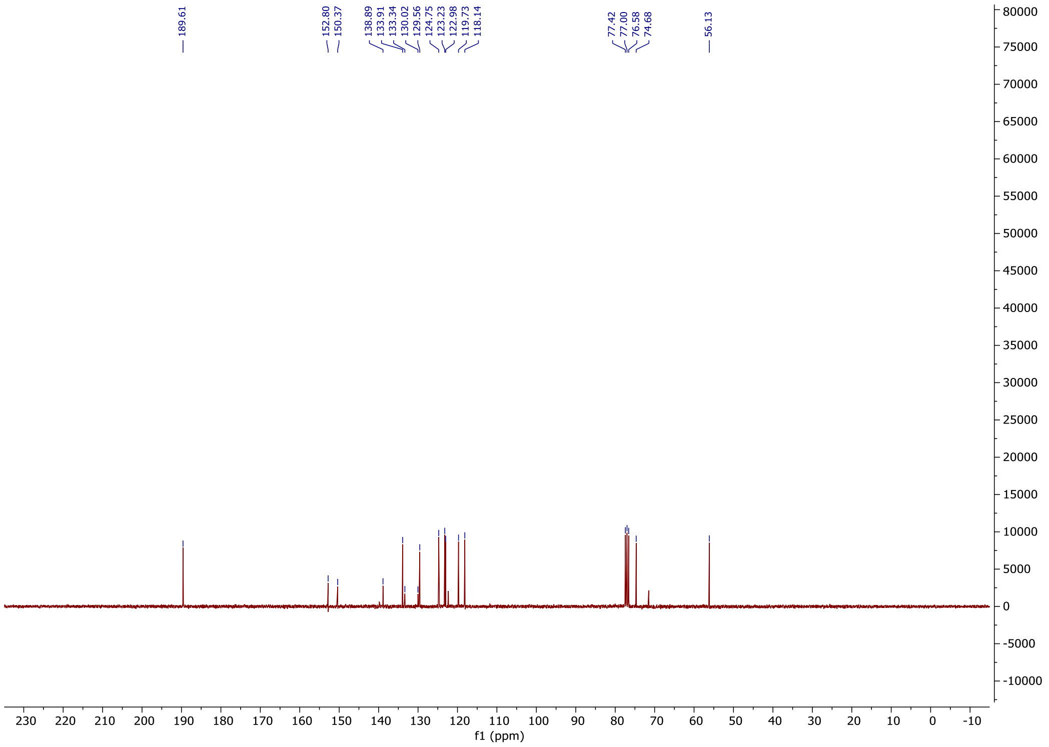

Figure S38. ^{13}C NMR of compound **20oV**.

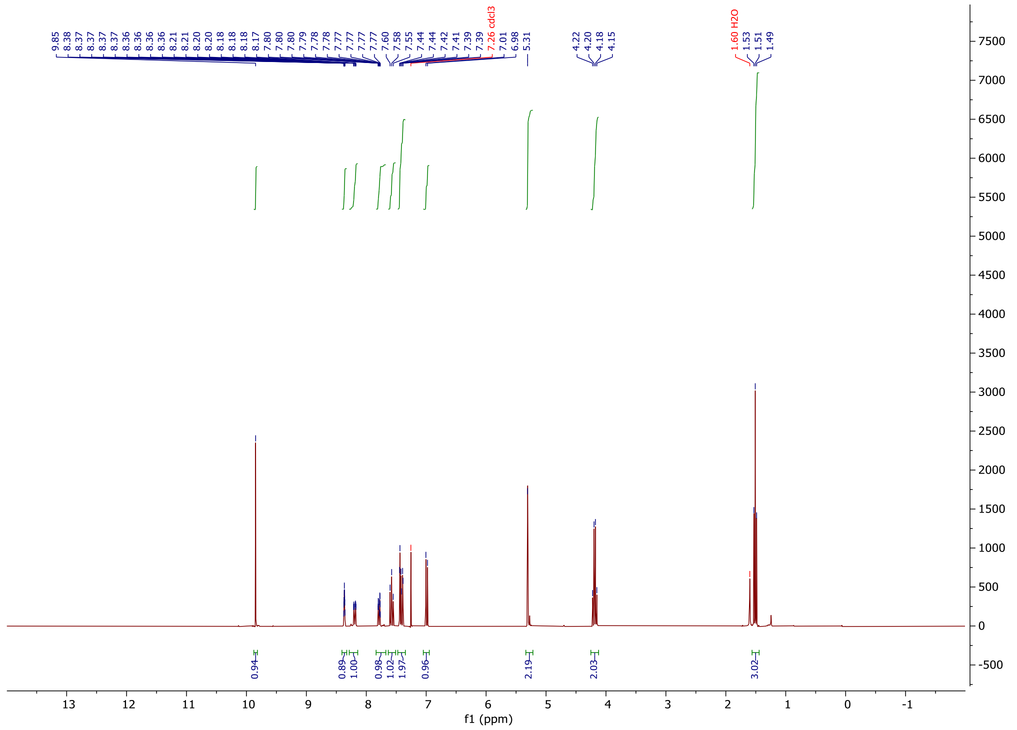

Figure S39. ^{1}H NMR of compound **21eV**.

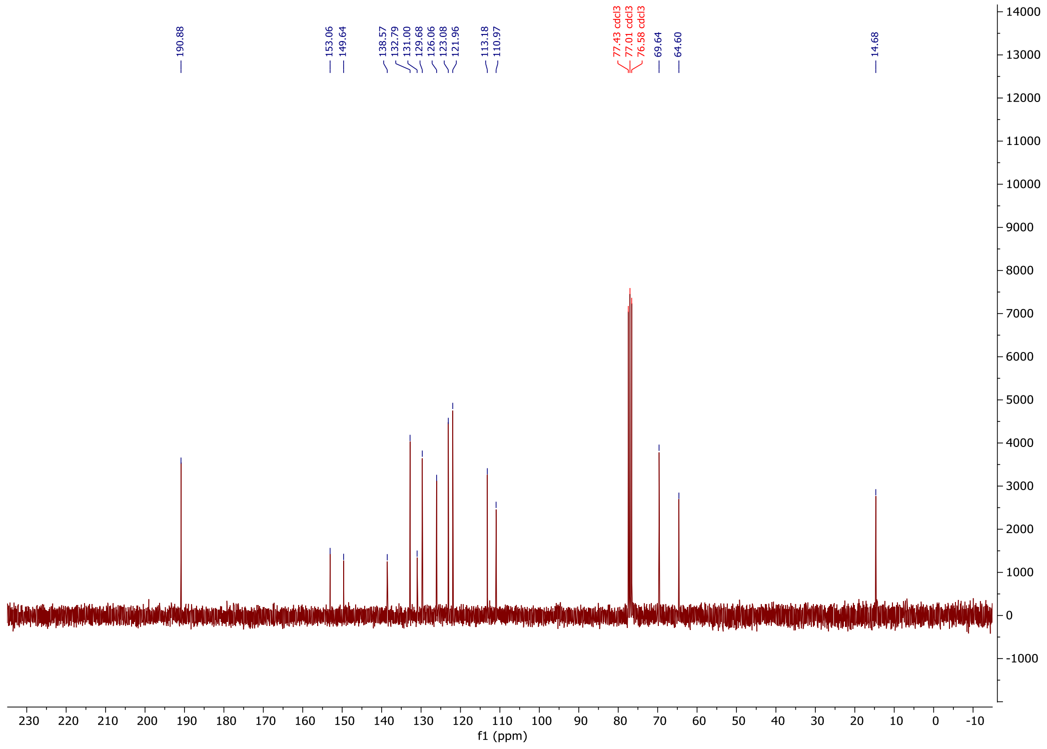

Figure S40. ^{13}C NMR of compound **21eV**.

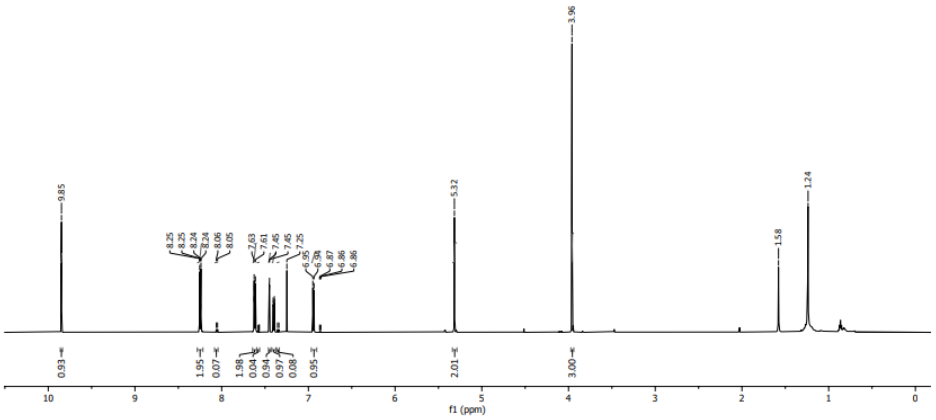

Figure S41. ^{1}H NMR of compound **22V**.

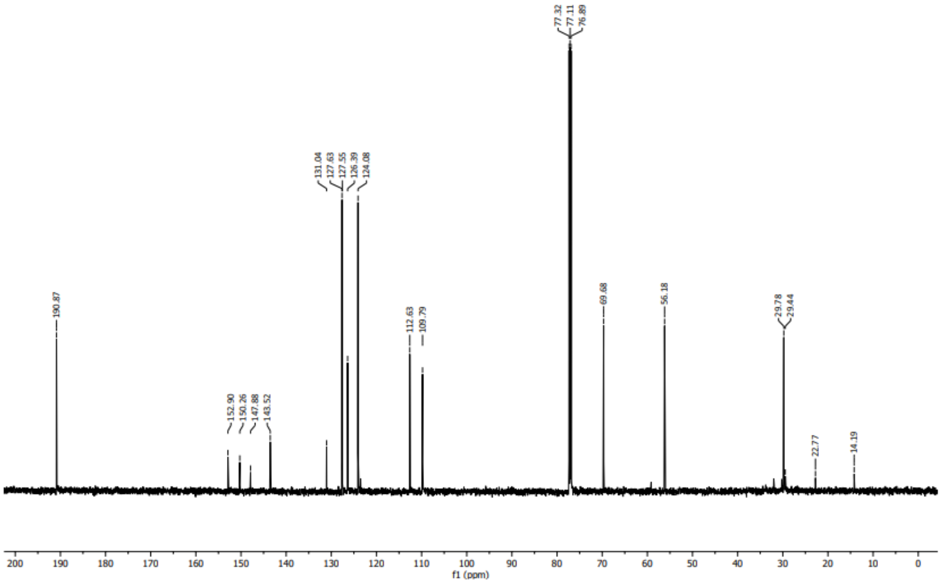

Figure S42. ^{13}C NMR of compound **22V**.

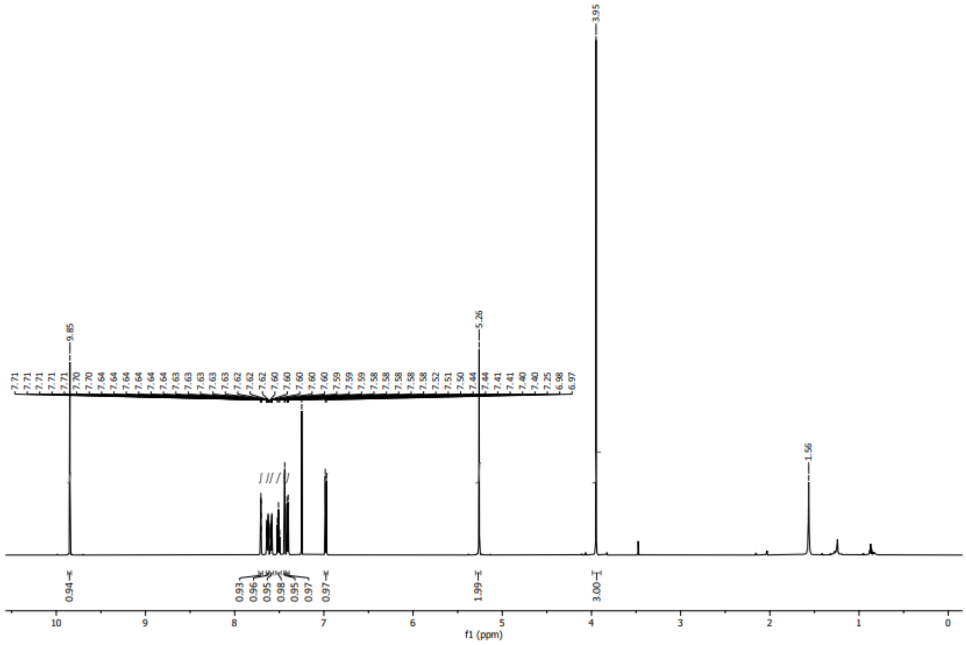

Figure S43. ^{1}H NMR of compound **23V**.

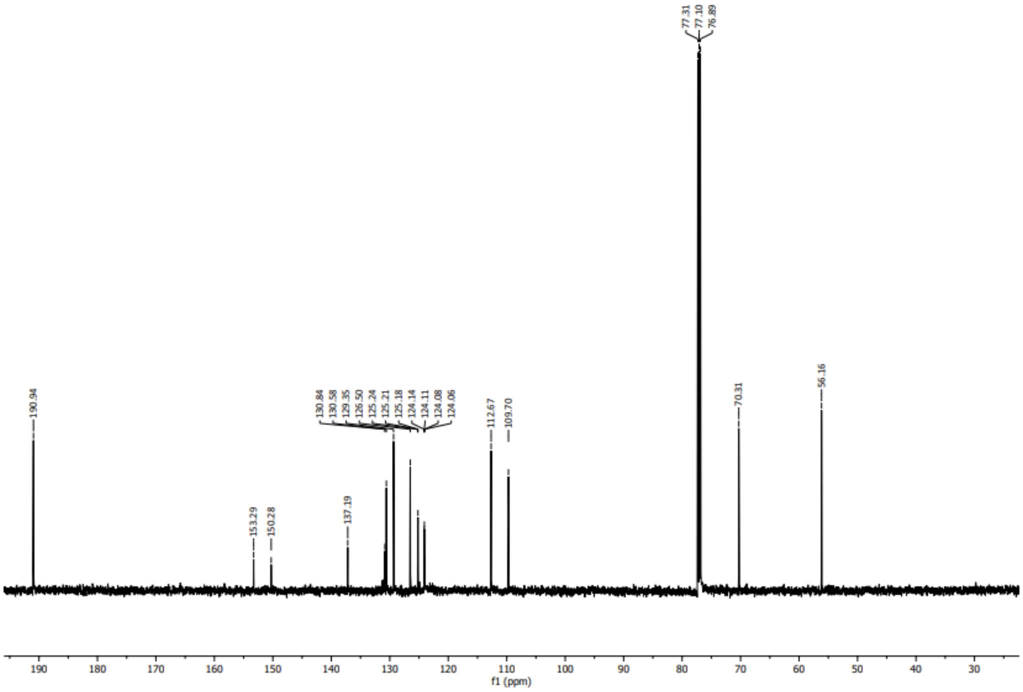

Figure S44. ^{13}C NMR of compound **23V**.

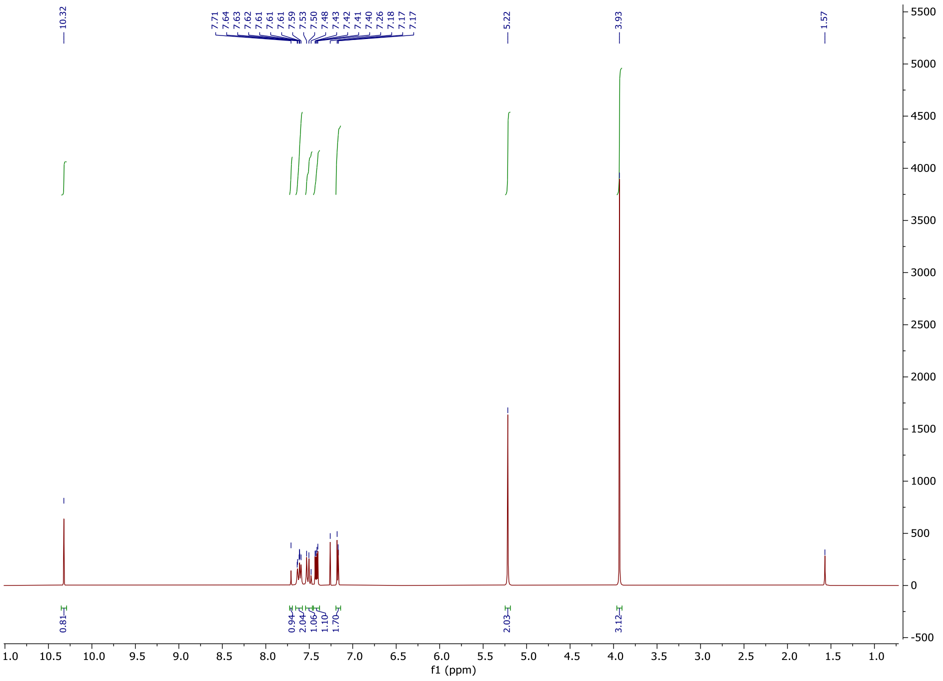

Figure S45. ^{1}H NMR of compound **24oV**.

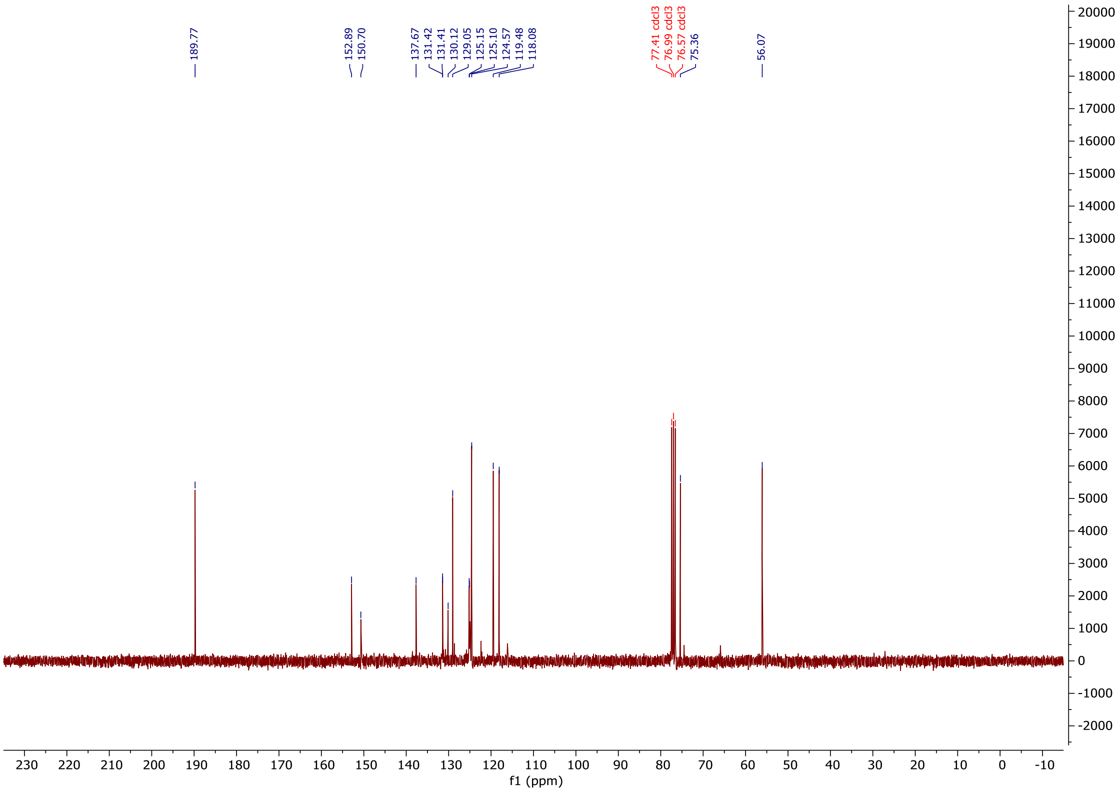

Figure S46. ^{13}C NMR of compound **24oV**.

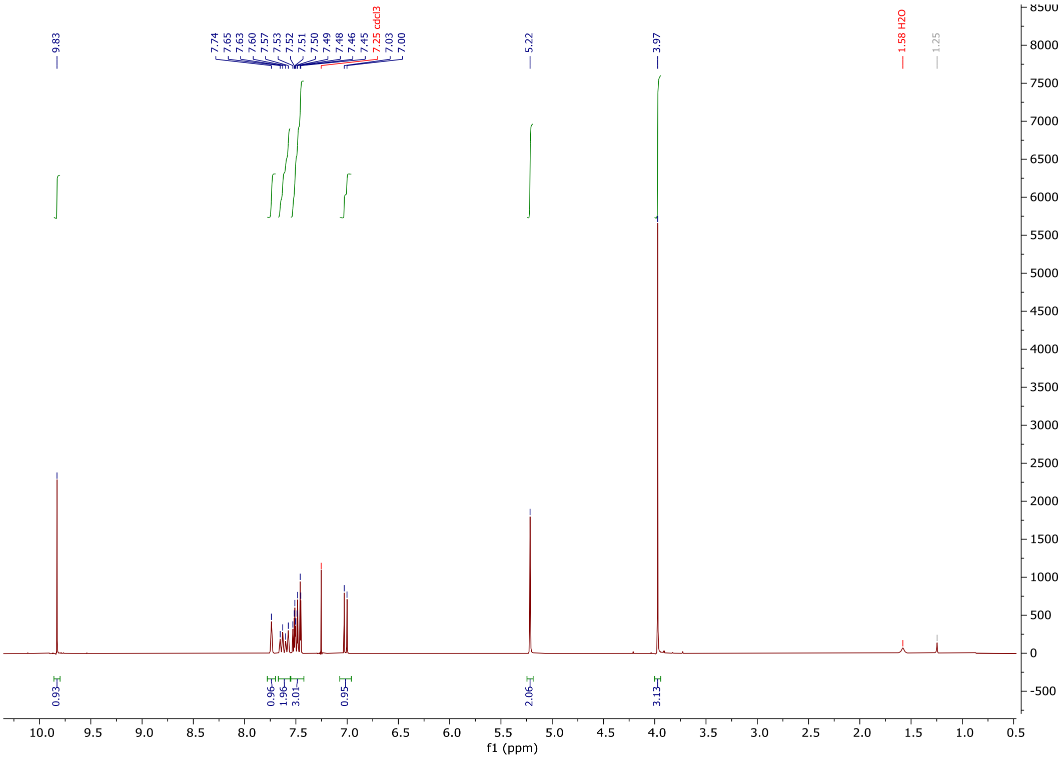

Figure S47. ^{1}H NMR of compound **25iV**.

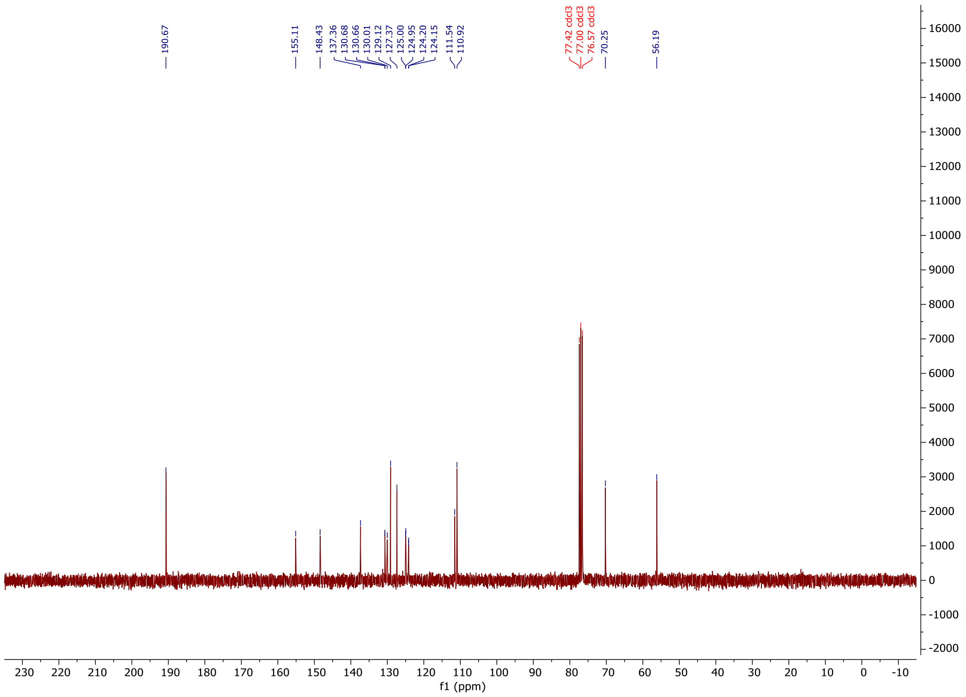

Figure S48. ^{13}C NMR of compound **25iV**.

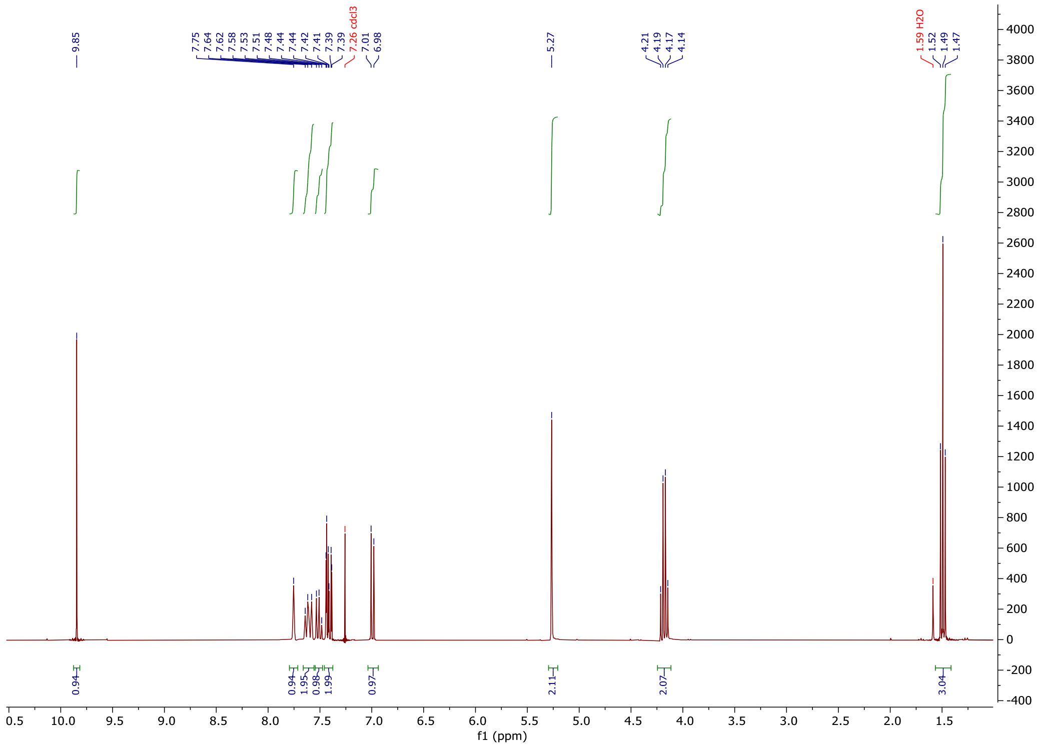

Figure S49. ^{1}H NMR of compound **26eV**.

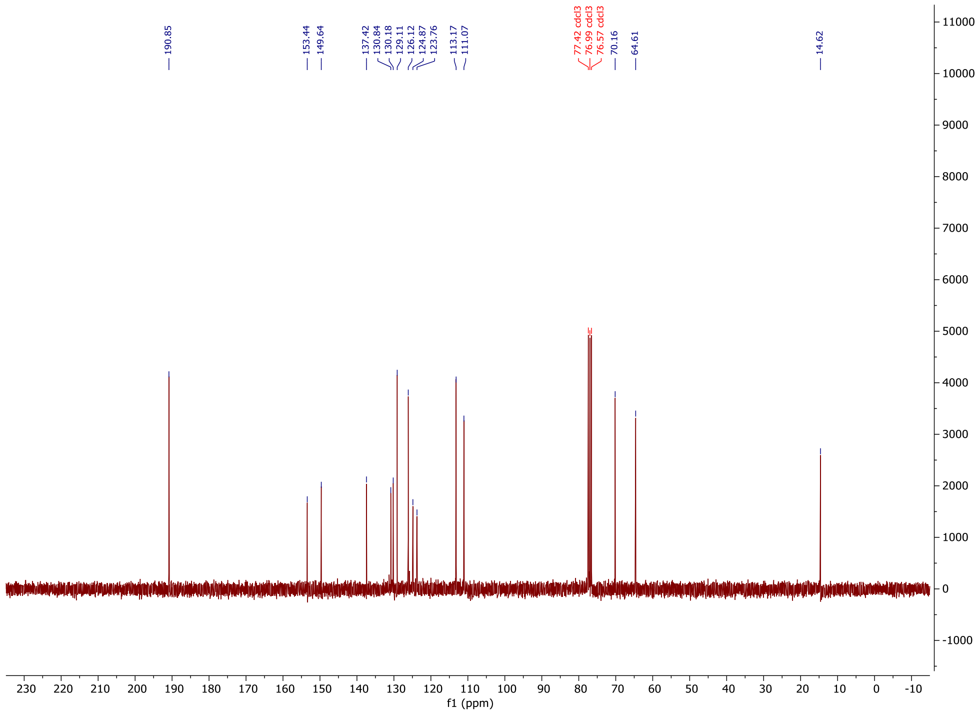

Figure S50. ^{13}C NMR of compound **26eV**.

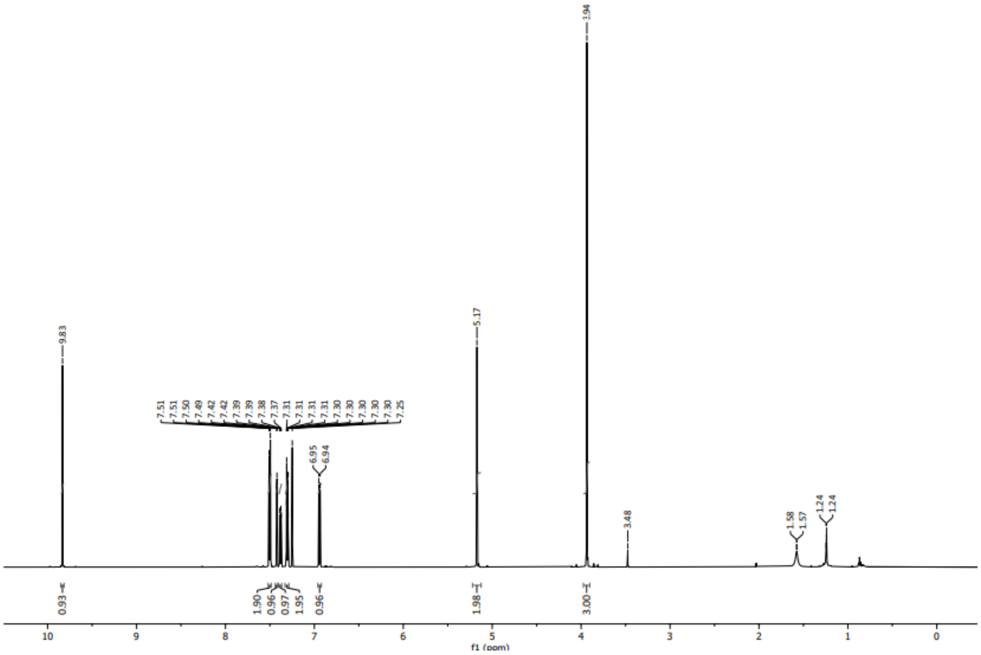

Figure S51. ^{1}H NMR of compound **27V**.

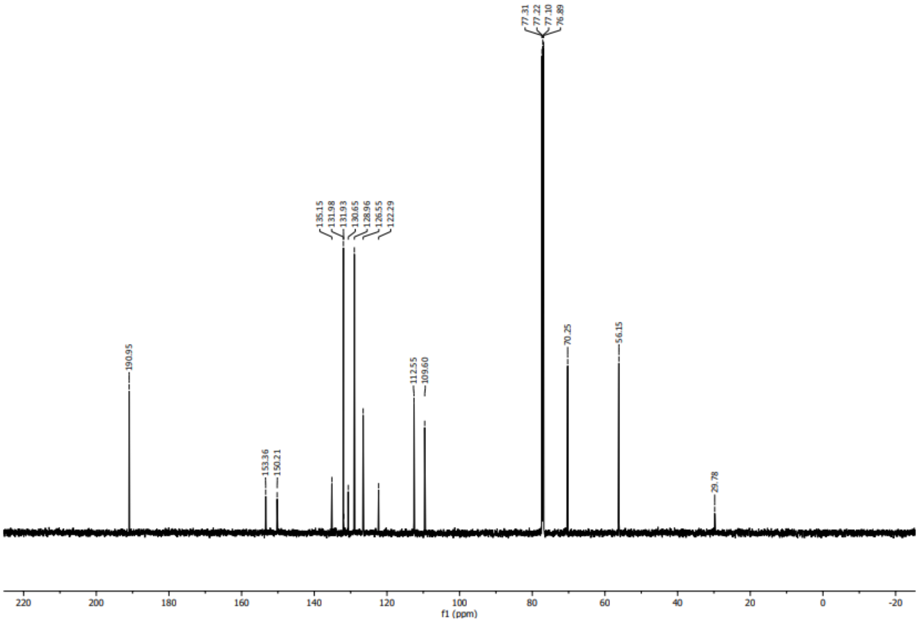

Figure S52. ^{13}C NMR of compound **27V**.

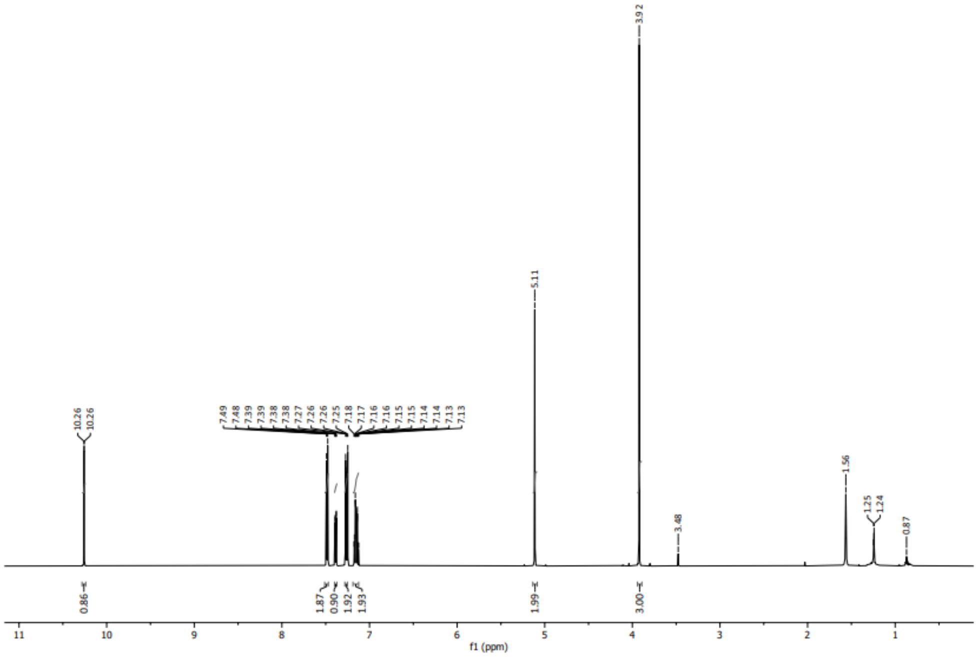

Figure S53. ^{1}H NMR of compound **28oV**.

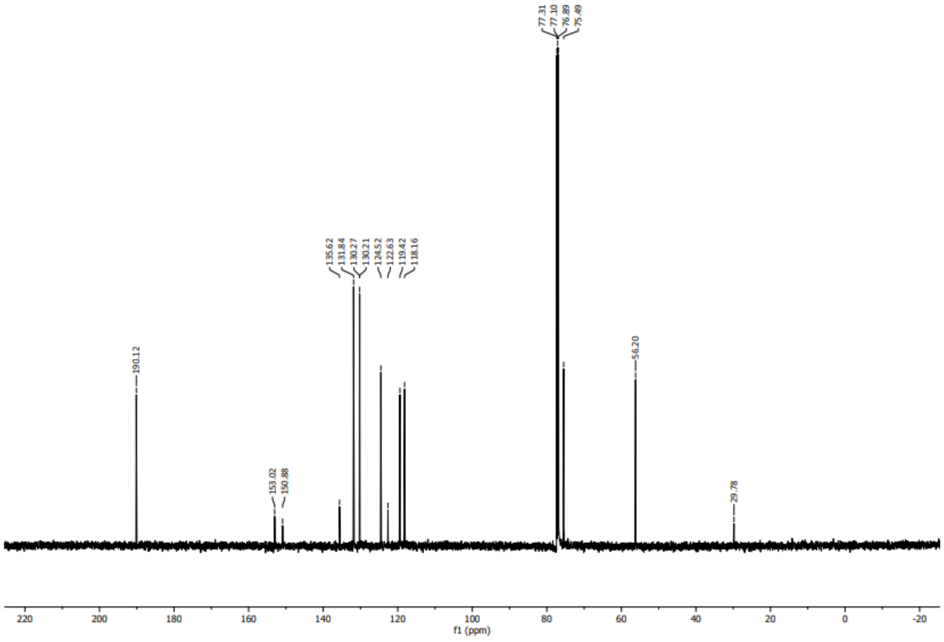

Figure S54. ^{13}C NMR of compound **28oV**.

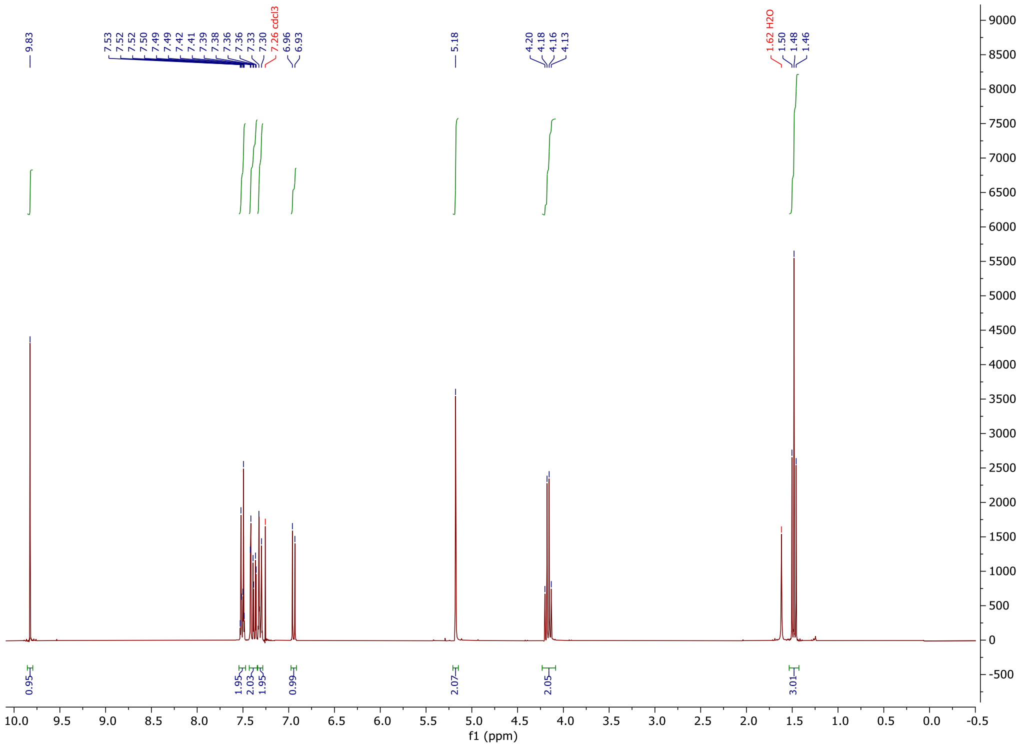

Figure S55. ^{1}H NMR of compound **29eV**.

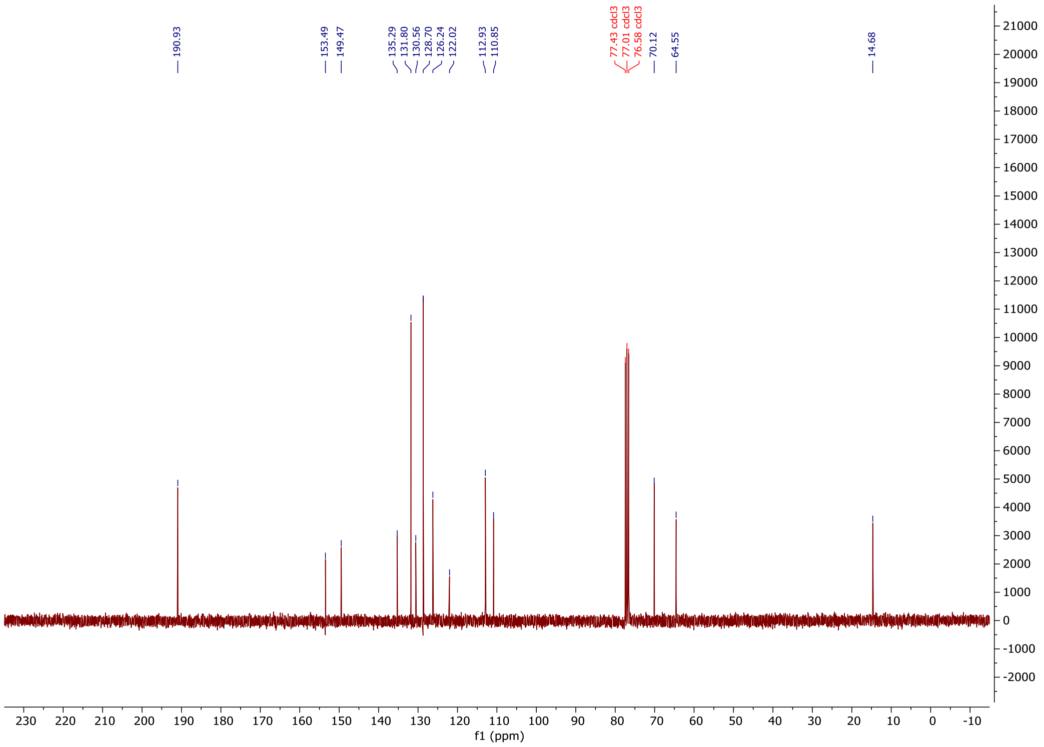

Figure S56. ^{13}C NMR of compound **29eV**.

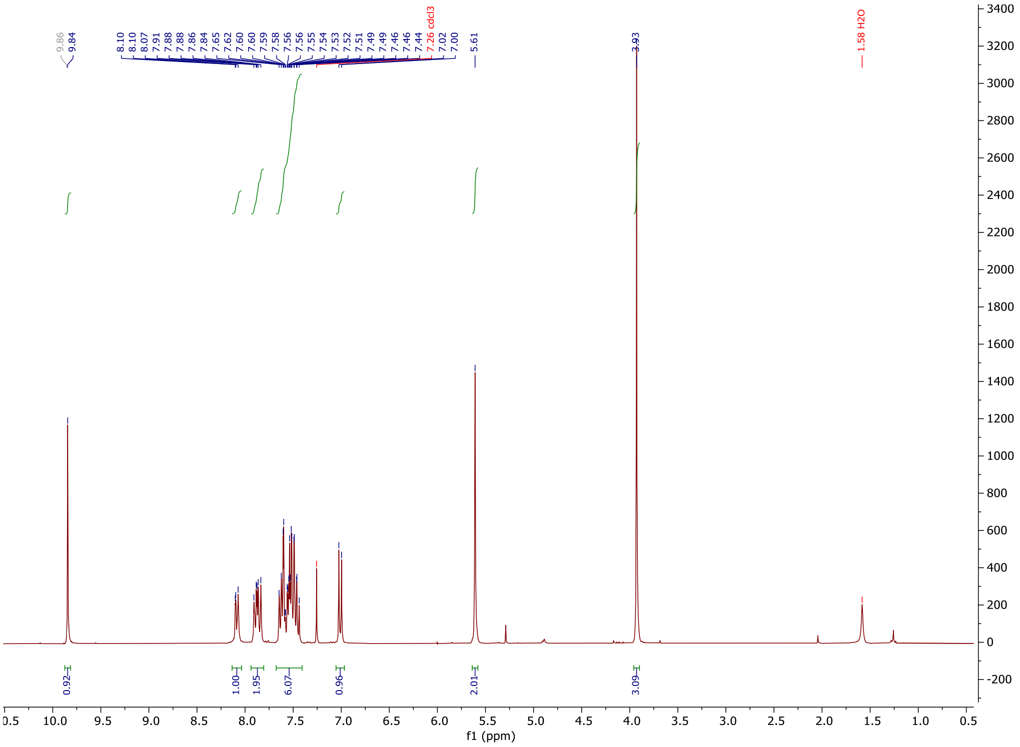

Figure S57. ^{1}H NMR of compound **30iV**.

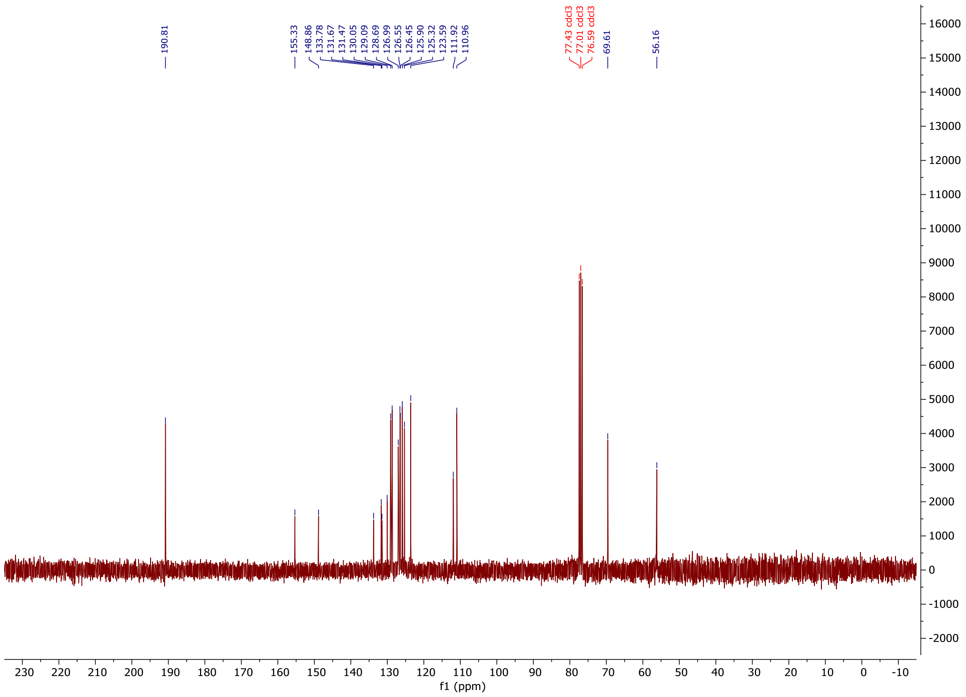

Figure S58. ^{13}C NMR of compound **30iV**.

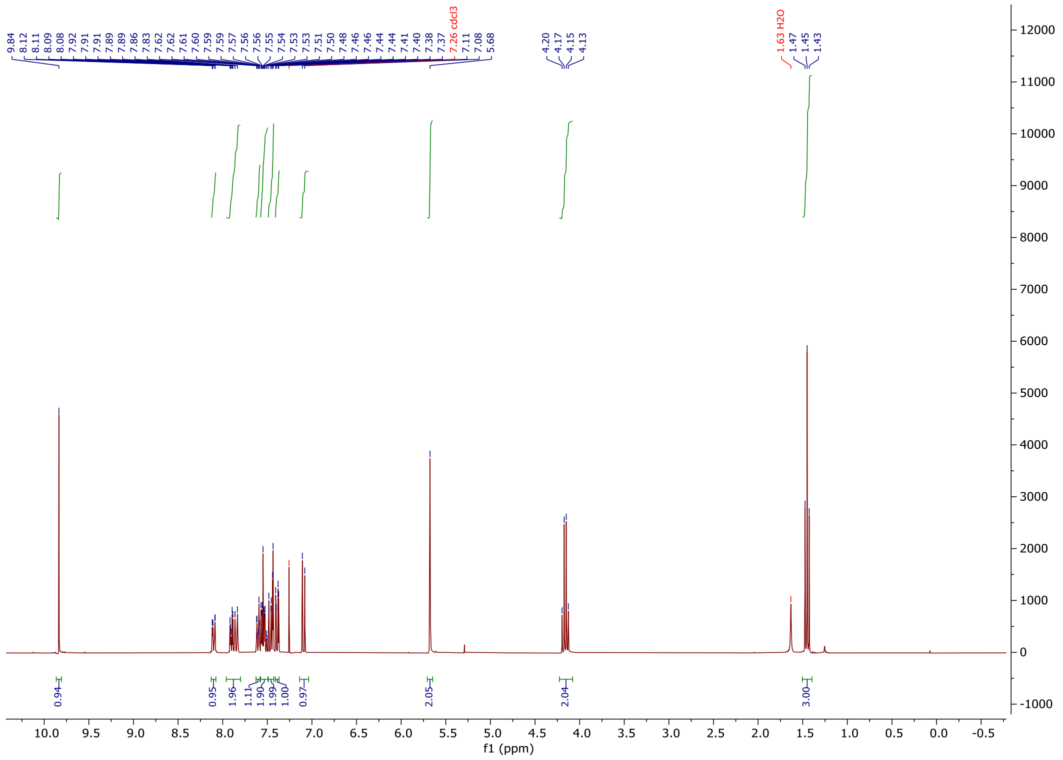

Figure S59. ^{1}H NMR of compound **31eV**.

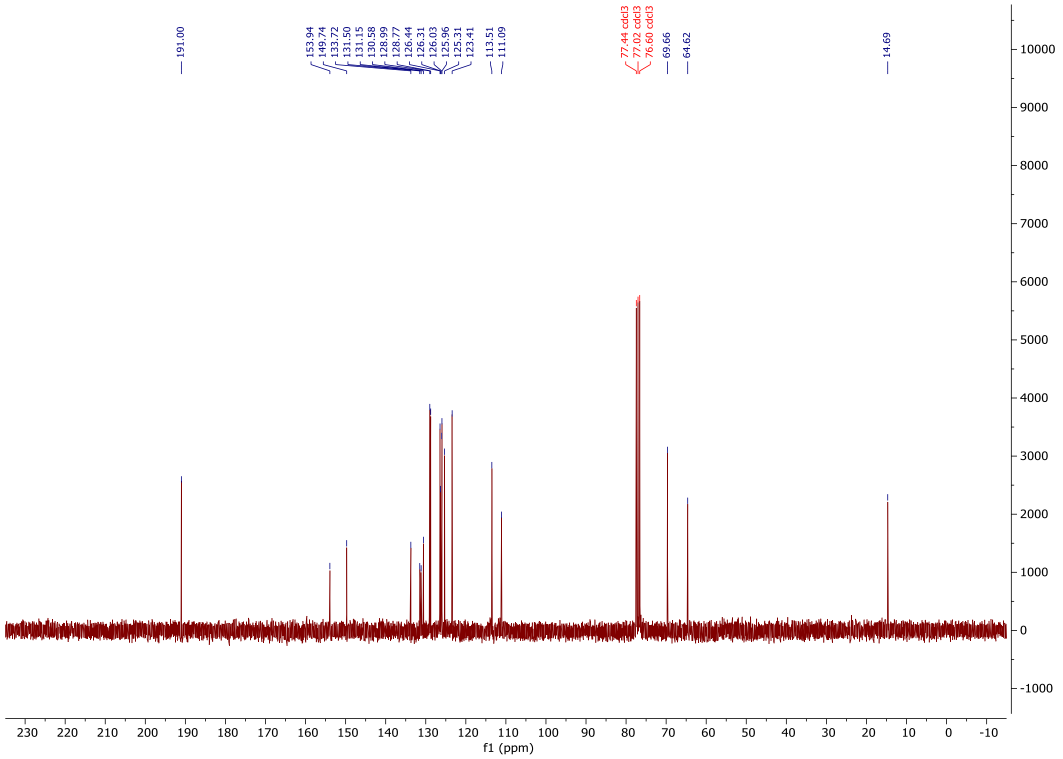

Figure S60. ^{13}C NMR of compound **31eV**.

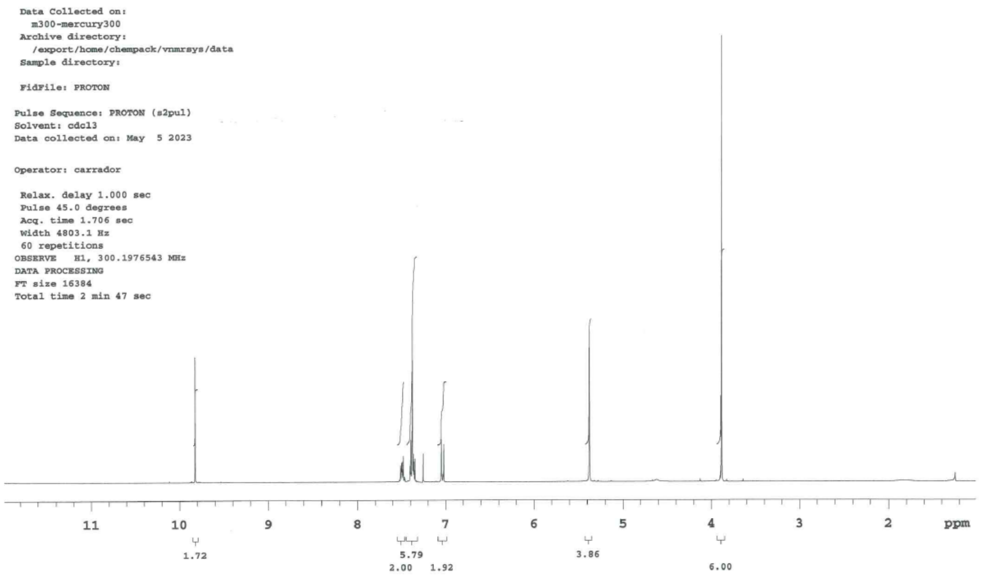

Figure S61. ^{1}H NMR of compound **32V**.

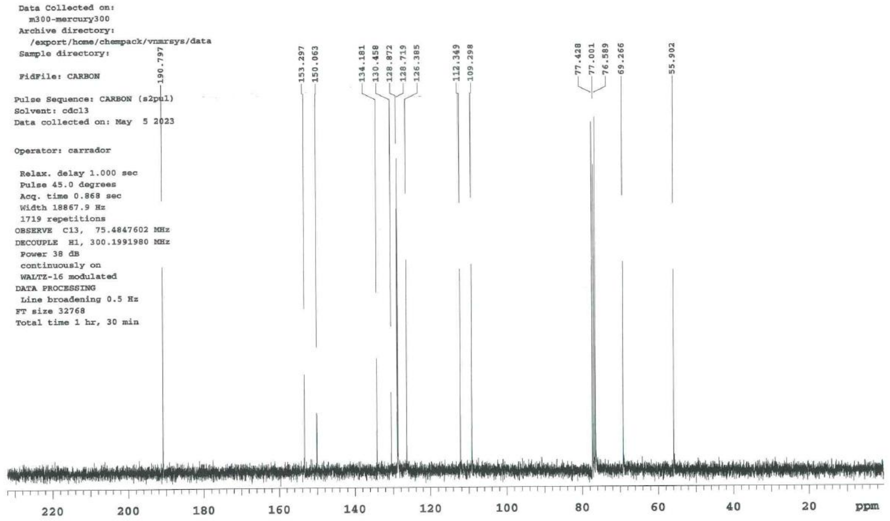

Figure S62. ^{13}C NMR of compound **32V**.

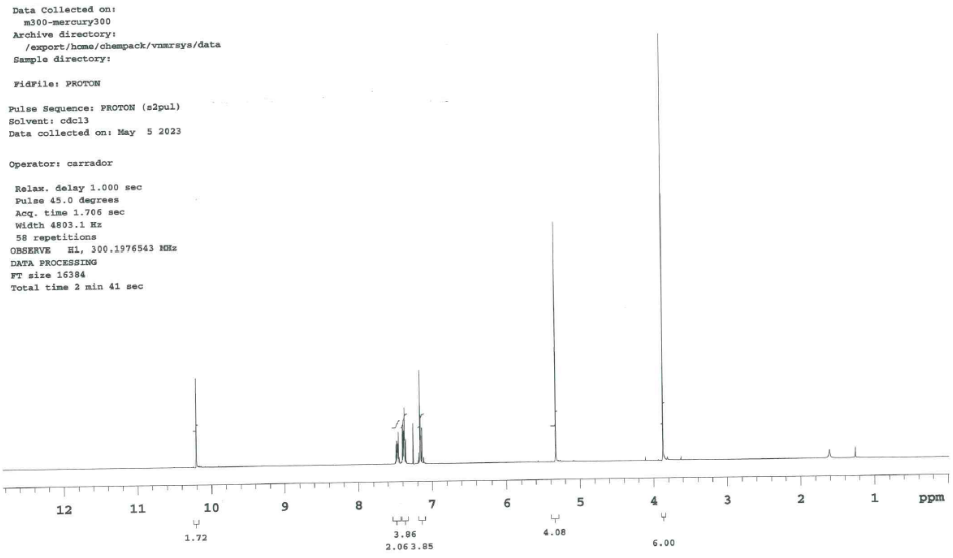

Figure S63. ^{1}H NMR of compound **33oV**.

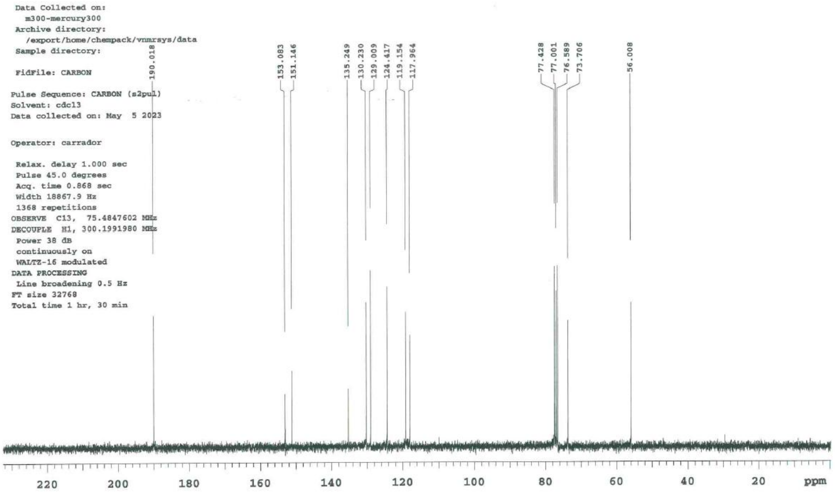

Figure S64. ^{13}C NMR of compound **33oV**.

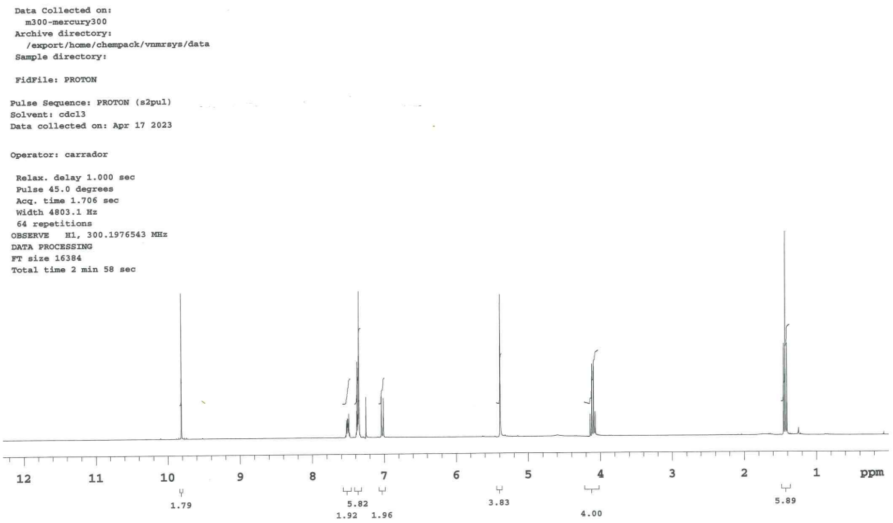

Figure S65. ^{1}H NMR of compound **34eV**.

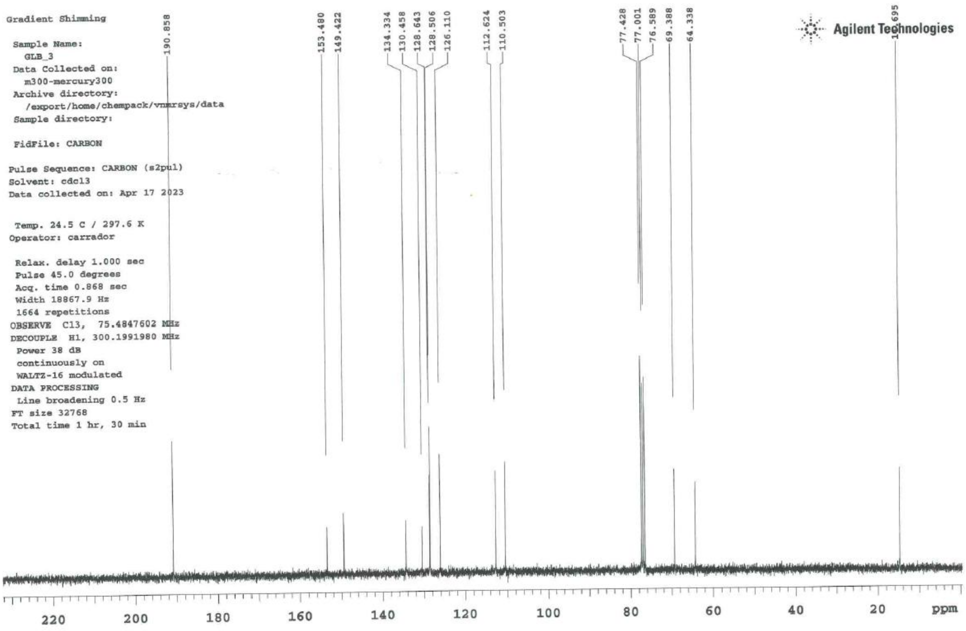

Figure S66. ^{13}C NMR of compound **34eV**.

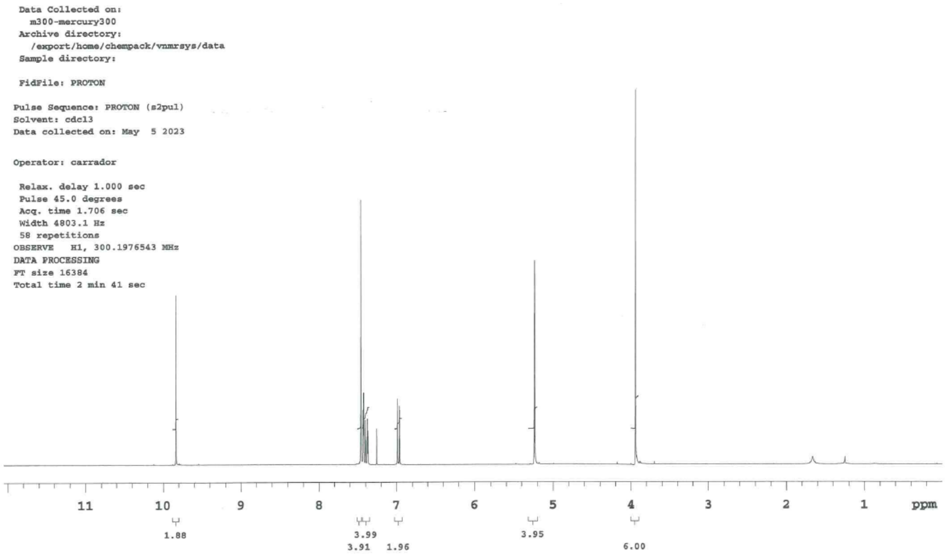

Figure S67. ^{1}H NMR of compound **35V**.

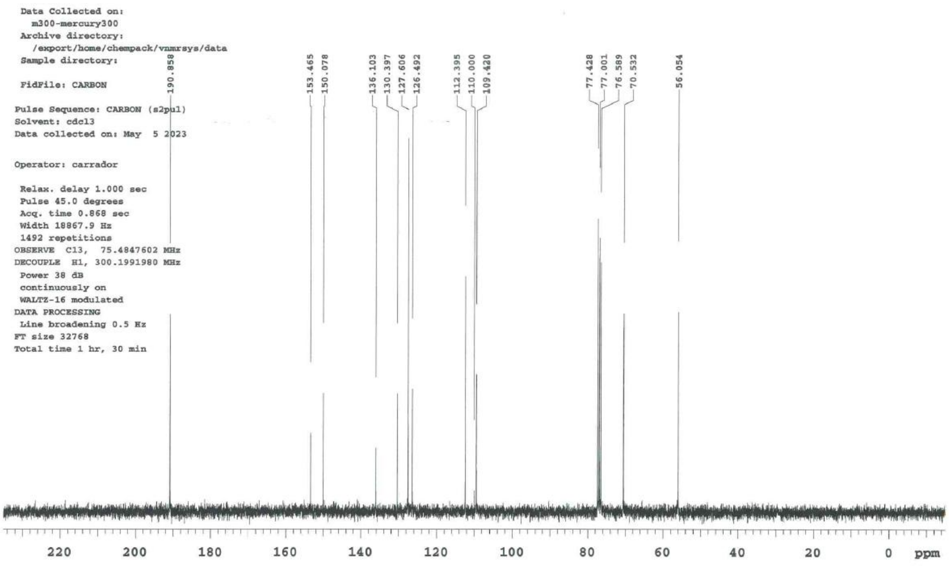

Figure S68. ^{13}C NMR of compound **35V**.

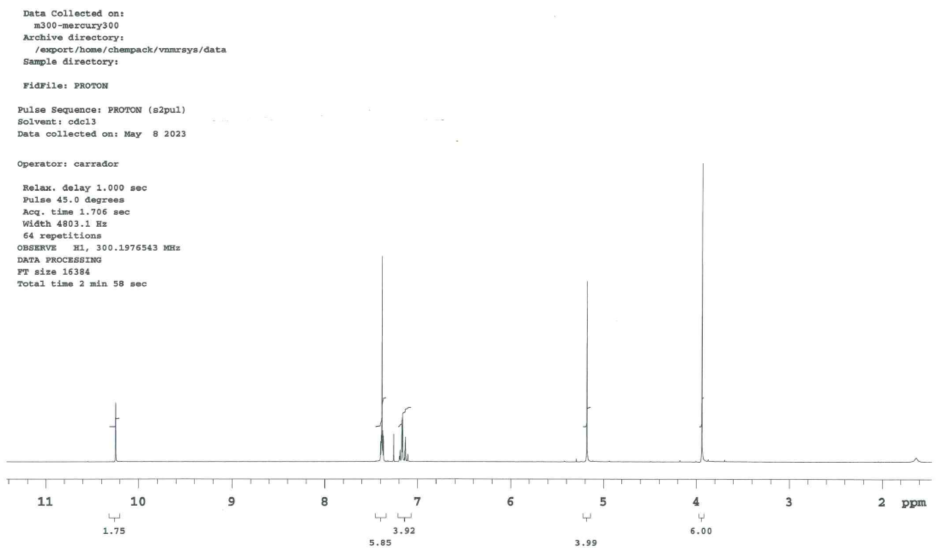

Figure S69. ^{1}H NMR of compound **36oV**.

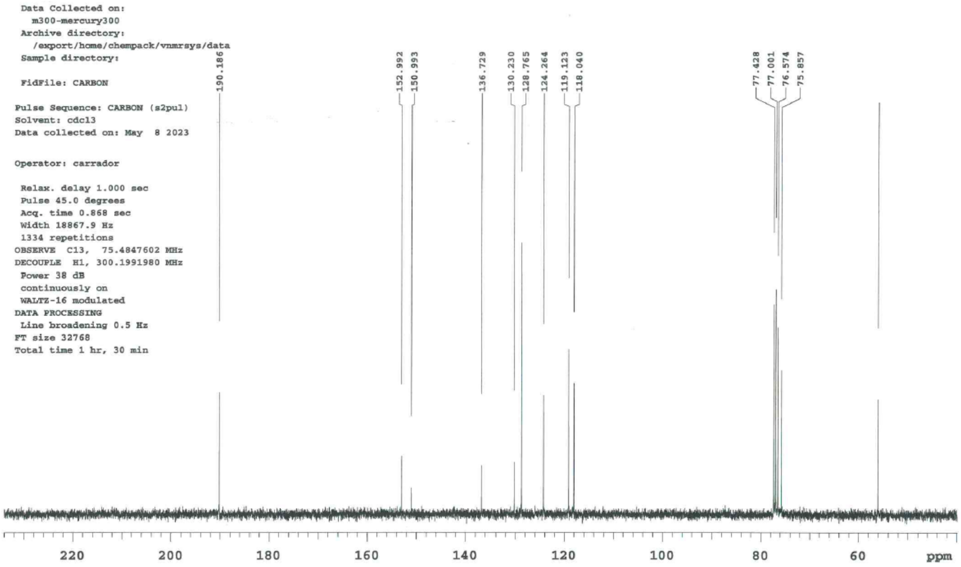

Figure S70. ^{13}C NMR of compound **36oV**.

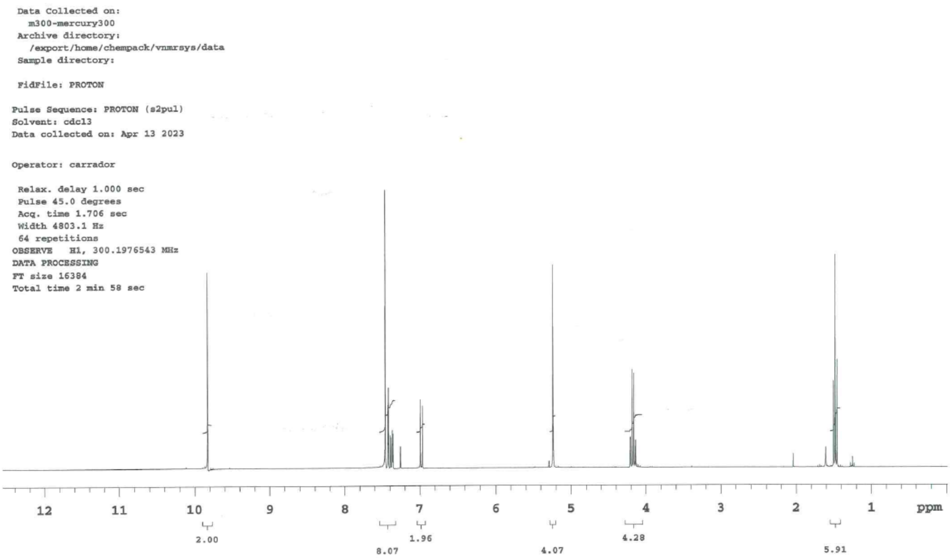

Figure S71. ^{1}H NMR of compound **37eV**.

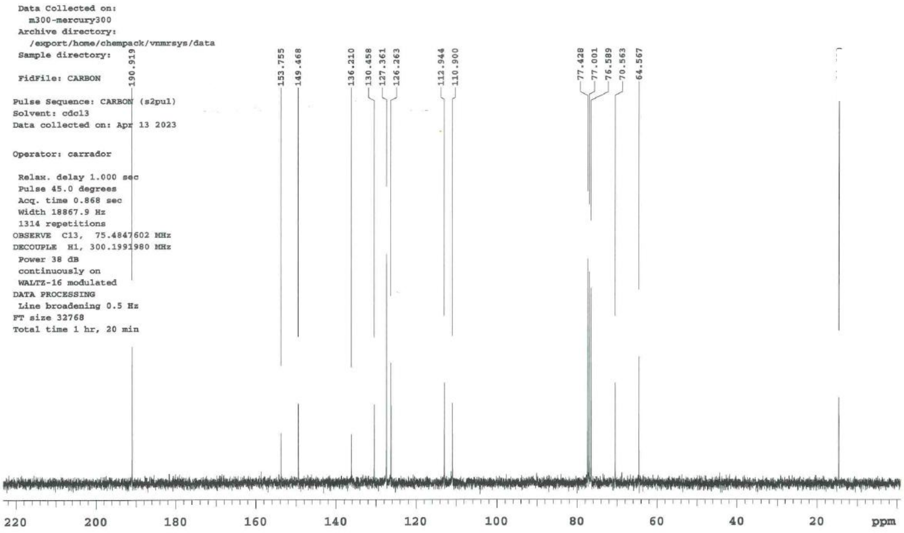

Figure S72. ^{13}C NMR of compound **37eV**.

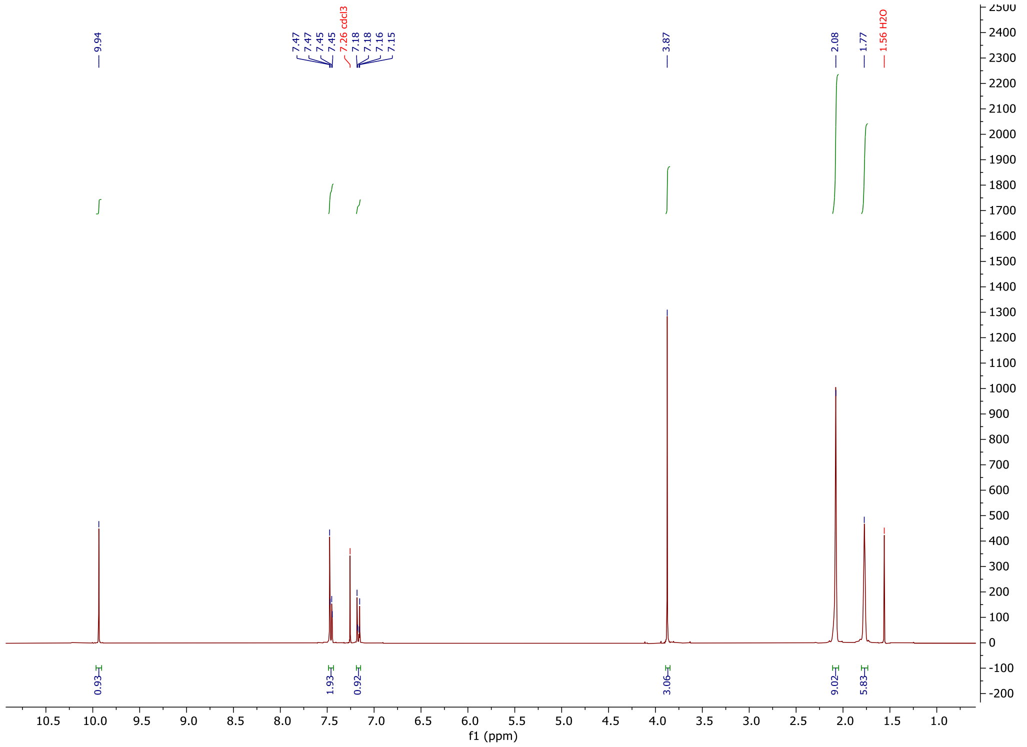

Figure S73. ^{1}H NMR of compound **38V**.

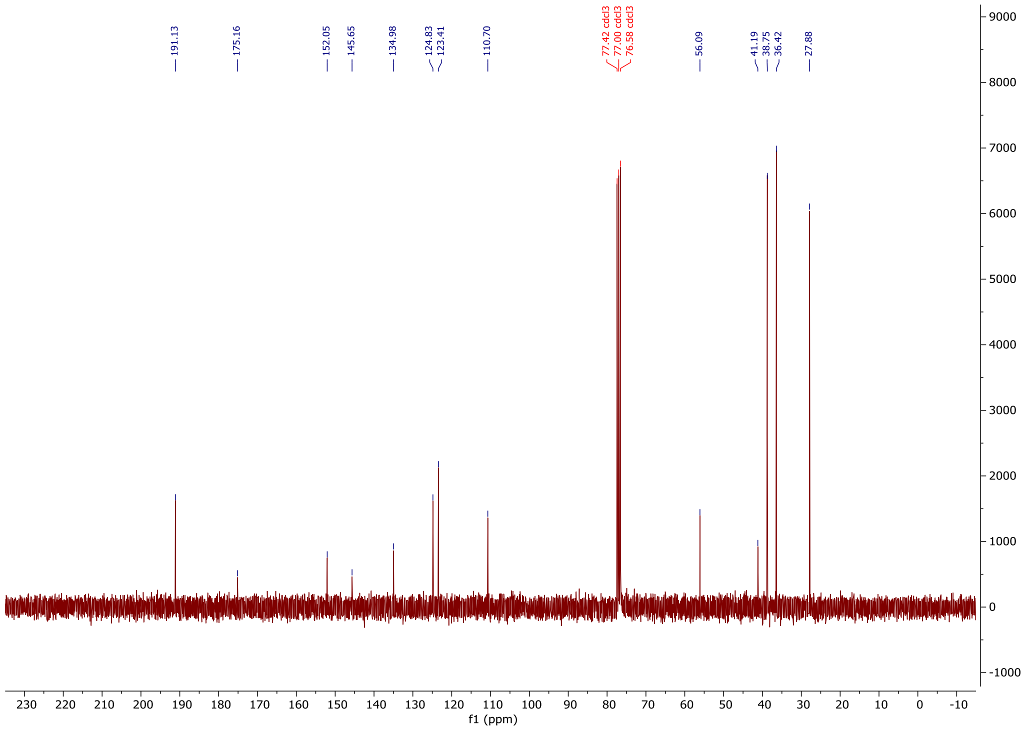

Figure S74. ^{13}C NMR of compound **38V**.

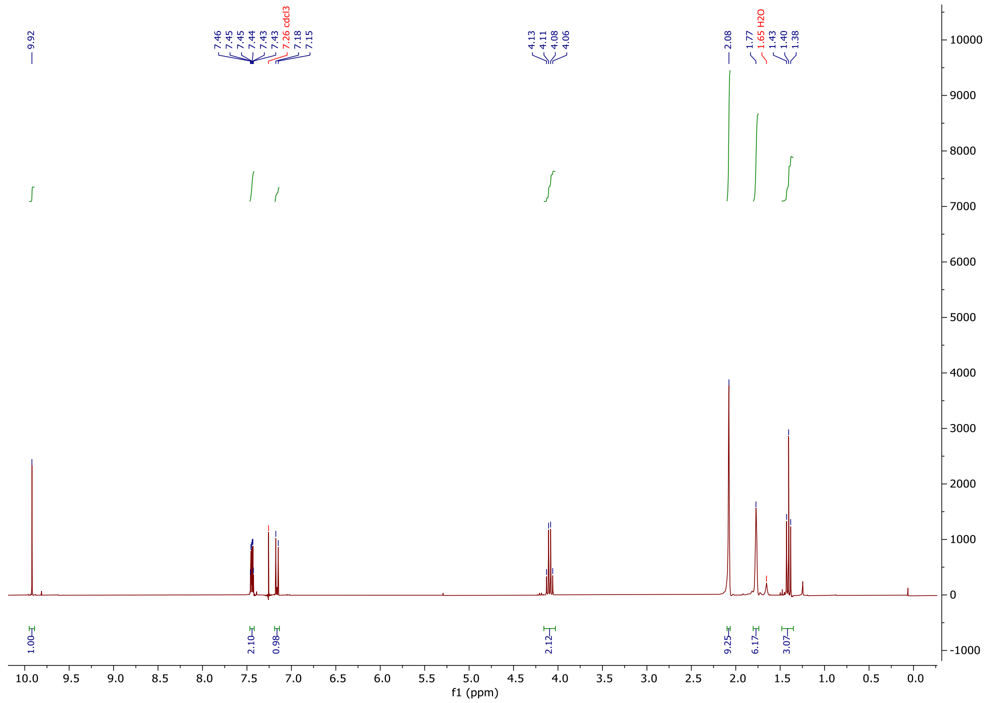

Figure S75. ^{1}H NMR of compound **39eV**.

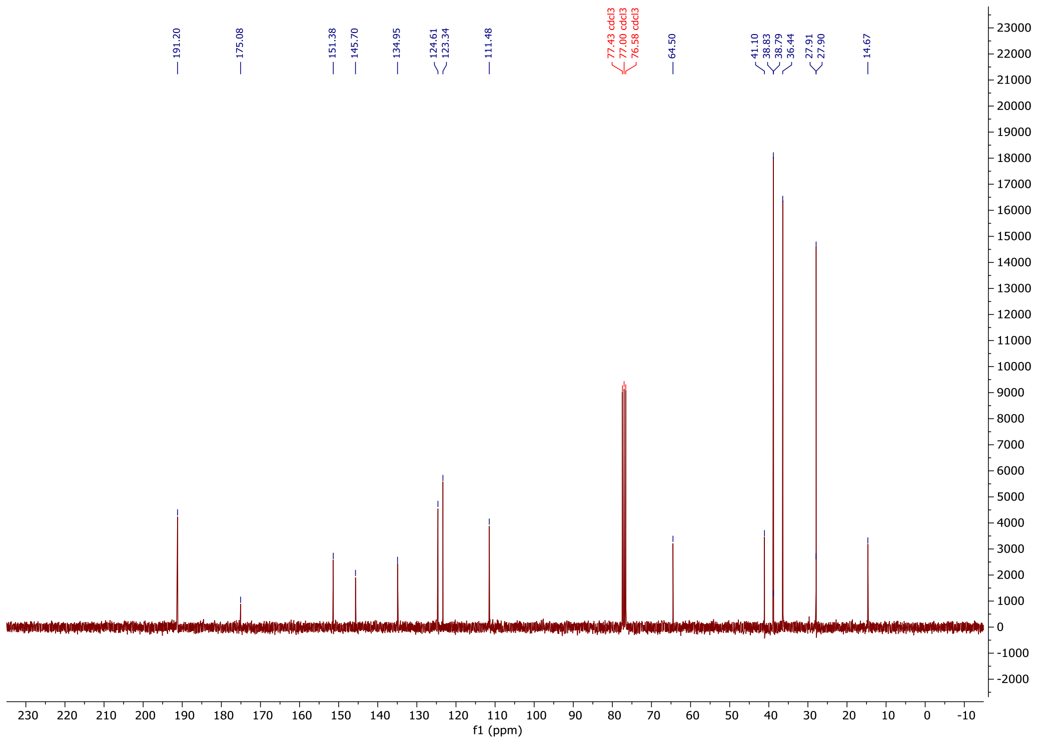

Figure S76. ^{13}C NMR of compound **39eV**.

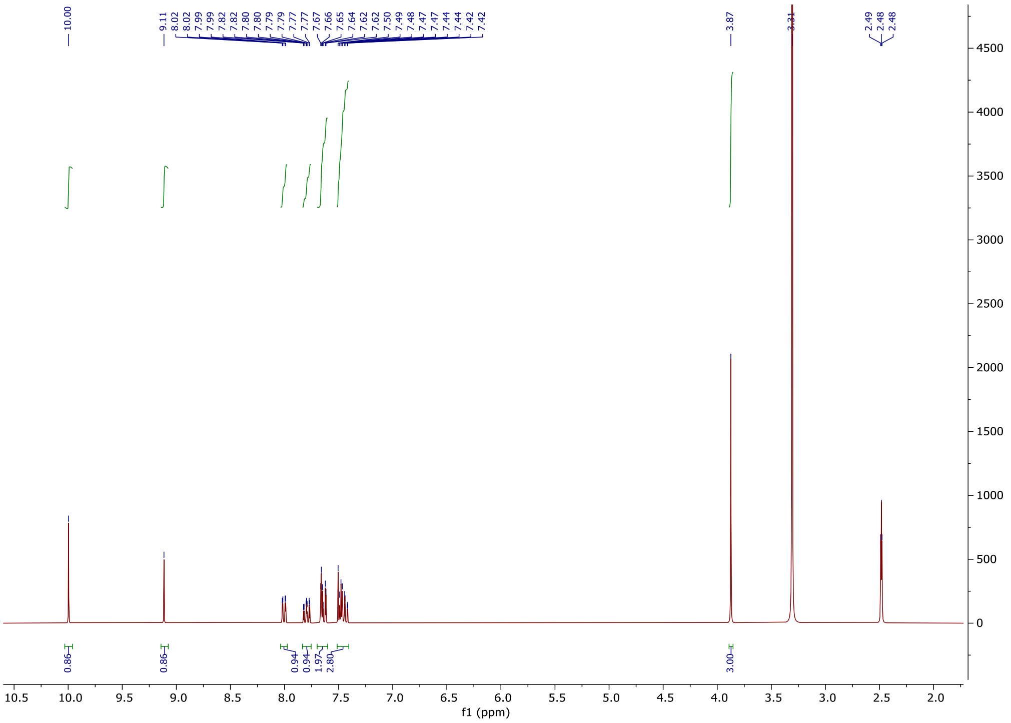

Figure S77. ^{1}H NMR of compound **40V**.

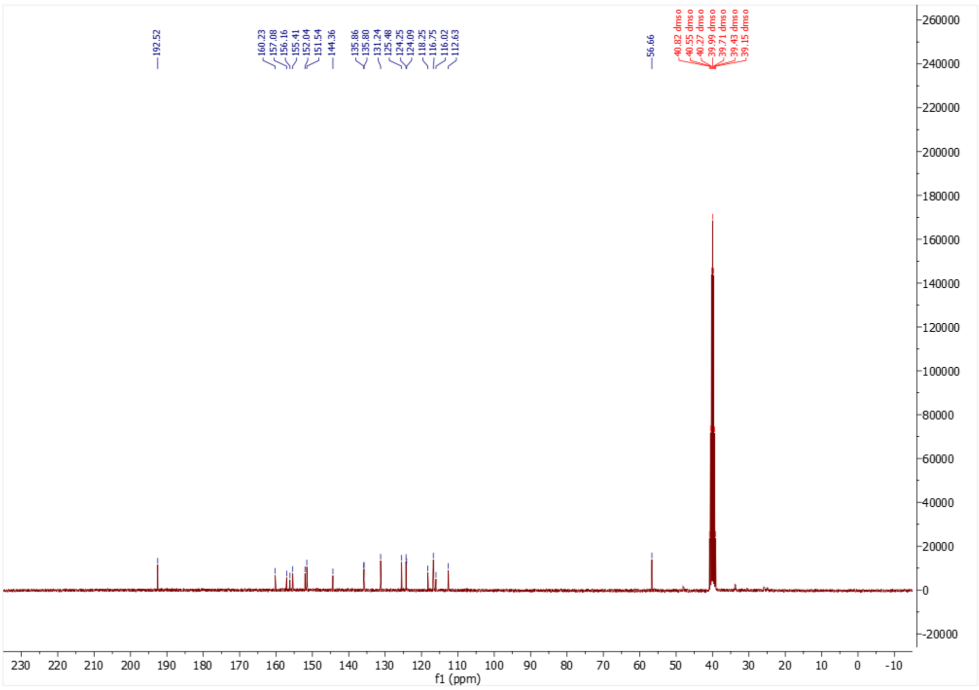

Figure S78. ^{13}C NMR of compound **40V**.

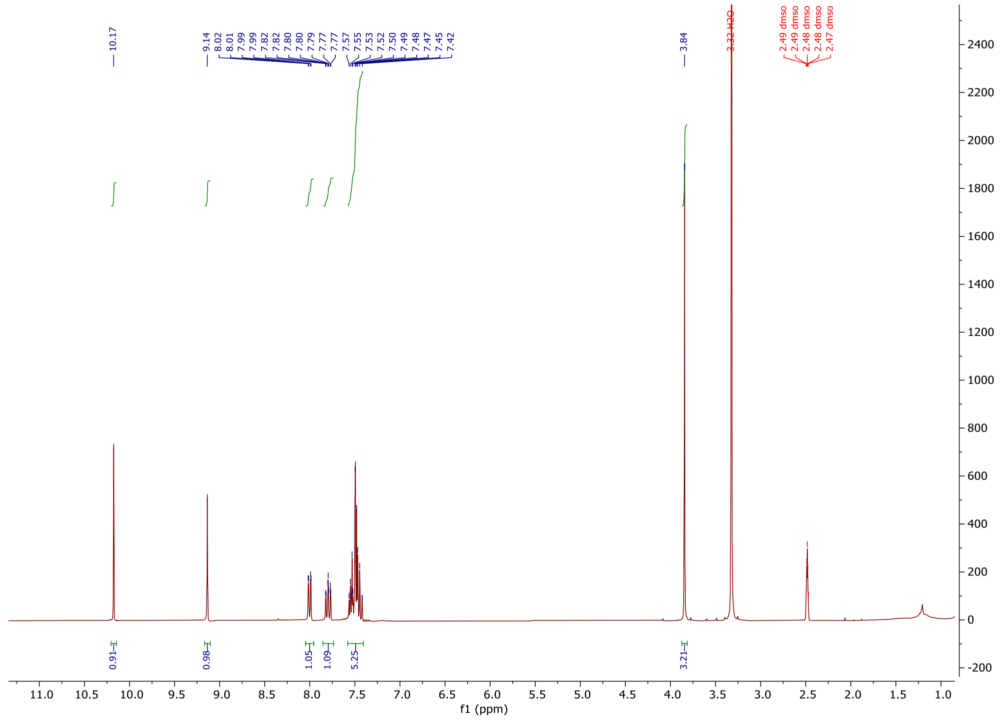

Figure S79 ^{1}H NMR of compound **41oV**.

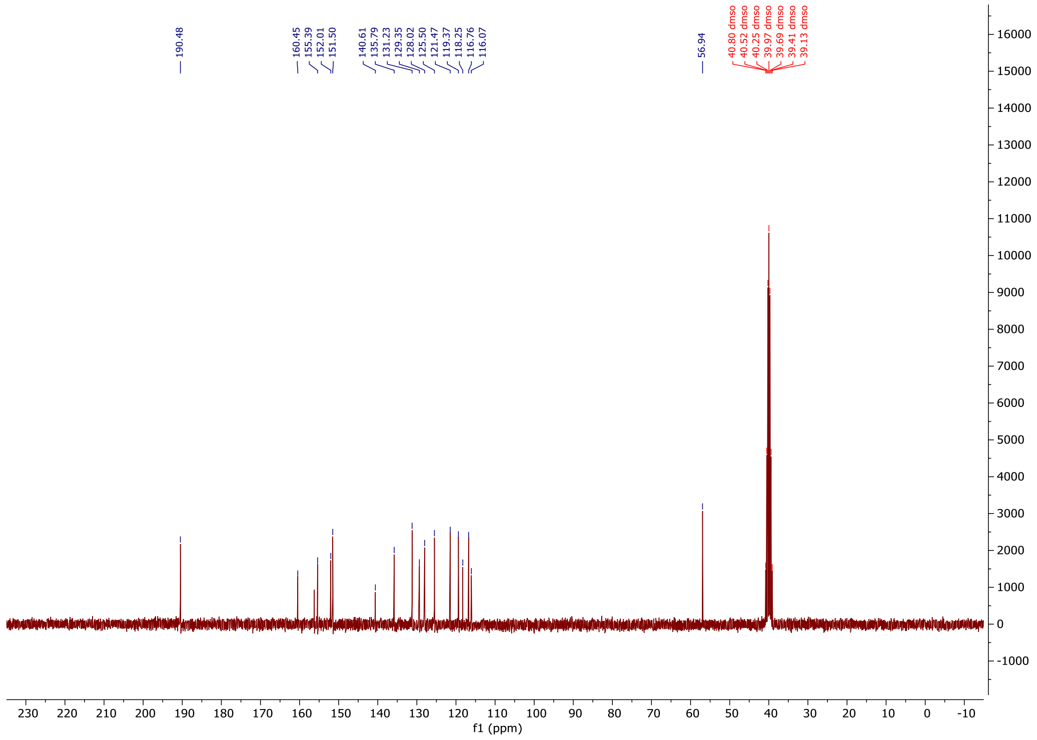

Figure S80. ^{13}C NMR of compound **41oV**.

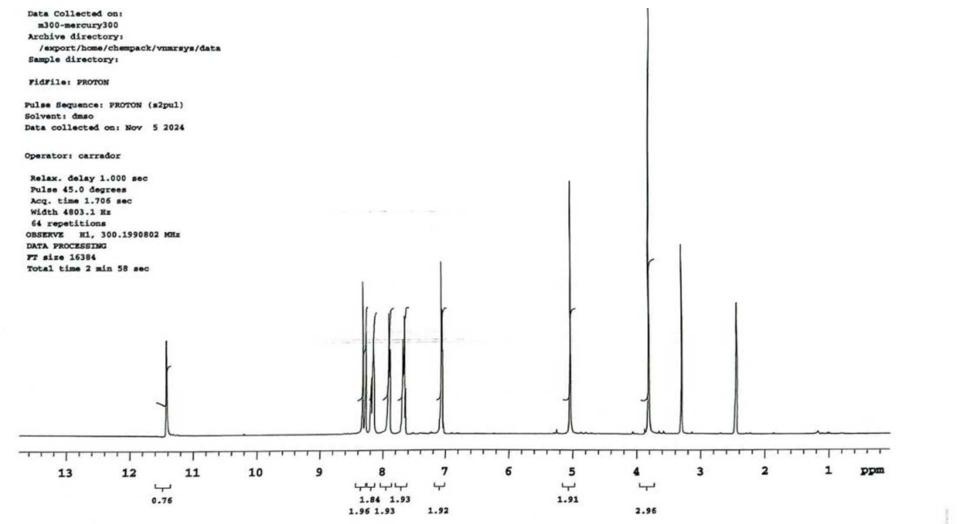

Figure S81. ^{1}H NMR of compound **42oV**.

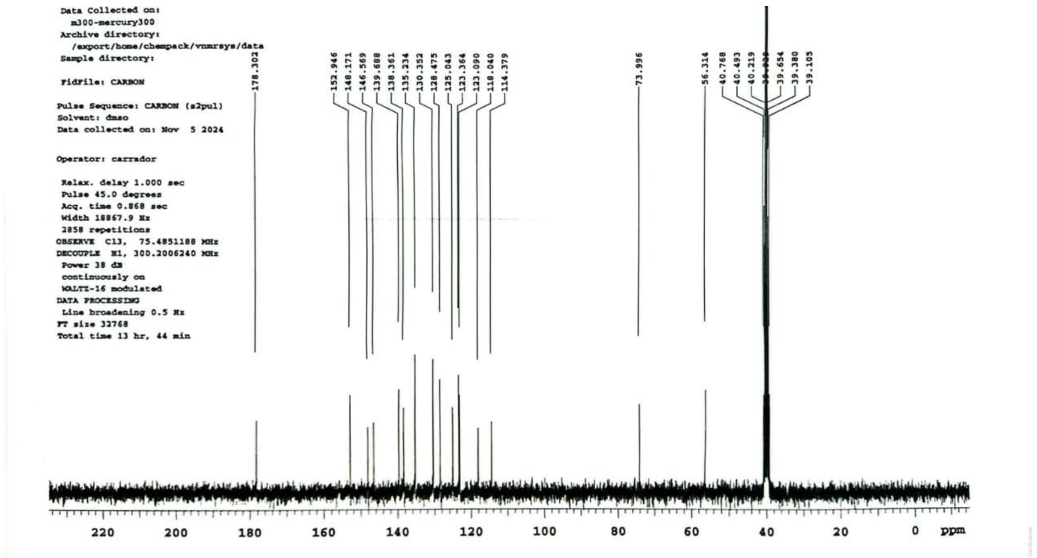

Figure S82. ^{13}C NMR of compound **42oV**.

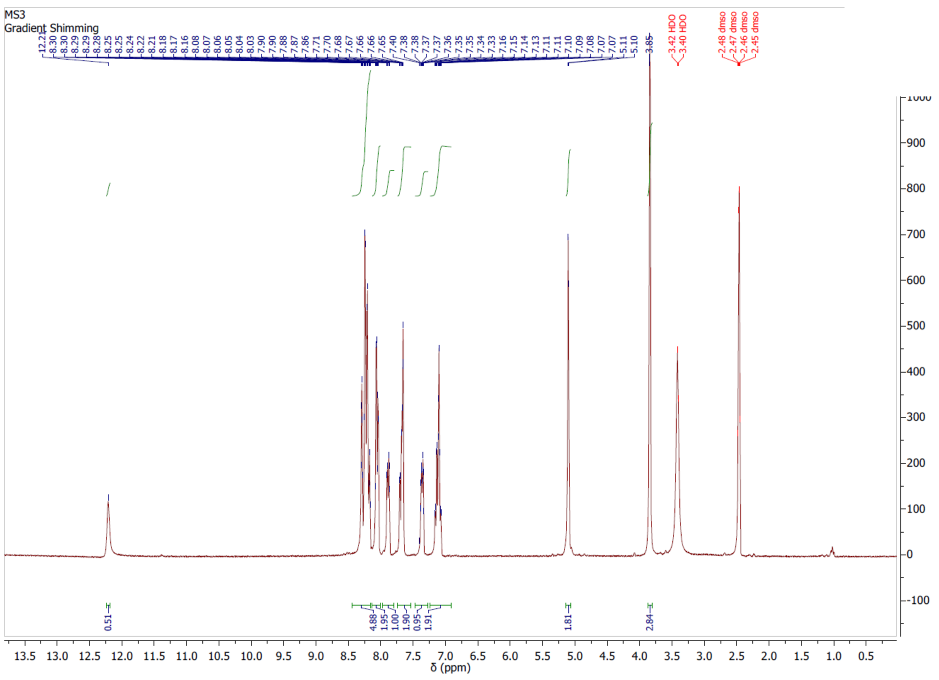

Figure S83. ^{1}H NMR of compound **43oV**.

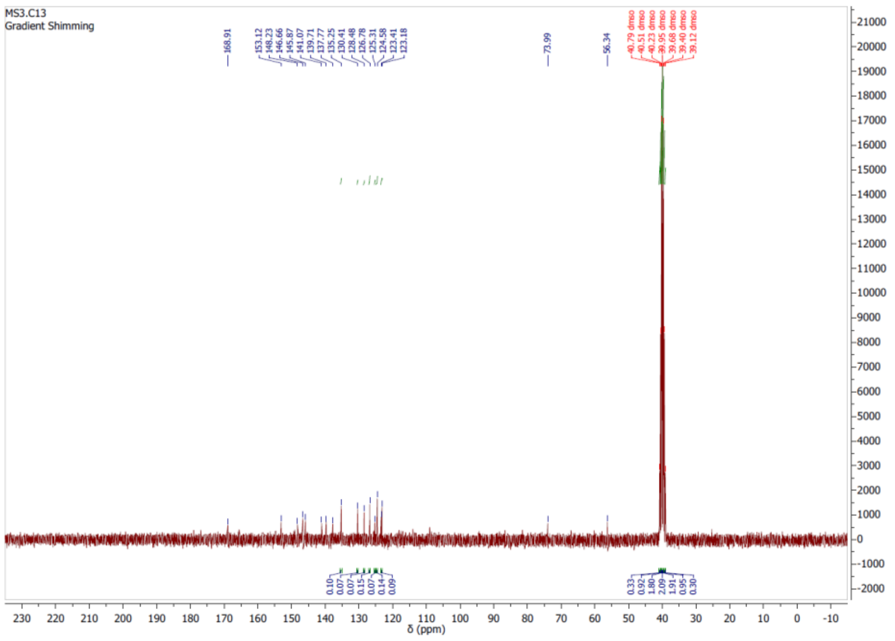

Figure S84. ^{13}C NMR of compound **43oV**.

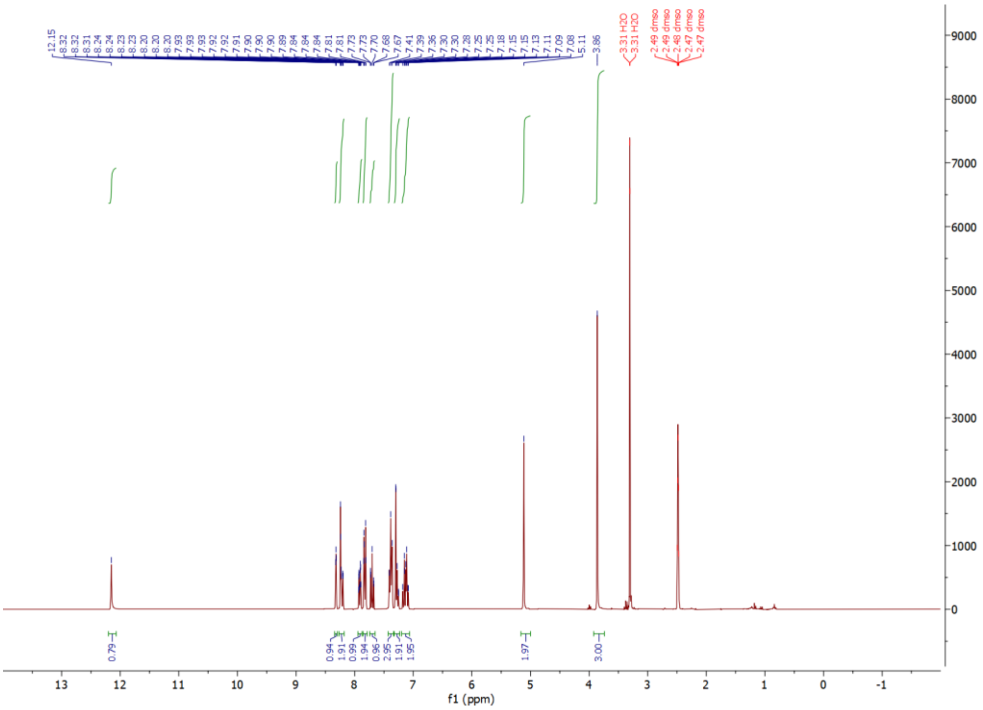

Figure S85. ^{1}H NMR of compound **44oV**.

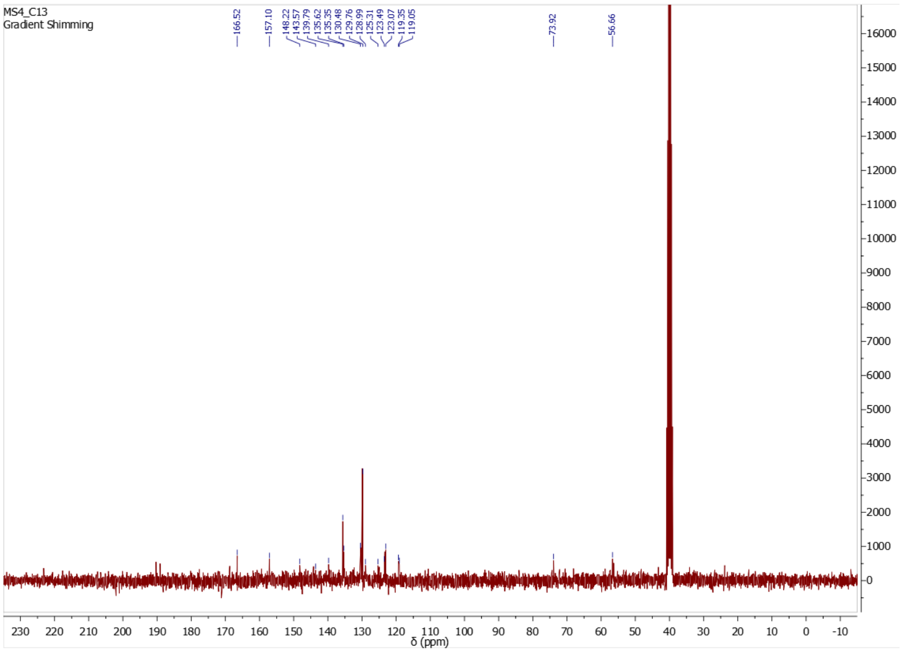

Figure S86. ^{13}C NMR of compound **44oV**.

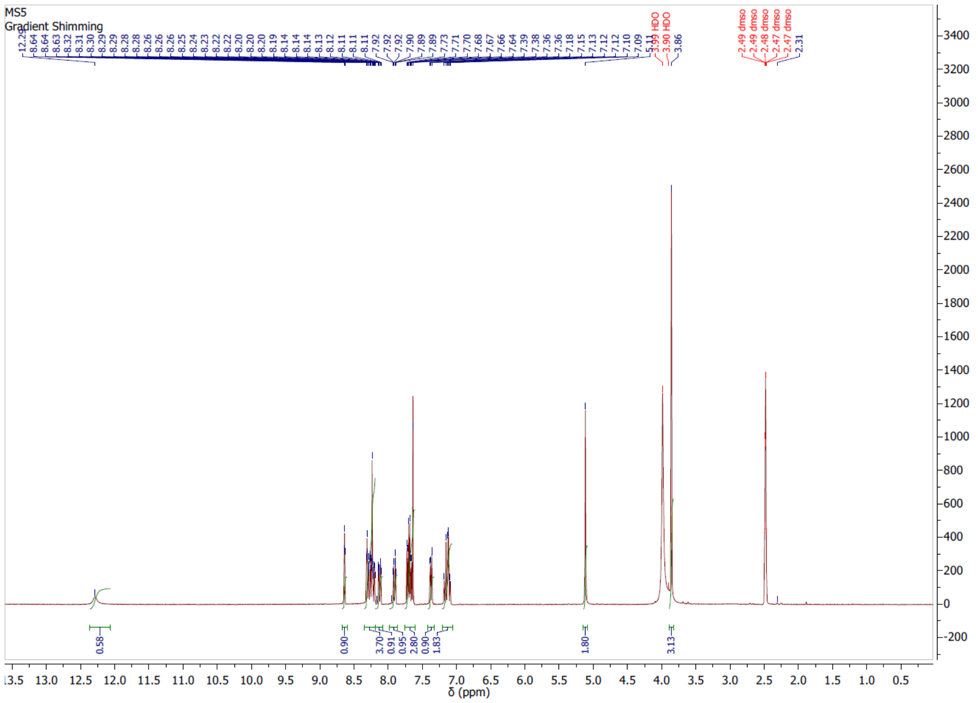

Figure S87. ^{1}H NMR of compound **45oV**.

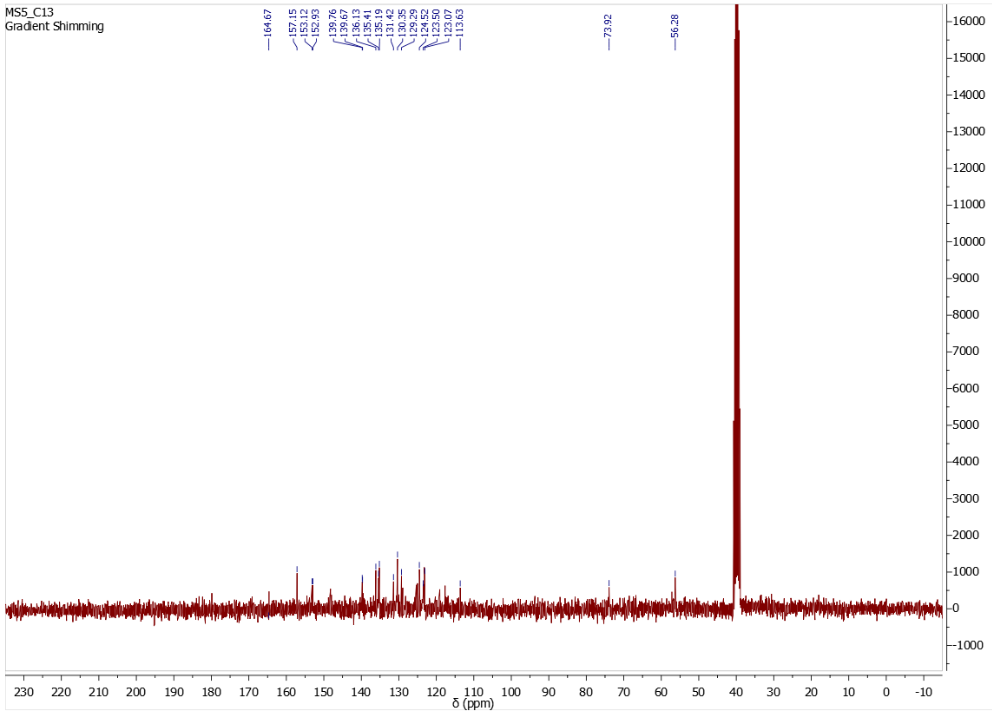

Figure S88 ^{13}C NMR of compound **45oV**.

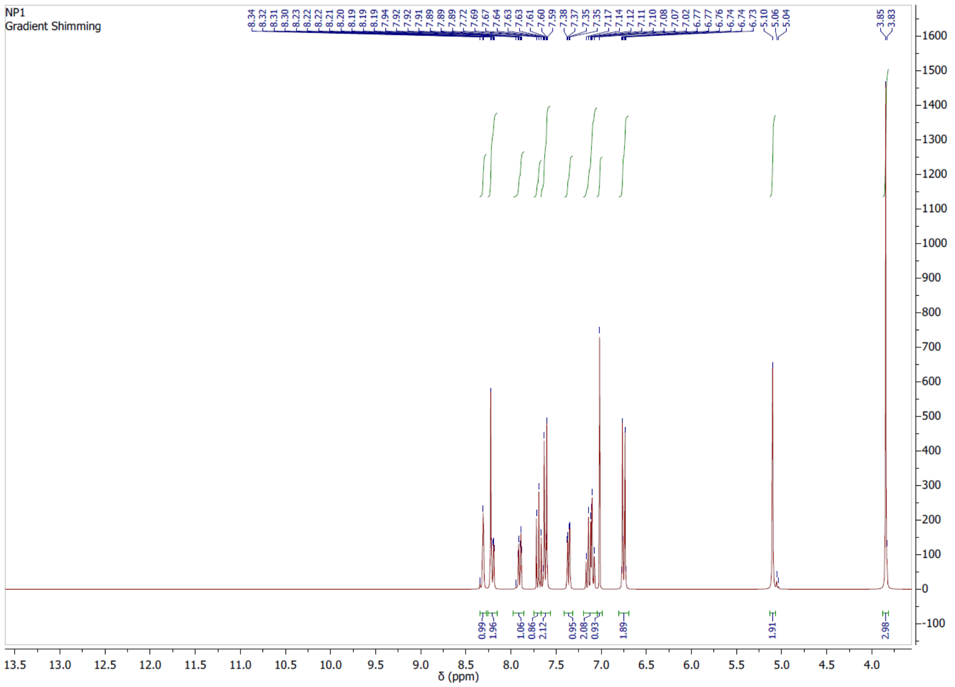

Figure S89. ^{1}H NMR of compound **46oV**.

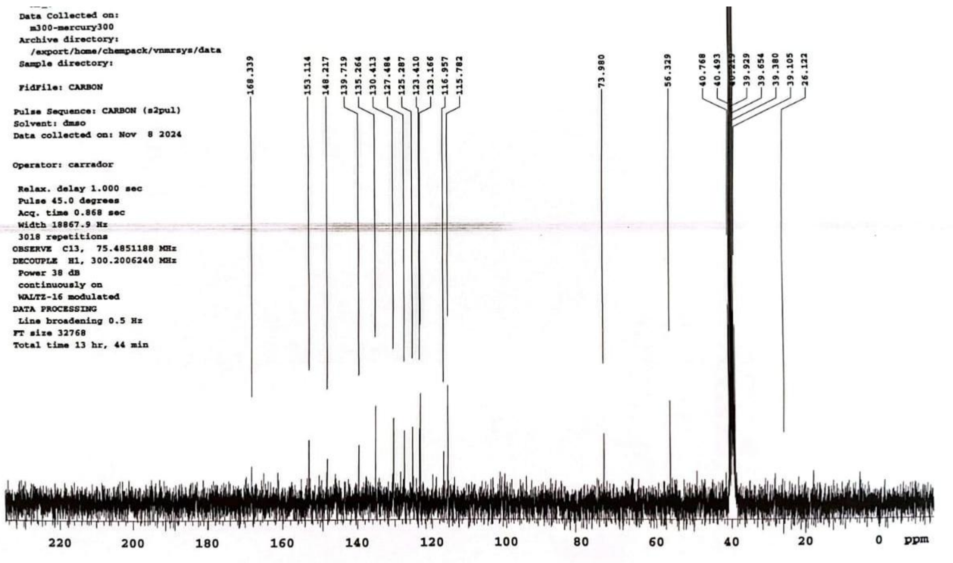

Figure S90. ^{13}C NMR of compound **46oV**.

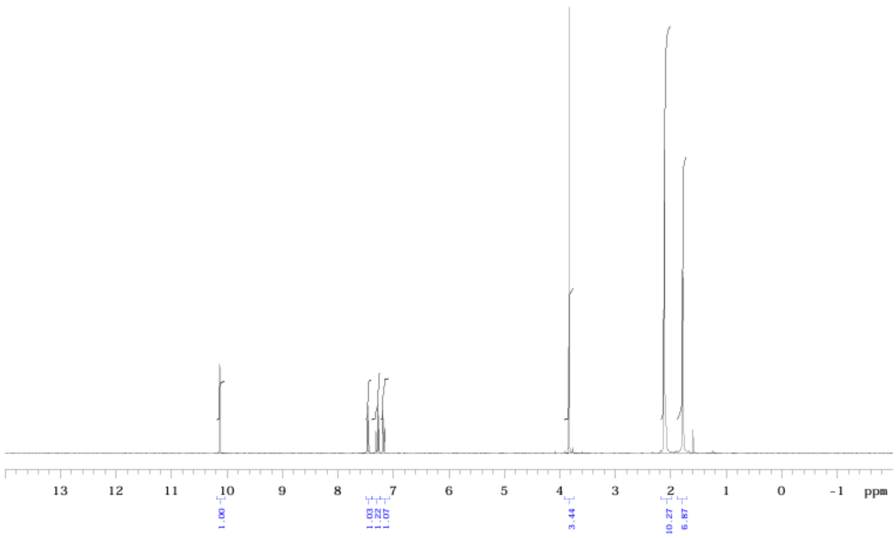

Figure S91 ^{1}H NMR of compound **55oV**.

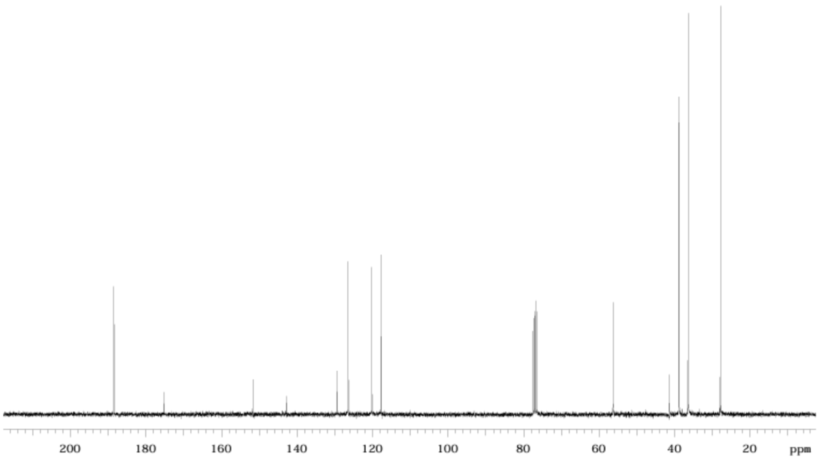

Figure S92. ^{13}C NMR of compound **55oV**.
